# Expression of temperature-sensitive ion channel TRPM8 in sperm cells correlates with vertebrate evolution

**DOI:** 10.7717/peerj.1310

**Published:** 2015-10-13

**Authors:** Rakesh Kumar Majhi, Somdatta Saha, Ashutosh Kumar, Arijit Ghosh, Nirlipta Swain, Luna Goswami, Pratyush Mohapatra, Apratim Maity, Vivek Kumar Sahoo, Abhishek Kumar, Chandan Goswami

**Affiliations:** 1 School of Biological Sciences, National Institute of Science Education and Research, Institute of Physics Campus, Bhubaneswar, Orissa, India; 2 School of Biotechnology, KIIT University, Bhubaneswar, Orissa, India; 3 Department of Zoology, Government Science College, Chatrapur, Ganjam, Odisha, India; 4 Department of Veterinary Biochemistry, CVSc & AH, Orissa University of Agriculture & Technology, Bhubaneswar, Orissa, India; 5 Department of Genetics & Molecular Biology in Botany, Institute of Botany, Christian-Albrechts-University at Kiel, Kiel, SH, Germany; 6 Division of Molecular Genetic Epidemiology, German Cancer Research Center (DKFZ), Heidelberg, BW, Germany

**Keywords:** Sperm cells, TRP ion channel, Cold-sensitivity, Molecular evolution, Ca^2+^-signaling, Vertebrate evolution, TRPM8

## Abstract

Transient Receptor Potential cation channel, subfamily Melastatin, member 8 (TRPM8) is involved in detection of cold temperature, different noxious compounds and in execution of thermo- as well as chemo-sensitive responses at cellular levels. Here we explored the molecular evolution of TRPM8 by analyzing sequences from various species. We elucidate that several regions of TRPM8 had different levels of selection pressure but the 4th–5th transmembrane regions remain highly conserved. Analysis of synteny suggests that since vertebrate origin, TRPM8 gene is linked with SPP2, a bone morphogen. TRPM8, especially the N-terminal region of it, seems to be highly variable in human population. We found 16,656 TRPM8 variants in 1092 human genomes with top variations being SNPs, insertions and deletions. A total of 692 missense mutations are also mapped to human TRPM8 protein of which 509 seem to be delateroiours in nature as supported by Polyphen V2, SIFT and Grantham deviation score. Using a highly specific antibody, we demonstrate that TRPM8 is expressed endogenously in the testis of rat and sperm cells of different vertebrates ranging from fish to higher mammals. We hypothesize that TRPM8 had emerged during vertebrate evolution (ca 450 MYA). We propose that expression of TRPM8 in sperm cell and its role in regulating sperm function are important factors that have guided its molecular evolution, and that these understandings may have medical importance.

## Introduction

Precise thermo-sensitivity allowing animals to discriminate very minute temperature changes ranging from warm to hot, cool to extremely cold temperatures is an ability which is highly conserved in the entire animal kingdom (McKemy, 2007). Though thermosensitivity is well established amongst animals, the key molecules involved in such responses and the molecular mechanisms governing them are not clear (Thomas, 1995; McCleskey, 1997; Brauchi, Orio & Latorre, 2004). Recently, TRPM8 has been reported to be an important molecular thermo-sensor as it can be activated by low temperature (McKemy, 2005; Tominaga & Caterina, 2004; Jordt, McKemy & Julius, 2003; De la Peña et al., 2005). TRPM8 is a well-established non-selective cation channel and so far has been reported from several species, yet exclusively in vertebrates. In most cases, TRPM8 is involved in different sensory processes and physiological functions such as detection of cold temperature (between 22–27 °C). TRPM8 has also been recently recognized as the testosterone receptor as it is activated by testosterone (Asuthkar et al., 2015a; Asuthkar et al., 2015b). In addition, TRPM8 can be modulated by certain natural compounds like menthol, ethanol, icilin, eucalyptol, 1,8-cineole and peppermint oil which provide cool sensation (McKemy, 2005; Andersson, Chase & Bevan, 2004; Benedikt et al., 2006; Namer et al., 2005; Takaishi et al., 2012). Often, the cooling sensation mediated by these natural compounds is species specific and context dependent (Andersson, Chase & Bevan, 2004). For example, TRPM8-specific agonist-mediated cation influx via TRPM8 is pH-dependent. The Ca^2+^-influx induced by menthol and icilin through murine TRPM8 can be completely inhibited or reduced at pH 6.3 and 8.0 respectively when compared to the response at pH 7.5 (Behrendt et al., 2004). In agreement with its precise sensory functions, TRPM8 is expressed in a subpopulation of sensory neurons in Dorsal Root Ganglia (DRG), Trigeminal Ganglia (TG) and in taste papillae of higher animals (Abe et al., 2005; McKemy, Neuhausser & Julius, 2002; Peier et al., 2002).

At this time, reports suggest that expression and functional repercussion of TRPM8 is restricted to vertebrates only and its primary function is to conduct Ca^2+^-influx in response to temperature changes, a function which has remained conserved across different species (Myers, Sigal & Julius, 2009). In case of humans and lower species such as *Xenopus*, activation of TRPM8 by low temperature increases intracellular Ca^2+^ concentration within the sensory neurons (McKemy, Neuhausser & Julius, 2002). TRPM8 is also involved in triggering immune response (Medic et al., 2011; Perraud, Knowles & Schmitz, 2004), regulation of body temperature, and causes hyperthermia (Gavva et al., 2012; Seebacher & Murray, 2007; Tajino et al., 2007; Masamoto, Kawabata & Fushiki, 2007; Almeida et al., 2006; Almeida et al., 2012). TRPM8 present in brown adipocytes are involved in the regulation of body temperature and are relevant for the survival of newborns and hibernating mammals (Yamashita et al., 2008). Cold-seeking behavior during systemic inflammation is also regulated by this ion channel (Almeida et al., 2006). TRPM8 also contributes to cold allodynia and neuropathic pain (Caspani et al., 2009). As TRPM8 is involved in the detection and further avoidance (or preference) of certain physical (such as low temperature) and chemical (such as natural compounds) stimuli, activation of TRPM8 poses immense importance on the animal sensory behavior and animal physiology. Sensitivity against both temperature and natural compounds render TRPM8 as an important molecular factor for detection of suitable habitat, ecological niche formation, adaptation, further speciation and evolution; especially in response to certain selection pressure where TRPM8-mediated sensory processes are involved (Huey, 1991; Tajino et al., 2011; Weil et al., 2005).

In spite of TRPM8’s involvement in precise thermo detection, the actual mechanism by which it is activated by temperature is not clear. Exchanging the entire C-terminus of TRPM8 with that of TRPV1 (which acts as a hot channel) reverses hot and cold-sensitivity of the chimeric channels, confirming that a distinct and smaller part located in its C-terminus can act as molecular-sensor for low temperature (Brauchi, Orio & Latorre, 2004; Brauchi et al., 2006). TRPM8 also acts as voltage gated ion channel which becomes activated upon membrane depolarization (Voets et al., 2004; Voets et al., 2007; Nilius et al., 2005). Indeed, a small yet highly conserved region located in the transmembrane region 3 (TM3) of TRPM8 can act as a “voltage-sensor” (Winking et al., 2012). Similarly, a small double cysteine motif is important for TRPM8 channel functions (Dragoni et al., 2006). These reports indicate the involvement of specific small regions rather than the entire TRPM8 channel in different specific functions which are fairly conserved throughout the evolution. Involvement of such small regions in specific functions is also apparent from sequence homology. TRPM8 of different species share modest sequence identity and homology. *Xenopus* TRPM8 shares approximately 70% identity with rat TRPM8 (Myers, Sigal & Julius, 2009). Therefore, these small regions shed light on the mechanistic details and the molecular evolution of this channel. In particular, these small regions can be used as good indicators to understand how these thermosensitive channels detect temperature and conduct Ca^2+^-influx upon activation in different species. Molecular characterization of TRPM8 mutations, particularly in the voltage-sensor regions and different transmembrane regions has also revealed plausible mechanism/s by which thermal and chemical stimuli can modulate this channel (Voets et al., 2007; Nilius et al., 2005).

As TRPM8 is involved in the thermosensation and Ca^2+^-signaling, in this work we have used TRPM8 sequences from different species and have critically analyzed the molecular evolution of this channel. Our results unravel that TRPM8 is conserved throughout vertebrate evolution. We also demonstrate for the first time that molecular evolution of TRPM8 correlates well with its expression in vertebrate sperm cells.

## Materials and Methods

### Sequence retrieval and alignment

The TRPM8 sequences were retrieved from the Ensembl release 73 (Sept 2013) (Flicek et al., 2010; Hubbard et al., 2009) and NCBI database (Wheeler et al., 2006; Wheeler et al., 2007). Details of each gene as well as protein are enlisted (Tables 1 and S1). The sequence alignment was done by using MUSCLE alignment software (Edgar, 2004a; Edgar, 2004b) with its default values. Sequences for Histone H4 (highly conserved protein) and Cytochrome-C (a semi-conserved protein) from different species was downloaded from the Ensembl (release 73) and also from different databases (Sept 2013) (Flicek et al., 2010; Hubbard et al., 2009; Dickerson, 1971). The Histone and Cytochrome-C protein sequences used for this work have been described before (Sardar et al., 2012).

**Table 1:** List of TRPM8 sequences.

	Name of species	Scientific name	Protein id	Length	Source
**List of species having complete sequences**					
1	Human	*Homo sapiens*	NP_076985.4	1104	NCBI
2	Chimpanzee	*Pan troglodytes*	ENSPTRP00000022348	1052	Ensembl
3	Gorilla	*Gorilla gorilla gorilla*	ENSGGOP00000008197	1105	Ensembl
4	Orangutan	*Pongo abelii*	XP_002813060.1	1104	NCBI
5	Gibbon	*Nomascus leucogenys*	XP_003278601.1	1104	NCBI
6	Olive baboon	*Papio anubis*	ABX89284.1	1104	GenBank
7	Red-bellied titi	*Callicebus moloch*	ACA57875.1	1104	GenBank
8	Common marmoset	*Callithrix jacchus*	ABY79104.1	1104	GenBank
9	Bushbaby	*Otolemur garnettii*	ENSOGAP00000012043	1088	Ensembl
10	Dog	*Canis lupus familiaris*	NP_001104239.1	1104	NCBI
11	Panda	*Ailuropoda melanoleuca*	XP_002917995.1	1104	NCBI
12	Horse	*Equus caballus*	XP_001499536.1	1104	NCBI
13	Cow	*Bos taurus*	ENSBTAP00000019509	1104	Ensembl
14	Pig	*Sus scrofa*	XP_003133798.1	1104	NCBI
15	Squirrel	*Spermophilus tridecemlineatus*	ENSSTOP00000013235	1104	Ensembl
16	Guinea pig	*Cavia porcellus*	ACU30144.1	1104	GenBank
17	Rat	*Rattus norvegicus*	NP_599198.2	1104	NCBI
18	Mouse	*Mus musculus*	NP_599013.1	1104	NCBI
19	African elephant	*Loxodonta africana*	XP_003417951.1	1108	NCBI
20	Nine-banded armadillo	*Dasypus novemcinctus*	ACO88994.1	1104	GenBank
21	Tasmanian devil	*Sarcophilus harrisii*	ENSSHAP00000003620	1103	Ensembl
22	Zebra finch	*Taeniopygia guttata*	ENSTGUP00000003679	1087	Ensembl
23	Chicken	*Gallus gallus*	ENSGALP00000039026	1106	Ensembl
24	Xenopus	*Xenopus laevis*	NP_001155066.1	1139	NCBI
**List of species with incomplete/fragmented sequences**					
1.	Wallaby	*Macropus eugenii*	ENSMEUP00000004623	1050	Ensembl
2.	Turkey	*Meleagris gallopavo*	ENSMGAP00000002375	1069	Ensembl
3.	Tree shrew	*Tupaia belangeri*	ENSTBEP00000008744	1104	Ensembl
4.	Tarsier	*Tarsius syrichta*	ENSTSYP00000009832	1064	Ensembl
5.	Sloth	*Choloepus hoffmanni*	ENSCHOP00000006168	1048	Ensembl
6.	Platypus	*Ornithorhynchus anatinus*	ENSOANP00000020169	1096	Ensembl
7.	Pika	*Ochotona princeps*	ENSOPRP00000001346	1103	Ensembl
8.	Oppossum	*Monodelphis domestica*	ENSMODP00000010005	1096	Ensembl
9.	Mouse lemur	*Microcebus murinus*	ENSMICP00000013415	1090	Ensembl
10.	Megabat	*Pteropus vampyrus*	ENSPVAP00000005934	1092	Ensembl
11.	Monkey	*Macaca mulatta*	ENSMMUP00000027950	192	Ensembl
12.	Dolphin	*Tursiops truncatus*	ENSTTRP00000004154	1103	Ensembl
13.	Coelacanth	*Latimeria chalumnae*	ENSLACP00000016045	1044	Ensembl
14.	Anole lizard	*Anolis carolinensis*	ENSACAP00000010017	1103	Ensembl
15.	Alpaca	*Vicugna pacos*	ENSVPAP00000002122	1059	Ensembl

### Construction of the phylogenetic tree

The complete sequences of TRPM8 from different species were retrieved from NCBI database and their accuracy were confirmed from Uniprot and Ensembl databases (Table 1). MUSCLE alignment program was used to align the amino acid sequences of TRPM8 for the purpose of phylogenetic analysis (Edgar, 2004a; Edgar, 2004b). The phylogenetic tree was constructed by the Bayesian approach (5 runs, 7,500,000 generations, 25% burn-in-period, WAG matrix-based model in the MrBayes 3.2 program) (Whelan & Goldman, 2001).

### Calculation of evolutionary time

In order to explore the molecular evolution of TRPM8, the sequences among different classes were compared and number of changes of amino acids per 100 amino acids was calculated by comparing birds with reptiles, fish with reptiles and reptiles with mammals for different available TRPM8 sequences (Dickerson, 1971; Sardar et al., 2012). Human TRPM8 sequence has been considered as the most recent one and, therefore, the evolutionary time reference of human TRPM8 is considered as zero million year. The average changes were calculated and radiations of mammalian TRPM8 were plotted against million years. While calculating radiation of non-human primates, Gibbon, Gorilla, and Olive Baboon were compared with human TRPM8 sequence. For calculating total mammalian radiation, Dog, Guinea Pig, Pig, and Rat sequences were compared with human TRPM8. In these cases, the average value representing the amino acid change/100 amino acids were considered. For calculation of birds with amphibians, Zebra Finch and Chicken TRPM8 were compared with *Xenopus* (Silurana) *tropicalis* TRPM8. Amphibian and mammals were compared by using TRPM8 from *Xenopus* (Silurana) *tropicalis* and Human. For similar analysis, we used histone-4 as a highly conserved protein and Cytochrome-C as a semi-conserved protein (Dickerson, 1971; Sardar et al., 2012; Baxevanis & Landsman, 1996).

### Synteny analysis of TRPM8 genes

We utilized the Ensembl genome browser for building synteny of TRPM8 gene loci from selected vertebrate genomes (Flicek et al., 2012). Additionally, we examined *X. tropicalis* genome using JGI genome browser (accessed on 28 March 2012, http://genome.jgi.doe.gov/help/browser_main.jsf).

### Fragmentation of TRPM8 in different domains and motifs

In order to analyze the degree of conservation of the different domains present in TRPM8, its various structural regions, domains and motifs were analyzed separately (Phelps & Gaudet, 2007; Liu et al., 2006; Erler et al., 2006; Chuang, Neuhausser & Julius, 2004; Bandell et al., 2006; Rohács et al., 2005; Tsuruda, Julius & Minor, 2006) (Table 4). The N- and C-terminal as well as TM regions and the respective loop regions were considered as mentioned before (Phelps & Gaudet, 2007; Liu et al., 2006; Rohács et al., 2005). In addition, conservation of specific functional regions such as region involved in self-interaction, trafficking and assembly were also explored (Erler et al., 2006). A specific region involved in tetramerization was also analyzed (Phelps & Gaudet, 2007). In all cases, the human TRPM8 sequence was used as the template. Specific domain and motif sequences described for other species were used as query in order to find the corresponding regions present in the human TRPM8 and also in TRPM8 sequences from different species.

MUSCLE software was used to align and find out the respective regions present in other species (Edgar, 2004a; Edgar, 2004b). The aligned data were subsequently imported into “R” statistical tool for statistical analysis. As the complete TRPM8 sequences from certain species are not available (mostly due to sequencing errors at certain regions), the analysis aimed to understand the conservation of different domains and motifs of TRPM8 were conducted with the available full-length sequences only (Table 1). We omitted incomplete sequences in cases where full-length sequences are needed. In all cases, the distance matrix generation and statistical tests were performed as described before (Sardar et al., 2012; Tamura et al., 2011; Kruskal & Wallis, 1952).

#### Circos analysis

The protein sequences of TRPM8 from various species (*n* = 24; Human to *Xenopus*) were collected and aligned with Clustal Omega (http://www.ebi.ac.uk/Tools/msa/clustalo/). The Multiple Sequence Alignment (MSA) generated by it was subsequently fed to the MISTIC server (http://mistic.leloir.org.ar/index.php). In absence of any authentic TRPM8 structure (it doesn’t have any PFAM entry and any structural information entry in RCSB PDB), the hTRPM8 sequence was taken as reference in the MISTIC server.

### Creating the catalogue of TRPM8 variants in 1092 human genomes

TRPM8 variants were sorted from 1092 human genomes (14 different populations) available in 1000 genomes project (Altshuler et al., 2012). Sorting Intolerant From Tolerant (SIFT) is a software tool that predicts whether an amino acid substitution affects protein function and it helps in prioritize substitutions for further study (Ng & Henikoff, 2003). Polymorphism Phenotyping v2 (PolyPhen-v2) is a tool that predicts possible impact of an amino acid substitution on the structure and function of a human protein using straightforward physical and comparative considerations (Adzhubei et al., 2010). Evaluation of the TRPM8 variant impact on human protein was performed using these two methods. We combined impact of these TRPM8 variants by using SIFT (Ng & Henikoff, 2003) and PolyPhen V2 (Adzhubei et al., 2010) tools (Table S2).

**Table 2:** Comparison of TRPM8 proteins for selected vertebrates using percentage identity.

	Chimpanzee	Chicken	Xenopus	Turtle	Lizard	Coelacanth
Human	95.1	80.8	73.5	80.5	81.5	52.8
Chimpanzee		76.5	69.8	76.1	77.6	49.2
Chicken			73.4	84.3	84.1	53.7
Xenopus				74.9	73.9	52.4
Turtle					84.5	51.9
Lizard						53.6

#### Calculation of Grantham Distance

The variations in amino acid sequence of TRPM8 as revealed in 1064 humans genome sequence database was used for this calculation. All these changes in a single amino acid coordinate were analysed and fed to Align GVGD (https://www.agvgd.iarc.fr), an online tool by IARC, WHO which combines biophysical characteristics of amino acids and protein multiple sequence alignments to predict where amino acid substitutions in protein of interest fall in a spectrum from enriched deleterious to enriched neutral. Align-GVGD is an extension of the original Grantham difference to multiple sequence alignments and true simultaneous multiple comparisons. Thus, we obtained the GD (Grantham Deviation) score for each amino acid substitution in TRPM8. The GD scores were plotted against the respective amino acid coordinates and different color codes were given for different classifiers and plotted. The same variants ere also analyzed by SIFT and PolyPhen.

#### Immunohistochemistry of tissues

Tissues were fixed with 4% Paraformaldehyde immediately after surgical removal. After 1 day of fixation, the tissues were transferred to 25% Sucrose and stored at 4 °C till cryo-sectioning. Just before cryo-sectioning, the tissues were snap frozen in dry ice and were then mounted on to the object plate holder of cryostat by embedding solution (Leica Biosystems). The object plate holder was then attached to the object head maintained at −19 °C. The chamber was maintained at −20 °C. Sections of 25 µm thickness were cut using CM3050 S cryostat (Leica Biosystems, Nussloch, Germany). The sections were mounted onto slides pre-coated with 0.1% Ploy-L-Lysine (Sigma-Aldrich, St. Louis, Missouri, USA). The slides were kept frozen at −20 °C freezer till processing. For immunohistochemistry (IHC), the slides were brought to room temperature and washed thrice with 1X PBS. The sections were permeabilized with 0.5% Titron X 100 (Sigma-Aldrich) for 30 min at room temperature, blocked with 5% BSA in PBS for 45 min and then incubated with primary antibodies against TRPM8 (Alomone Labs, Jerusalem, Israel) at 1:300 dilution in 2% BSA overnight in moist chamber at 4 °C. The slides were then washed thrice with 0.1% PBS-T (PBS with 0.1% Tween20) for 5 min each and then incubated with AlexaFluor 488 labelled anti-rabbit secondary antibody (Molecular Probes, Eugene, Oregon, USA) at 1:750 dilution in 2% BSA for 2 h in moist chamber at room temperature. The sections were then washed thrice with 0.1% PBS-T and incubated with DAPI (5 µg/ml) for 15 min at room temperature. After washing thrice with 0.1% PBS-T, the slices were layered with Fluoromount-G and covered by coverslip (Fisher Scientific). After the samples were dried for 24 h at room temperature, the images were acquired using 63X oil immersion objective LSM 780 Confocal microscope (Carl Zeiss, Oberkochen, Germany). The images were processed using LSM image browser software.

### Collection and isolation of sperm cells

Freshly ejaculated sperms from bovine (*Bos indicus*) were collected from healthy bulls after at least 48 h of sexual abstinence by trained professionals (at the Frozen Semen Bank, Cuttack) by means of artificial vagina. For collection of avian sperm, chicken (*Gallus gallus domesticus*) testis were collected (*n* = 4) from the slaughter house and bought to the laboratory within 15 min. After removing the tunica albuginea (outer covering membrane), the testis was chopped into pieces, smeared and then immediately fixed in 4% PFA. For collection of sperm from reptiles, we used a house lizard (*Hemidactylus leschenaultii*). Sexually mature males (*n* = 3) were collected from institutional campus and sacrificed by cervical dislocation. Testes was dissected out and immediately fixed in 4% PFA. For collection of sperm from amphibians, we used a common toad (*Duttaphrynus melanostictus*). Sexually mature male toads (*n* = 3) were collected from institutional campus and sacrificed by cervical dislocation. Testes were dissected out and immediately fixed in 4% PFA. In case of chicken, lizard and common toad, the testis were smeared and centrifuged at 1,000 RPM for 30 s. The supernatants containing the sperm cells were collected for further analysis. Sperm pellet was obtained by centrifugation at 6,000 RPM for 5 min. Mature sperm from Rohu fish (*Labeo rohita)* were collected as described before (Majhi et al., 2013). In all cases, extreme care was taken to minimize the sufferings and the number of animals used. All experiments were done according to the approval from institutional animal ethics committee of NISER (NISER-IAEC/SBS-AH/07/13/10).

### Immunofluorescence analysis and microscopy

Immunocytochemical analysis of sperm cells were performed as described previously (Majhi et al., 2013). In brief, immediately after collection, sperm cells were fixed with 2% paraformaldehyde (PFA) and were permeabilized with 0.1% Triton X-100 in PBS (5 min). Subsequently, the cells were blocked with 5% bovine serum albumin for 1 h. Rabbit polyclonal anti-TRPM8 antibody (1:300 dilution, Alomone Lab, directed against the pore loop i.e., amino acid residues 917-929) of human TRPM8 and corresponding peptide (SDVDGTTYDFAHC, Alomone Lab) have been used. All primary antibodies were incubated for overnight at 4 °C in PBST buffer (PBS supplemented with 0.1% Tween-20). AlexaFluor-488 labeled anti rabbit (Molecular probes) were used as secondary antibodies (1:1000 dilutions). All images were taken on a confocal laser-scanning microscope (LSM-780, Zeiss) with a 63X-objective and analyzed with the Zeiss LSM image examiner software and Adobe Photoshop.

#### Sperm motility assay

Sperm motility assay was performed using frozen straws from Jersey bull sperm (commercially sold by the Frozen Semen Bank, Cuttack). After thawing the straws for 2 min at 37 °C, the semen from two straws (∼200 µl per straw) was pooled into a 1.5 ml tube and 100 µl of semen was distributed into 3 tubes: (1) control tube without any pharmacological agent, (2) tube containing TRPM8 activator WS12 (10 µM), (3) tube containing TRPM8 inhibitor AMTB (10 µM). The tubes were incubated for 1 h in a water bath maintained at 37 °C. Post one hour, 10 µl of semen was spotted on a glass slide maintained at 37 °C, covered with a fresh coverslip and placed under 100X objective of Olympus (BX51) microscope. Motility of bull sperm in each of the tubes was recorded as a movie file for 1 min each. The movies were compiled using movie maker software as described before (Majhi et al., 2013).

## Results

### TRPM8 is a highly conserved protein evolved during vertebrate evolution ca 390 MYA

**Figure 1:**
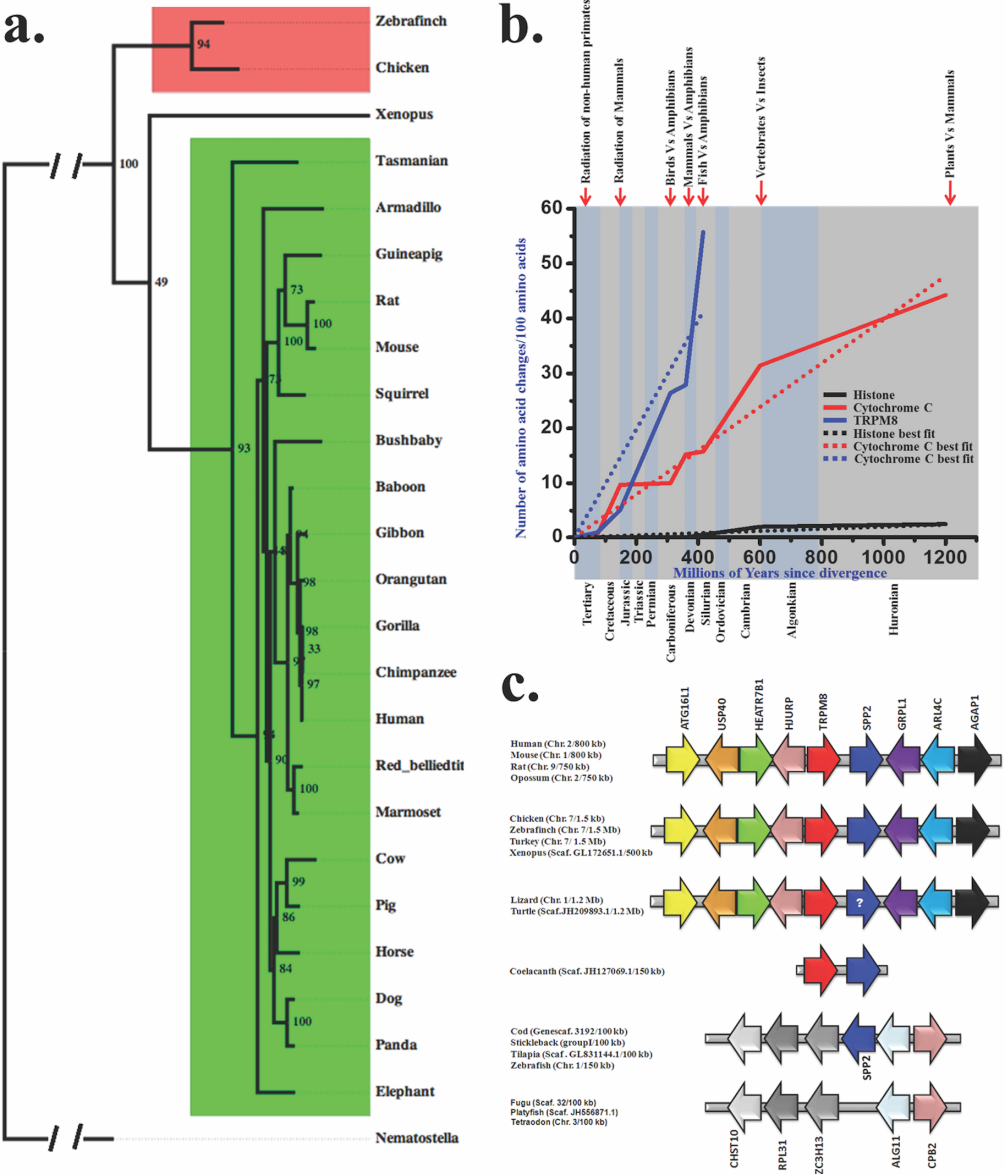
Molecular evolution of TRPM8. (A) Bayesian phylogeny of TRPM8 illustrates that there is a single copy of this gene which is conserved across different vertebrates. Bayesian phylogenetic tree of TRPM8 proteins from mammals (green) and birds (red) was generated using MrBayes 3.2 (Winking et al., 2012). Percentage posterior probabilities are marked at the node of the branches while mean branch length is marked in decimal on the respective branch. TRPC7 gene from *Nematostella vectensis* (JGI acc. id—estExt_fgenesh1_pg.C_6220005) served as out-group in this phylogenetic tree. (B) Conservation analysis with TRPM8 in comparison to histone H4 (a conserved protein) and Cytochrome-C (a semi-conservative protein) are shown here. The values for histone-H4 and Cytochorome-C are plotted as best-fit while values for TRPM8 are plotted according to the original values. (C) Synteny analysis of TRPM8 indicates that TRPM8 and SP2 are juxtaposed throughout the vertebrate evolution. (In case of turtle and lizard, the presence of SPP2 is not confirmed yet (indicated by a question mark) as genome assembly of lizard and turtle are still in draft conditions).

We explored the molecular evolution of TRPM8 in details and therefore retrieved full-length or partial TRPM8 sequences from different databases (Table 1). Full-length protein sequences were used for the establishment of bayesian phylogenetic history, which depicts that there is a single copy of TRPM8 which is conserved in different vertebrates with high statistical support (Whelan & Goldman, 2001) (Fig. 1A). Furthermore, we calculated the evolutionary age and pattern of TRPM8. For that purpose we estimated the number of amino acid changes per 100 sites among different TRPM8 sequences (Fig. 1B) as reported previously (Dickerson, 1971; Sardar et al., 2012; Baxevanis & Landsman, 1996). This analysis also indicates that TRPM8 has evolved during the Devonian era (approximately 390 MYA) when amphibians started evolving from fishes. The evolutionary slope of TRPM8 indicates that it was subjected to different levels of selection pressure in different era. The variations became lower during radiation of mammals and lowest during the radiation of non-human primates. In the cretaceous era, TRPM8 incorporated few changes indicating a molecular stabilization process coinciding with the radiation of mammals. During the tertiary era, TRPM8 incorporated minor changes supporting the notion that TRPM8 structure-function relationship is fairly stabilized during the primate evolution.

### Emergence of TRPM8 co-relates with vertebrate evolution

In order to understand the conservation of TRPM8 in the genome of different species throughout the evolution, we performed synteny analysis (Fig. 1C). Interestingly, TRPM8 gene has been detected in coelacanth (*Latimeria chalumnae*, a fish close to mammals and reptiles). Human TRPM8 shares 80.2%, 73.5%, 81.5% and 52.8% identities with its orthologs in chicken, frog, lizard and coelacanth, respectively (Table 2). To evaluate whether invertebrates have TRPM8, we carried out extensive survey of TRPM8 gene using BLAST suite. Majority of hits picked up were hypothetical or without proper annotation and hence we picked up sequences from few selected invertebrates only. We report that TRPM-like genes exist in invertebrate genomes which are not genuine TRPM8 orthologs (Table S1). These TRPM-like genes from hydra to lancelet share 22.3–27.1% of identities with human TRPM8 (Table 3). A comparative phylogentic tree also demonstrates that genuine TRPM8 orthologs are not present in invertebrates, but these species possess distinct homologs such as TRPM2 or TRPM3-like genes (Fig. S1).

**Table 3:** Comparison of sequence identities of invertebrate TRPM-like sequences with human TRPM8.

Species	Accession ID	Capitella	Branchiostoma	Trichoplax	Aplysia	Strongylocentrotus	Hydra	Bombyx	Acyrthosiphon	Crassostrea	Danaus	Ceratitis
Human	NP_076985.4	**25.9**	**27.1**	**25.5**	**23.8**	**22.3**	**22.5**	**25.7**	**24.1**	**24.1**	**21.6**	**23.9**
Capitella	ELU00429.1		35.4	31.2	21.1	22.3	25.9	24.2	26.3	28.5	23.5	26.1
Branchiostoma	XP_002592165.1			29	21.9	20.2	26	23.4	27.3	31.2	21.5	25.6
Trichoplax	XP_002114617.1				30.1	32.5	25.6	28	26.8	24.9	24.8	27.9
Aplysia	XP_005094347.1					24.2	23.7	22.1	24.5	22.5	21.3	24.2
Strongylocentrotus	XP_792292.3						23.4	22.6	24.3	21.4	19.9	24.9
Hydra	XP_002168052.2							26.9	26.3	23.2	23.2	26
Bombyx	XP_004933523.1								65.9	24.2	68.4	67.6
Acyrthosiphon	XP_001950420.2									23.5	52	63.1
Crassostrea	EKC31999.1										18.4	23.4
Danaus	EHJ78405.1											56.5

**Table 4:** Description of different domains and motifs.

Region	Amino acids	Refs
MHR1	117-245	Wheeler et al. (2006)
MHR2	246-352	Wheeler et al. (2006)
MHR3	353-550	Wheeler et al. (2006)
MHR4	551-692	Wheeler et al. (2006)
TM-1	691-710	Wheeler et al. (2007)
Loop-1	711-730	Wheeler et al. (2007)
TM-2	731-758	Wheeler et al. (2007)
Loop-2	759-802	Wheeler et al. (2007)
TM-3	803-820	Wheeler et al. (2007)
Loop-3	821-829	Wheeler et al. (2007)
TM-4	830-848	Wheeler et al. (2007)
Loop-4	849-866	Wheeler et al. (2007)
TM-5	867-885	Wheeler et al. (2007)
Pore region	886-954	Wheeler et al. (2007)
TM-6	955-977	Wheeler et al. (2007)
N-terminal	1-692	Wheeler et al. (2006)
TM and loop region	693-989	Wheeler et al. (2006)
C-terminal	978-1104	Wheeler et al. (2007)
Regions required for channel localization and tetramerization	40-86	Wheeler et al. (2006)
Coiled coil region at the N-terminus	594-628	Edgar (2004a)
Critical region for channel gating	799-805	Phelps & Gaudet (2007); Edgar (2004b); Dickerson (1971)
Region important for voltage sensing	842-856	Brauchi et al. (2006)
TRP-domain	990-1025	Wheeler et al. (2006)
TRP-domain	993-1016	Sardar et al. (2012)
TRP-Box	993-998	Sardar et al. (2012)
Self-interacting sites	1007-1047	Edgar (2004a)
Coiled coil region at the C-terminus	1064-1104	Whelan & Goldman (2001)
Coiled coil region at the C-terminus	1070-1104	Wheeler et al. (2006)

We found that since ∼400 MYA (a time relevant for vertebrate evolution), TRPM8 has shared close relationship with a 24 kDa protein named secreted phosphoprotein 2 (SPP2). To evaluate the level of gene-specific functional constraints of TRPM8 in vertebrate evolution in more details, we mapped chromosomal locus of these two genes (TRPM8 and SPP2) in the representative vertebrate genomes. We found that TRPM8 and SPP2 genes are juxtaposed in a head to tail orientation on the human chromosome 2, flanked by a tetrad of autophagy related 16-like 1 (ATG16L1), ubiquitin specific peptidase 40 (USP40), HEAT repeat containing 7B1 (HEATR7B1), Holliday junction recognition protein (HJURP) on the one side. On the other side, we found a dyad of ADP-ribosylation factor-like 4C (ARL4C), ArfGAP with GTPase domain, ankyrin repeat and PH domain 1 (AGAP1) as conserved arrangements. Notably, this locus is conserved in all mammals tested, i.e., in human (chromosome 2), mouse (chromosome 1), rat (chromosome 9) and oppossum (chromosome 2) (Fig. 1C). Furthermore, we identified the same locus in chicken, zebra finch and turkey where these two genes are present on the chromosome 7. In reptiles also, this locus seem to be maintained. Amphibians, namely *Xenopus* have this locus on scaffold GL172651.1. Interestingly, coelacanth (*Latimeria chalumnae*), a fish close to mammals and reptiles, has this locus maintained. But this locus is not found in any other known fish genomes available so far. However, Cod, stickleback, tilapia and zebrafish possess SPP2 at another locus flanked by a dyad of zinc finger CCCH-type containing 13 (ZC3H13), ribosomal protein L31 (RPL31) and carbohydrate sulfotransferase 10 (CHST10) on the one side while on the other side a dyad of carboxypeptidase B2 (CPB2) and asparagine-linked glycosylation 11, alpha-1,2-mannosyltransferase homolog (ALG11) is conserved. This altered locus is present in Fugu, platyfish and tetraodon, but does not contain SPP2 gene. Overall, adjunct chromosomal location of TRPM8 and SPP2 from coelacanth to mammals suggests the level of gene-specific functional constraints of these two genes in vertebrate evolution. Though the other fishes have no TRPM8 gene and corresponding locus; SPP2 gene is present in selected fishes on other locus. This probably suggests that TRPM8 gene was lost specifically in ray-finned fishes after separation from basal fishes at about 450 MYA. As SPP2 is a morphogen involved in bone formation and the conservation of these two genes in the same genomic locus for last 390 million years strongly suggests that the *gene-specific functional constraints* of these two gene products are most likely for some common function.

### Different regions of TRPM8 have evolved with different selection pressure

**Figure 2:**
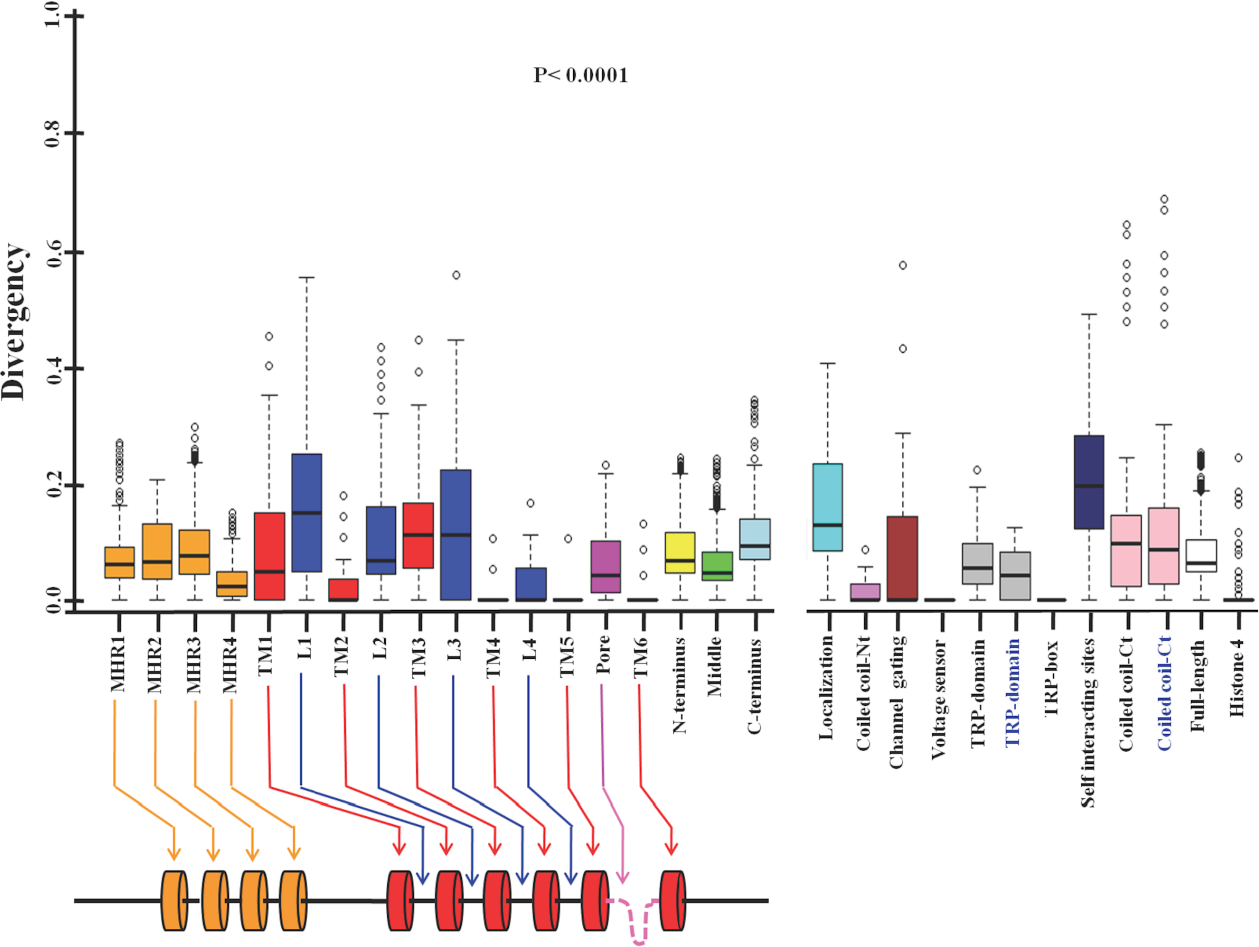
Different domains, motifs and interacting sites of TRPM8 had different evolutionary selection pressure. The lower value indicates more conservation and higher value indicates less conservation. Different regions of the TRPM8 are indicated by different colors. MHR1, TRPM homology regions; TM, Transmembrane region; L, Loop region; Pore, Pore loop; TRP-box, Signature motif for TRP-box; Localization, regions required for channel localization and tetramerization; Coiled coil-Nt, Coiled coil region at the N-terminus; channel gating, Critical region for channel gating; Voltage-sensor, Region important for voltage sensing; TRP domain, TRP domain of TRPM8; self-interacting sites, regions required for self interaction; coiled coil-Ct, Coiled coil region at the C-terminus; N-terminus, N-terminal cytoplasmic domain; Middle, Middle portion containing all the TM and loop regions; C-terminus, C-terminal cytoplasmic domain of TRPM8; FL, Full-length; Histone 4, Histone 4. TRP-domain and coiled-coil-Ct (indicated in black and blue script) represent TRP-domain of TRPM8 and Coiled coil region at the C-terminus which are over-lapping but different sequences as described in Table 4. The schematic drawing of TRPM8 (below) represents different domains and motifs (not according to scale). All values are significant (*P* < 0.0001, Kruskal–Wallis test).

Next we tested the conservation in different motifs and domains of TRPM8 (Fig. 2, Table 4) (Phelps & Gaudet, 2007; Liu et al., 2006; Erler et al., 2006; Chuang, Neuhausser & Julius, 2004; Bandell et al., 2006; Rohács et al., 2005; Tsuruda, Julius & Minor, 2006). We noted that all four TRPM homology regions (MHR) are well conserved throughout evolution. This is in full agreement with the previous analysis, which suggested that MHR regions are conserved in all members of the TRPM family (Phelps & Gaudet, 2007). The MHR4 is most conserved followed by MHR1, MHR2 and MHR3. Among all the TM regions, TM-4, TM-5 and TM-6 reveal highest level of conservation respectively. TM-2 is also well conserved and this agrees well with the fact that this region is involved in the interaction with specific agonist such as menthol (Malkia et al., 2009). However, TM1 and TM3 show more variability compared to all other TM regions suggesting that these two TM regions have less functional importance than TM-4, TM-5 and TM-6. Among all the loops, loop-4 is highly conserved and loop-1 as well as loop-3 are least conserved. Loop-2 is also less conserved when compared with loop-4. Interestingly, pore loop is less conserved than loop-4, suggesting an important functional contribution of loop-4 in channel gating. The amino acid region 40-86 (located at the N-terminus and required for channel localization and tetramerization) is not well conserved (Phelps & Gaudet, 2007). This may suggest that the importance of this region in the context of channel localization and function is limited to higher mammals only. Notably, the 40 amino acids (AA 1007-1047) located at the C-terminus (has been described as important for self-interaction) is not well conserved (Erler et al., 2006). However, report suggests that this region is functionally important as R1008Q mutant (human TRPM8) is defective in desensitization (Fujita et al., 2013). This may suggest the importance of this region in the higher mammals only. Coiled coil region located at the N-terminus (AA 594-628) is more conserved when compared to the coiled coil region located at the C-terminus (AA 1064-1104) (Phelps & Gaudet, 2007; Erler et al., 2006; Tsuruda, Julius & Minor, 2006). The TRP-domain regions (AA 990-1025 and AA 993-1016) as described by two different groups is less conserved than the TRP-box region (AA 993-998) located within this region (Phelps & Gaudet, 2007; Rohács et al., 2005). Notably the TRP-box is highly conserved region indicating the functional importance of this region in channel function. This conservation accords well with the documented report of PIP_2_ interaction with TRPM8 in this region (Rohács et al., 2005). The voltage-sensing region (AA 842-856) seems to be totally conserved across all the species indicating the functional importance of this region (Voets et al., 2007). In the similar manner, a small region (AA 799-805) required for channel gating is conserved in TRPM8 throughout evolution indicating its importance in the channel function (Voets et al., 2007; Chuang, Neuhausser & Julius, 2004; Bandell et al., 2006).

**Figure 3:**
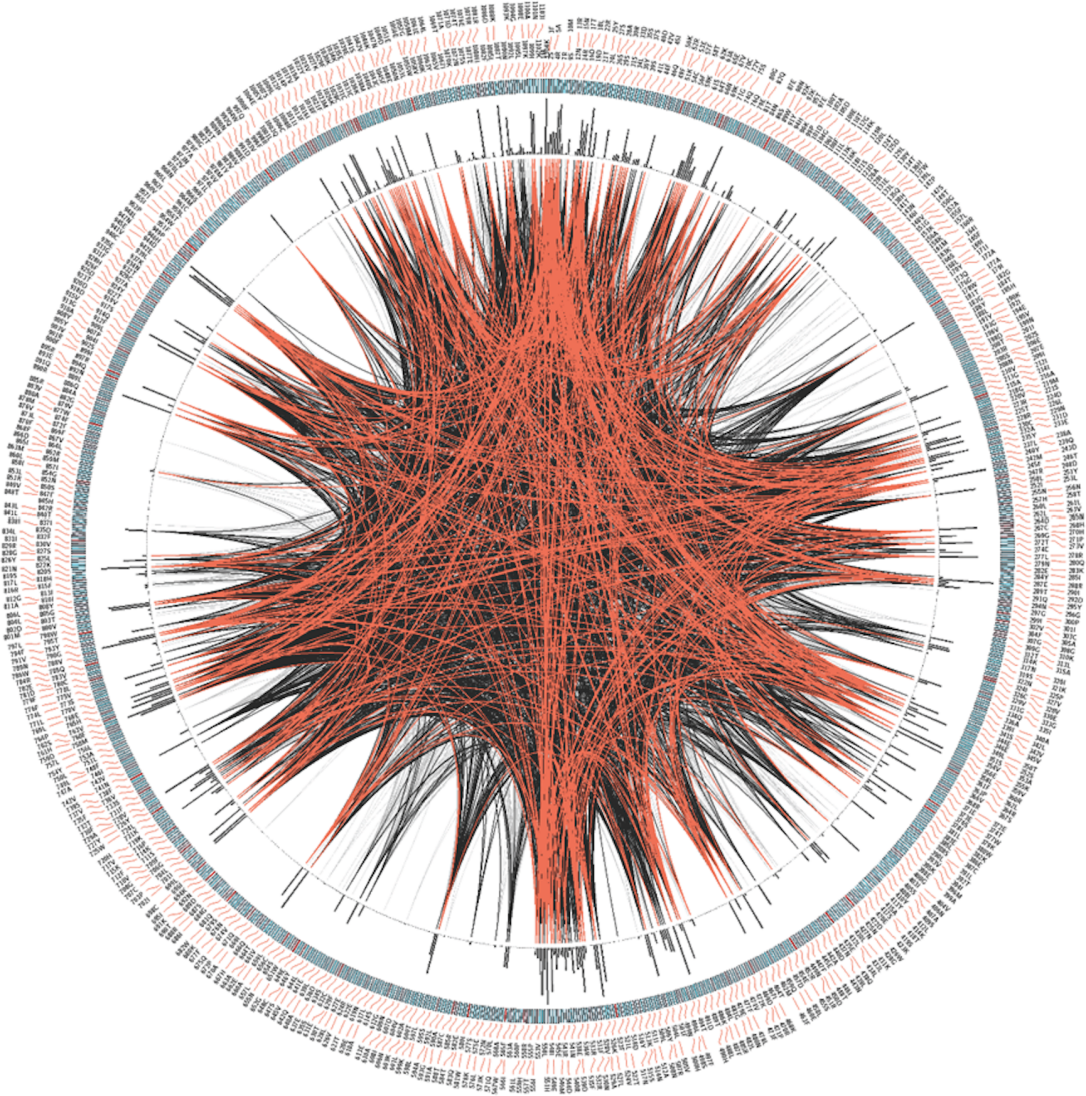
CIRCOS analysis of TRPM8. Mutual Information (MI) Circo of Multiple Sequence Alignment (MSA) analysis of TRPM8. Human TRPM8 protein was used as reference. Labels in the first (outer) circle indicate the alignment position and the amino acid code of the reference sequence. The coloured square boxes of the second circle indicate the MSA position conservation (highly conserved positions are in red, while less conserved ones are in blue). The third circle shows the Cumulative Mutual Information (cMI) as histograms, facing outwards. In the center of the circle lines can be observed, that connect pairs of positions with MI greater than 6.5. Red edges represent the top 5%, black lines represent points scoring between 95–70%, and gray edges account for the remaining 70%.

Next we analyzed the conservation of TRPM8 by using Circos (Fig. 3). This analysis along with the multiple sequence analysis reveals the conservation of TRPM8 in single amino acid resolution, possible intramolecualr interaction sites and also suggests the respective conservation of few amino acids that are responsible for post-translational modifications such as phosphorylation, N-glycosylation and Ubiquitination (Table 5).

**Table 5:** Description of functionally important specific amino-acids.

Position	Type of modification	Region in the protein	Remarks on function and conservation	Species	Reference
Ser-9	PKA Phosphorylation	N-Terminal	This phosphorylation site is critical for Clonidine-induced channel inhibition. This site is conserved in all mammals, but not present in birds, reptiles and coelacanth, changed to Lys in amphibians (*Xenopus*).	Human	Bavencoffe et al., 2010
Thr-17	PKA Phosphorylation	N-Terminal	This phosphorylation site is critical for Clonidine-induced channel inhibition. Conserved in mammals, changed to Asp in birds. Not conserved in other species.	Human	Bavencoffe et al., 2010
Lys-283	Ubiquitination	MHR2	Conserved in mammals, birds, reptiles, amphibians but not in coelacanth.	Mouse	CST Curation Set 3115; Year: 2007
Lys-298	Ubiquitination	MHR2	Conserved in mammals, birds, reptiles, amphibians and coelacanth.	Mouse	CST Curation Set 3115; Year: 2007
Thr-503	Phosphorylation	MHR3	Conserved in mammals, changed to Ser in birds. Not conserved in other species.	Human	CST Curation Set 9059; Year: 2010
Tyr-506	Phosphorylation	MHR3	Conserved in mammals, changed to Phe in birds, reptiles and amphibians and to Ser in coelacanth.	Human	CST Curation Set 9059; Year: 2010
Ser-850	Phosphorylation	Voltage-sensor region	Conserved in all except Coelacanth.	Human	CST Curation Set 2928; Year: 2007
Asn-934	N-glycosylation	Putative pore region	This position is flanked by two conserved residues (Cys-929 and Cys-940) and this glycosylation helps in lipid raft segregation. Mutation in this position (N934Q) results in 50% dissociation of the protein from lipid rafts. This position is highly conserved in all species.	Human, mouse	Dragoni et al., 2006 Morenilla-Palao et al., 2009
Ser-1040	Phosphorylation	Self-interacting site	Conserved in mammals, changed to Pro in others.	Human	CST Curation Set 2262; (2007)
Ser-1041	Phosphorylation	Self-interacting site	Conserved in all species.	Human	CST Curation Set 2262; (2007)

### Catalog of genetic variation in TRPM8 using 1092 human genomes

**Figure 4:**
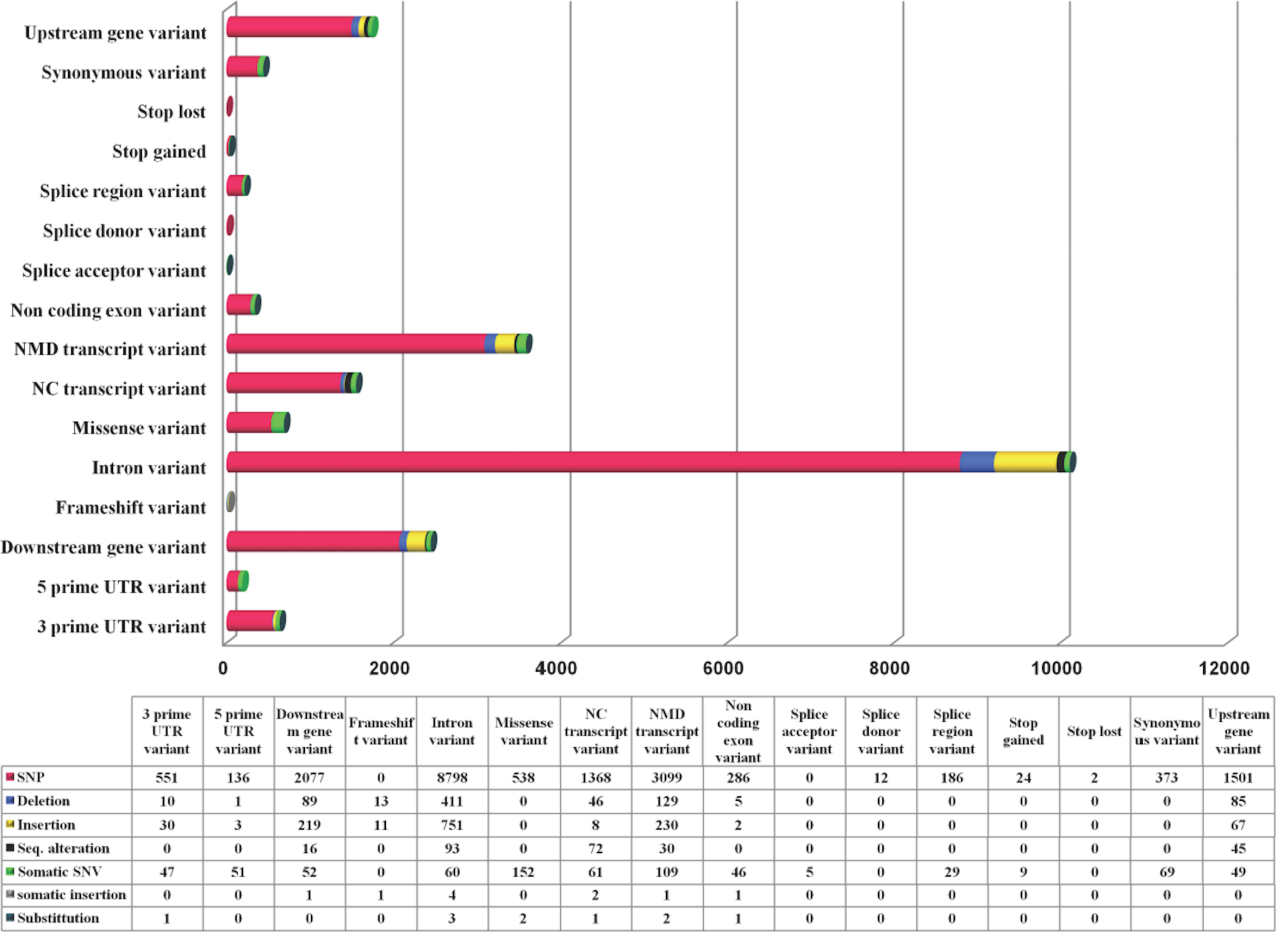
TRPM8 variant analysis from human genome. Human TRPM8 variants computed from 1092 genomes of 14 human populations demonstrates that SNPs are predominant variant class (according to general variant classification).

While TRPM8 remains conserved in most of the mammals, in human it has a large number of variants. We computed variations in the TRPM8 gene in 1092 human genomes as obtained from 14 different populations and details of these studies are provided (Fig. 4). Majority of these are SNPs (85.8%). There are total 16656 variations observed in 16 variant types (based on location in and around TRPM8 gene), with top 5 variant types being intron variants (10116), Nonsense-mediated mRNA decay (NMD) transcript variants (3558), downstream gene variants (2409), upstream gene variants (1747) and non-coding (NC) transcript variant (1556). Top four variant classes (according to general variant classification) are single-nucleotide polymorphism (SNP) variants (85.8%), insertions (6%), deletions (3.6%) and single nucleotide variant (SNV) (3.35%). We further examined 692 missense variations to examine what are the critical changes, i.e., changes which can alter both TRPM8 amino acid sequence and thus secondary structural elements (compiled in Table S2).

**Figure 5:**
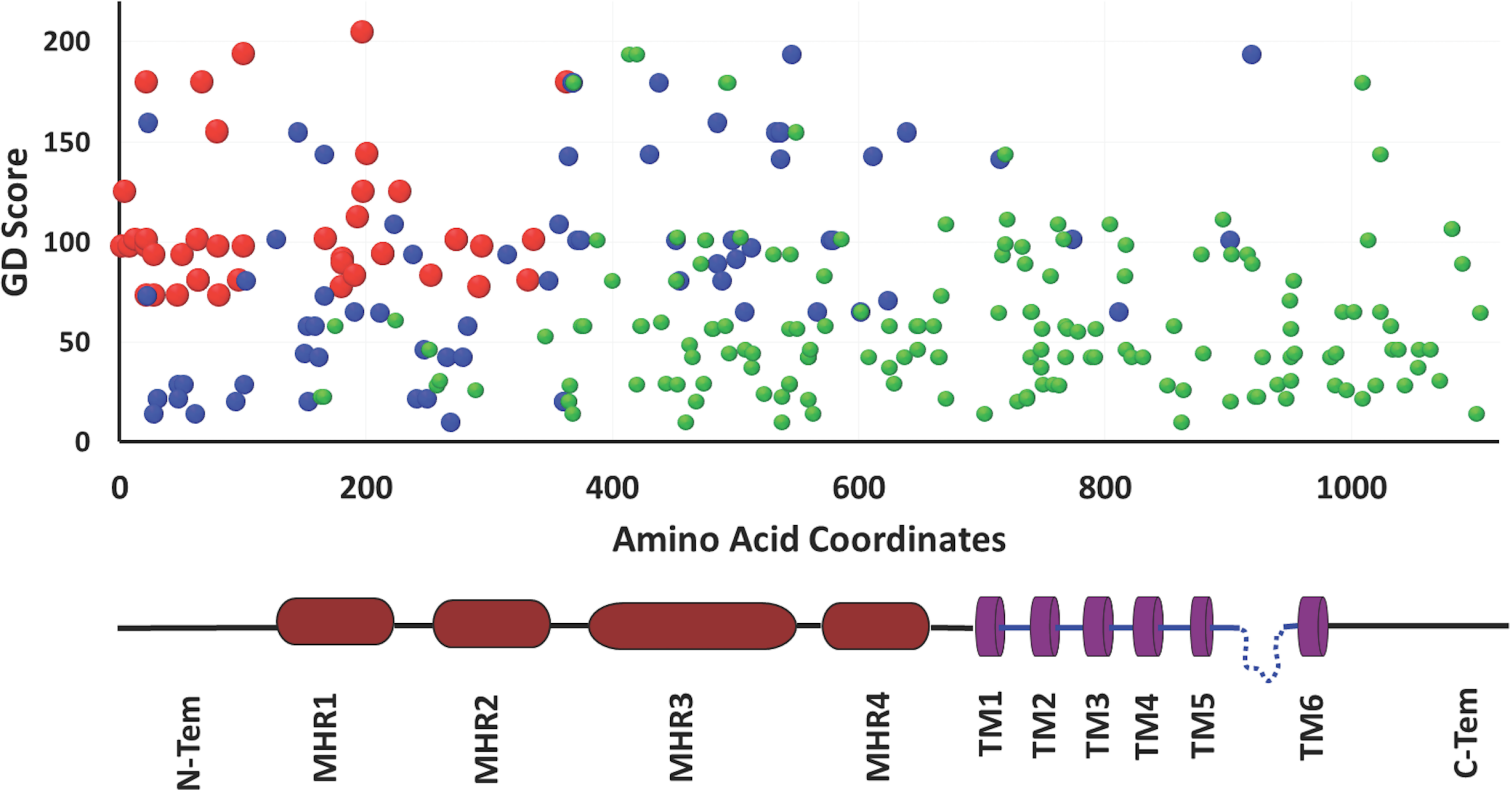
Analysis of hTRPM8 variants. Mutational analysis of TRPM8 ion channel on the basis of Grantham Deviation (GD), SIFT and PolyPhen score. The red spheres denote mutations which are most likely damaging according to all three mutation prediction methods (GD, SIFT and PolyPhen), the blue spheres denote the mutations which are probably damaging according to only two of these methods and the green spheres denote ones which are probably damaging according to only one of these methods. This analysis also suggests that in human population, the N-terminal region of TRPM8 has more variability than its TM or C-terminal regions.

We calculated Grantham Distance (GD) scores of all these missense variants which also suggest that that N-terminal region of TRPM8 contains more variations and majority of these can cause significant structural changes (Fig. 5). The N-terminal region of TRPM8 has more variants compared to its C-terminus (Fig. 5). In general, we noted that in case of the positions that have multiple variations, the variations are not random and a clear biasness for certain amino acids is prominent (Fig. 5).

### TRPM8 is expressed in sperm cell throughout vertebrate evolution

**Figure 6:**
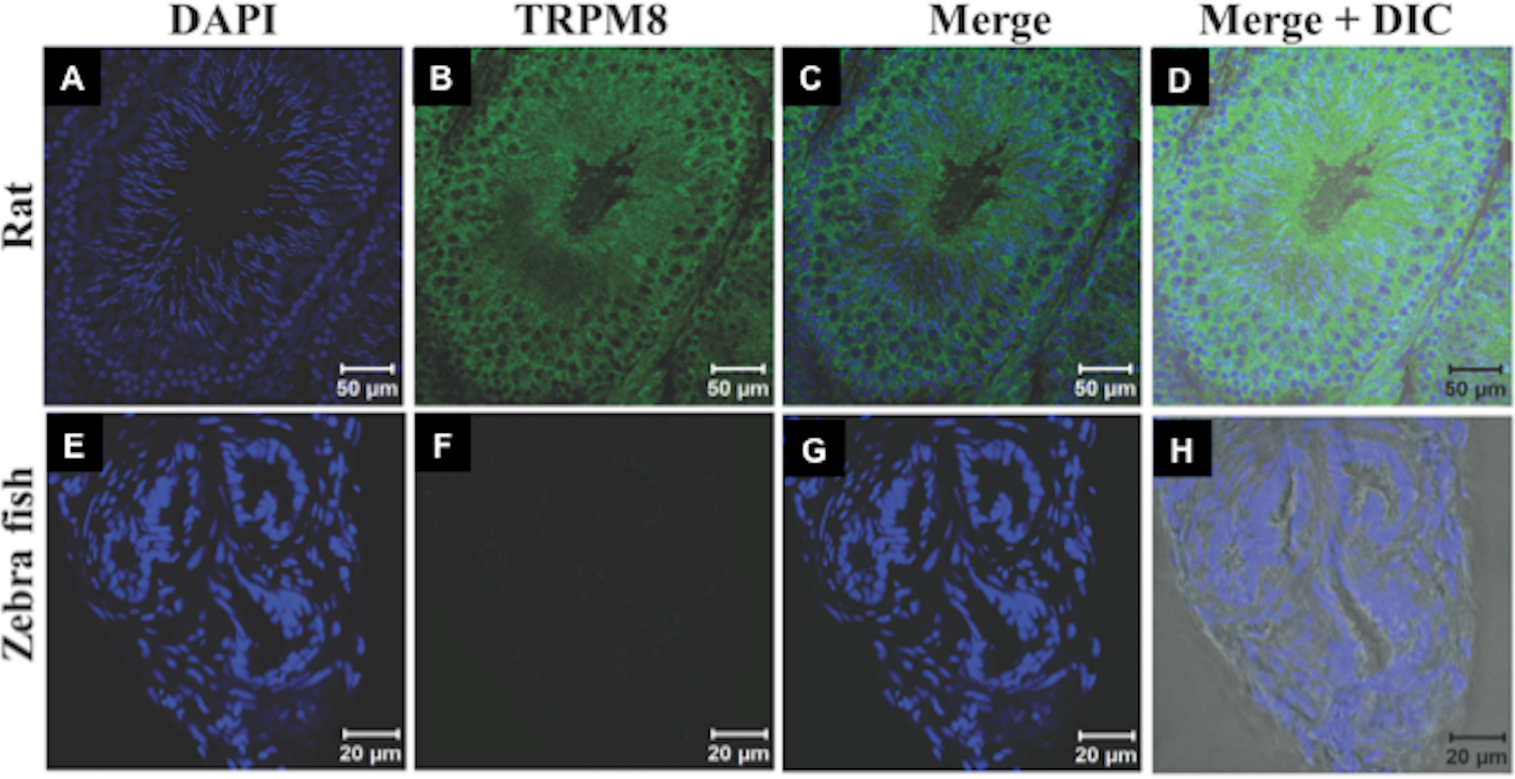
TRPM8 in present in the testis. The confocal images of testis sections are shown from Rat (A–D) and Zebrafish (E–H) stained with an antibody specific for TRPM8 (B–D) and DAPI (Blue). The merged images with DIC are shown in the (D and H). TRPM8 antigen is detected in testis of Rat but not in that of Zebrafish.

**Figure 7:**
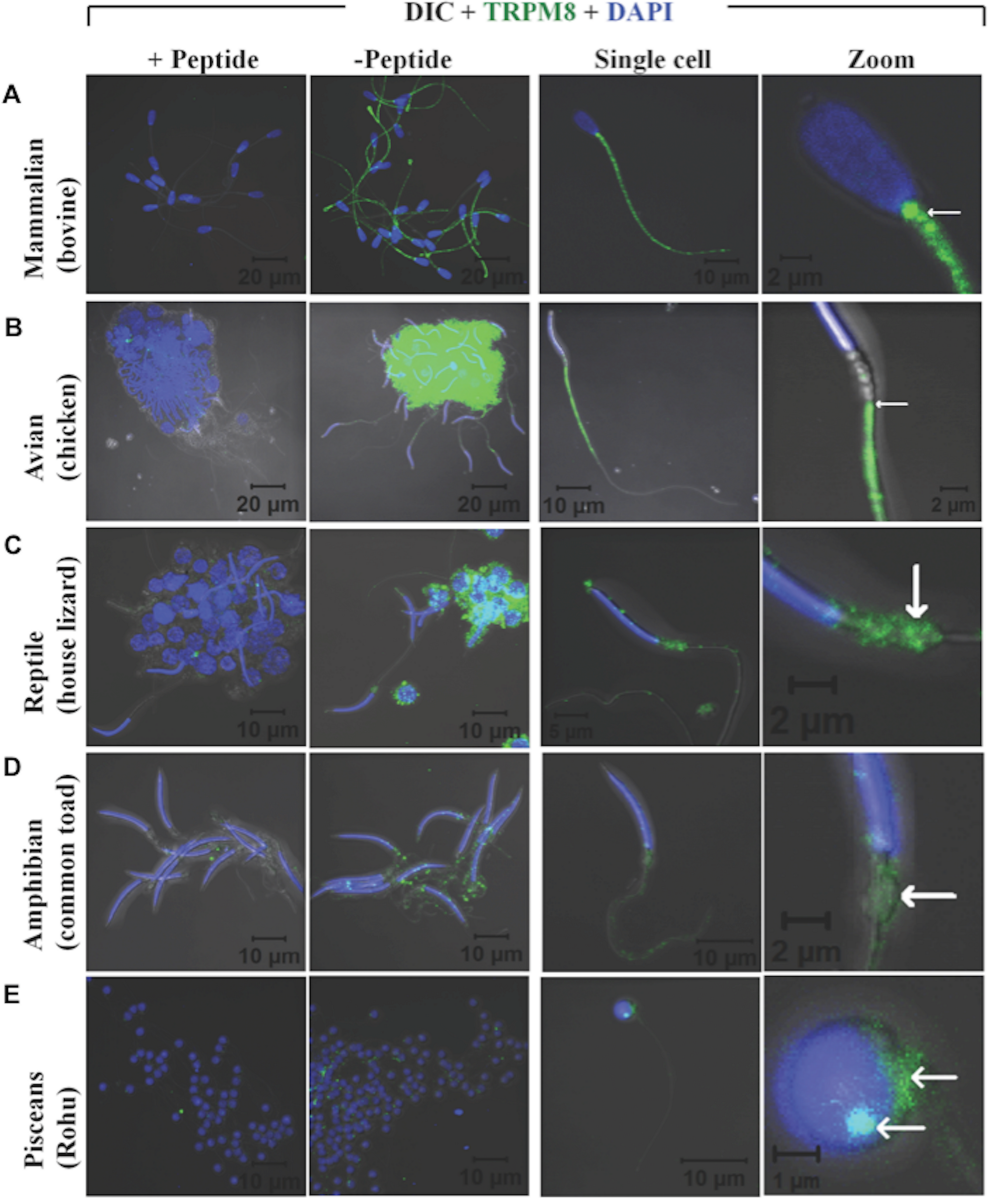
TRPM8 is present in vertebrate sperm cells. Confocal images demonstrating the presence of TRPM8 in mammalian (A, bovine), avian (B, duck), reptilian (C, house lizard), amphibian (D, common toad) and piscean (E, Rohu fish) sperm are shown. Cluster of sperm cells were immunostained for TRPM8-specific antibody in presence or absence of a specific blocking peptide. Fluorescence images representing TRPM8 (green) and DNA (blue) were merged with DIC images. In the right panels, confocal images of TRPM8 expression and localization in single sperm from different species are shown. An enlarged view of the same cell is shown in extreme right. In mammals TRPM8 is exclusively localized in the neck and tail regions. In avian sperm, it is exclusively localized in the tail regions and mostly excluded from head and neck regions. In reptilian and amphibian sperm, TRPM8 is exclusively localized in the neck regions. In piscean, weak yet specific TRPM8 immunoreactivity (restricted in the neck regions) is observed in some but not in all mature sperm cells.

Recently expression and functional involvement of TRPM8 in higher mammals, namely in human and mouse sperm and prostate have been described (De Blas et al., 2009; Martínez-López et al., 2011; Li et al., 2008; Gibbs et al., 2011). Since our *in silico* analysis indicated that TRPM8 is a well conserved channel involved in thermosensation, we hypothesized that TRPM8 might be present in the sperm cells of different species, especially because sperm cells are highly responsive to temperature fluctuations. To explore these aspects, we checked the endogenous expression of TRPM8 in the sperm cells from different species ranging from lower vertebrates to higher mammals by indirect immunofluorescence analysis using specific antibodies. To confirm that the immunoreactivity due to this antibody is really specific, we used zebrafish as a negative control as the TRPM8 gene is absent in zebrafish genome (Kastenhuber et al., 2013). We used testis section of zebrafish and noted that these tissues reveal no staining when probed under the same conditions as mice testis sections, which show prominent TRPM8 immunoreactivity (Fig. 6). Our *in silico* analysis also confirms that the epitope sequence (SDVDGTTYDFAHC) recognized by the TRPM8-specific antibody is highly conserved throughout the vertebrates (Fig. S2A). In addition, the same sequence is absent in other TRPM sequences (Fig. S2B). By using this specific antibody we confirmed that TRPM8 is expressed in the sperm cells in these representative species (ranging from fish, amphibians, reptiles, avian and mammals) (Fig. 7). These TRPM8-specific immunoreactivities were abolished/reduced when we used a specific blocking peptide.

Next, we performed western blot analysis to confirm the expression of TRPM8 in sperm cells. We have detected the expression of TRPM8 in bull and fish sperm confirming the expression of TRPM8 in these cells (data not shown). To prove further that TRPM8 has functional significance in sperm cell, we used bull sperm as a model system and analysed its motility in response to TRPM8 activator and inhibitor. We noted that activation of TRPM8 results in significant drop in sperm motility and inhibition of TRPM8 results in marginal increase in sperm motility (Movie 1: https://youtu.be/0esyio_N5pY). Taken together, the results suggest that the conserved expression of TRPM8 in sperm cells of vertebrate origin correlate well with its level of gene-specific functional constraints that is important throughout the vertebrate evolution.

Overall, our results strongly suggest that TRPM8 has evolved nearly 400 million years ago and is an evolutionary conserved ion channel. We propose that expression of TRPM8 in sperm cell is a major aspect that has guided its molecular evolution.

## Discussion

In this work we have explored the nature of conservation of a thermosensitive ion channel TRPM8 and our findings indicate that the molecular evolution of TRPM8 correlates well with vertebrate evolution.

### TRPM8 in the context of evolutionary conserved physiological functions

So far it has been proposed that TRPM8 is involved in the detection of cold temperature and thus involved in thermosensation, especially in endotherms. Interestingly, it seems that the amphibian genome contains two open reading frames for TRPM8 that are homologous to mammalian TRPM8 (Saito & Shingai, 2006). These two copies in *Xenopus* i.e., xlTRPM8 and xlTRPM8b display 65% and 70% identity to the rat TRPM8 protein sequence, respectively. Out of these two, only the xlTRPM8 is responsive to cold and menthol (Myers, Sigal & Julius, 2009). Moreover, in comparison to the TRPM8 channels of homeothermic animals (i.e., TRPM8 in mammals and birds, which gets activated at low temperatures like 25 °C), their poikilothermic counterparts (such as frogs) require a substantially lower temperatures to become activated. It is important to note that body temperature of amphibians as well as the temperature of their ecological niches are lower than the core body temperature of both mammals and birds (Myers, Sigal & Julius, 2009).

As activation temperature for TRPM8 is different for different species, it is tempting to speculate that these changes in the activation temperature correlate with the different environmental temperatures and ecological niches. This is supported by the fact that the trajectory of TRPM8 evolution reveals different slopes in different era (Fig. 1B). For example, during the radiation of non-human primates, changes are minimum while changes are higher during radiation of mammals (Fig. 1B). In the same manner, TRPM8 underwent more changes during evolution of warm blooded animals from cold blooded animals such as evolution of birds from reptiles which occurred around 150 MYA.

### Involvement of TRP channels in evolution and presence of TRPM8 in fish

We have analyzed the molecular evolution of TRPM8 and the conservation of its different domains and motifs. In addition, we demonstrate that TRPM8 is expressed endogenously in sperm cells from all vertebrates tested so far suggesting that functional importance of TRPM8 remain conserved in all vertebrate sperm cells. Our data shows that TRPM8 is well conserved throughout evolution (*p* value ≤ 0.0001, based on the comparison of TRPM8 protein sequence among total 24 different species, ranging from human to lower vertebrates) (Figs. 1 and 2). Notably, based on the zebrafish genome sequence, it has been described that TRPM8 is absent in the fish lineages but two divergent copies of TRPM8 are present in *Xenopus* (Gibbs et al., 2011). We detected two copies of TRPM8 each in the two well known amphibian genomes as detected by BLAST tool, namely *Xenopus laevis* (NP_001155105.1 and NP_001155106.1) and *X. tropicalis* (NP_001155066.1 and NP_001155067.1). However, further search for two copies of TRPM8 in some other amphibians was constrained due to limited or incomplete amphibian genomic resources. We attempted to run homology scan for TRPM8 against the EST database of the axolotl salamander, *Ambystoma mexicanum* (http://www.ambystoma.org), but we didn’t find any hit for TRPM8 in the current assembly. Based on this observation, previously it has been suggested that TRPM8 might have evolved with these ectotherms. As TRPM8 is present in the Coelacanth (the oldest known living lineage of lobed-finned fish which appeared approximately 400 MYA, Ensembl accession id ENSLACP00000016045), but not present in zebrafish, it needs more detailed studies including different fish species. Unavailability of the complete TRPM8 sequences at any of the query databases, especially for few selected species (such as in other fishes and reptiles) make it difficult to predict the exact evolutionary history and trajectory. However, the presence or absence of TRPM8 in different fish lineages suggest that it had probably evolved during evolution of fishes, but lost in certain lineages subsequently.

In particular, absence of TRPM8 in Zebrafish and presence of TRPM8 in Rohu is intriguing. However, there are several possibilities by which this conflicting situation can be explained. Firstly, it is most likely that ancient fish had TRPM8 and then this gene has been either retained or lost in certain lineages depending on the requirements, i.e., adaptation and speciation driven by molecular evolution. For instance, Zebrafish has a small body and genome size, short life-span and typical reproduction behavior (i.e., it breeds throughout the year) which suggests that this fish has evolved and survived in a “resource-limited environment.” Moreover, as it had its origin in the Himalayan region, which is a relatively cold area therefore, Zebrafish has probably benefitted by losing TRPM8 while some other teleosts have retained it for certain sensory processes and other physiological benefits. Secondly, the reproductive seasonality and temperature requirement is very different for Zebrafish and Rohu. Zebrafish reproduces throughout the year (therefore to some extent its fertilization process could be independent of temperature) and it also reproduces in all laboratory conditions. On the other hand, reproduction of Rohu is highly seasonal and depends on precise temperature and other environmental factors. Thirdly, it is important to note that horizontal gene transfer of TRPM8 is possible in aquatic animals. Such horizontal gene transfer has been documented across the genus and classic examples are the transfer of antifreeze protein and Tc1 element in fish, which provides adaptive benefit to survive in cold regions or against parasites (Graham et al., 2012; Graham et al., 2008; Kuraku, Qiu & Meyer, 2012; Robinson, Sieber & Dunning Hotopp, 2013; Sorhannus, 2012). Therefore, such horizontal transfer of TRPM8 gene to Rohu from other sources cannot be ruled out completely. Nevertheless, using specific antibody and its specific blocking peptide in independent experiments such as immunofluorescence analysis, FACS and Western blot analysis, our results suggest that a subpopulation (not all cells) of Rohu fish sperm cells express TRPM8 at the protein level, at least the specific epitope *per se*.

Taken together, all these results suggest an evolutionary conserved role of TRPM8 in thermosensation as experienced by sperm. In this study we have also demonstrated that true TRPM8 orthologs are missing in invertebrates. There are four TRPM-like genes in the *C. elegans* genome, namely gon-2 (abnormal gonad development), gtl-1 (gon-two like 1), gtl-2 (gon-two like 2), and ced-11 (cell death abnormal) (Okamura et al., 2005; Xiao & Xu, 2009). Additionally, Ciona has also TRPM-like genes (Okamura et al., 2005). This also supports that TRPM-like genes are present in invertebrates but these species lack genuine TRPM8 ortholog. However, further in-depth studies are required in this context, which is beyond scope of the current study.

### TRPM8 in the context of conserved cellular functions

This work along with other previous studies have confirmed the presence of TRPM8 in many vertebrates tested so far, both at the genomic as well as at the protein level. This work also suggests that involvement of TRPM8 is conserved in the context of sperm cells and possibly also in the bone cells. We provide evidence supporting the endogenous expression of TRPM8 in sperm of all representative vertebrates. This is in line with the recent reports demonstrating the expression of TRPM channels in human, mouse sperm cells as well as in rat spermatogenic cells (De Blas et al., 2009; Martínez-López et al., 2011; Li et al., 2008). It is important to mention that recently a protein (Cysteine-rich secretory protein 4) which regulates sperm function has been characterized as an endogenous inhibitor of TRPM8 (Gibbs et al., 2011). All these in general suggest involvement of TRPM8 in critical reproductive functions such as sperm development, sperm movement and fertility in vertebrates and hence contributes to “their reproductive fitness.” Notably, all these factors are dependent on temperature and seasonality.

Detection of TRPM8 in sperm cells of early vertebrates is intriguing. The expression of TRPM8 in sperm cell suggests that TRPM8 may have played an important role in the adaptation (in response to temperature) of warm-blooded (homeothermic animals) and cold-blooded (poikilothermic) animals in different ecological niche, especially in animals (such as in fish and amphibians) where fertilization is exogenous in nature. Though we have detected TRPM8 expression in sperm cells from all the vertebrates that we have tested so far, an interesting pattern of TRPM8 localization is worth mentioning. In our analysis, we noted that the localization of TRPM8 is mainly restricted in the tail region in case of homeothermic animals (such as mammals and avian) with warm blood and having internal fertilization. In contrast, poikilothermic animals with cold blood (such as fish, amphibians and reptiles) the localization of TRPM8 is mainly restricted in the neck region which contains mitochondria. Nevertheless, conserved expression of TRPM8 in the vertebrate sperm cells strongly suggests the evolutionary conserved role of TRPM8 and may also explain the thermosensitivity observed in these motile cells.

We noted that the TRPM8 shares same genomic locus with secreted phosphoprotein 2 (SPP2) throughout the vertebrate evolution. As SPP2 is directly involved in the BMP-signaling and bone development, close association of TRPM8 with SPP2 is intriguing (Brochmann, Behnam & Murray, 2009). Though speculative, yet this close relationship of both TRPM8 and SPP2 for 400 MYA (since vertebrate evolution started), is indicative of their involvement in bone formation and may argue for a possible co-evolution at molecular level. This is in full agreement with reports suggesting the presence of TRPM8 in bone cells (Abed et al., 2009). This association also correlates well with the Ca^2+^-signaling involved in the bone formation (Väänänen et al., 2000). For example, Menthol and its metabolites have been shown to inhibit bone resorption when fed to rats (Mühlbauer , 2003; Spichiger et al., 2006). Similarly, TRPM8 also express in human odontoblasts and co-localize with dentine sialophosphoprotein (DSPP) (El Karim et al., 2011). Although presence of TRPM8 in bone cells has not been established yet, the relationship between TRPM8 and bone formation is intriguing and important in the context of notochord formation, the prime feature of vertebrates. Notably, Zebrafish, which lack TRPM8 in its genome, also lack SPP2, suggesting that these two might be involved in a common function. How TRPM8 and SPP2 share molecular interaction relevant for the bone development is not clear at this point and further studies are needed.

Overall, our studies indicate that expression of TRPM8 in vertebrate sperm could be an important factor in the molecular evolution of TRPM8 which correlates well with vertebrate evolution.

## Supplemental Information

10.7717/peerj.1310/supp-1 Table S1 List of TRPM-like genes from invertebrates

10.7717/peerj.1310/supp-2 Table S2 Summary of missense variants of human TRPM8, deduced from 1000 genome datasets

10.7717/peerj.1310/supp-3 Figure S1 TRPM-like genes are present in invertebrates Invertebrates have TRPM-like genes but not authentic TRPM8 as demonstrated by Maximum-likelihood method based phylogenetic tree. Bootstrap = 1,000.

10.7717/peerj.1310/supp-4 Figure S2 Conservation of the epitope of the TRPM8-specific antibody (A) The epitome sequence of the anti-TRPM8 antibody used in this work is highly conserved in all species for which TRPM8 sequences are available. (B) The TRPM8 epitope sequence is missing in all other TRPM channels.

## Additional Information and Declarations

### Data Availability

The following information was supplied regarding data availability: Accession numbers can be found in Table 1 of this manuscript.
