# Supplementary material for: Expression of temperature-sensitive ion channel TRPM8 in sperm cells correlates with vertebrate evolution
Source: PeerJ. 2015 Oct 13;3:e1310. doi: 10.7717/peerj.1310 (PMC4614861; doi:10.7717/peerj.1310)
Supplement: Table S1 [file peerj-03-1310-s001.docx]

**Supplementary table S1: List of TRPM-like genes from invertebrates**

| **Invertebrates** | **Accession ids in Genbank** | **Gene annotations** | **Seq. Length** | **min. eValue** | **mean Similarity** | **#GOs** | **GOs** | **InterProScan** |
| --- | --- | --- | --- | --- | --- | --- | --- | --- |
| Acyrthosiphon pisum | gi\|328713847\|ref\|XP_001950420.2\| | transient receptor potential cation channel cg34123-like | 1089 | 0 | 81.50% | 10 | F:metal ion transmembrane transporter activity; P:zinc ion homeostasis; P:divalent metal ion transport; P:mitochondrion organization; P:thermotaxis; P:cell growth; P:transmembrane transport; P:magnesium ion homeostasis; C:integral to membrane; F:ion channel activity | IPR005821 (PFAM); PTHR13800 (PANTHER), PTHR13800:SF0 (PANTHER), tmhmm (TMHMM) |
| Aplysia californica | gi\|524875233\|ref\|XP_005094347.1\| | transient receptor potential cation channel subfamily m member 2 isoform x2 | 1478 | 0 | 52.75% | 5 | C:integral to membrane; F:ion channel activity; F:hydrolase activity; P:ion transport; P:transmembrane transport | IPR005821 (PFAM); PTHR13800 (PANTHER), PTHR13800:SF0 (PANTHER), tmhmm (TMHMM) |
| Bombyx mori | gi\|512936452\|ref\|XP_004933523.1\| | transient receptor potential cation channel trpm-like [Bombyx mori] | 1338 | 0 | 82.80% | 10 | F:metal ion transmembrane transporter activity; P:zinc ion homeostasis; P:divalent metal ion transport; P:mitochondrion organization; P:thermotaxis; P:cell growth; P:transmembrane transport; P:magnesium ion homeostasis; C:integral to membrane; F:ion channel activity | IPR005821 (PFAM); G3DSA:1.20.5.1010 (GENE3D), PTHR13800 (PANTHER), PTHR13800:SF0 (PANTHER), tmhmm (TMHMM) |
| Branchiostoma floridae | gi\|260794336\|ref\|XP_002592165.1\| | transient receptor potential cation channel subfamily m member 2 | 1553 | 0 | 60.80% | 9 | F:sodium channel activity; F:calcium channel activity; F:ADP-ribose diphosphatase activity; C:integral to plasma membrane; P:response to hydroperoxide; P:calcium ion transmembrane transport; P:manganese ion transmembrane transport; F:manganese ion transmembrane transporter activity; P:sodium ion transport | IPR000086 (PROFILE); IPR005821 (PFAM); IPR015797 (G3DSA:3.90.79.GENE3D); G3DSA:3.40.50.450 (GENE3D), PTHR13800 (PANTHER), PTHR13800:SF0 (PANTHER) |
| Capitella teleta | gi\|443702340\|gb\|ELU00429.1\| | transient receptor potential cation channel subfamily m member 3 isoform x23 | 1628 | 0 | 56.10% | 4 | C:integral to membrane; F:calcium channel activity; P:cation transport; P:transmembrane transport | IPR005821 (PFAM); IPR015797 (G3DSA:3.90.79.GENE3D); PTHR13800 (PANTHER), PTHR13800:SF0 (PANTHER), tmhmm (TMHMM) |
| Ceratitis capitata | gi\|498937077\|ref\|XP_004520562.1\| | transient receptor potential cation channel trpm-like isoform X2 [Ceratitis capitata] | 1225 | 0 | 95.85% | 10 | F:metal ion transmembrane transporter activity; P:zinc ion homeostasis; P:divalent metal ion transport; P:mitochondrion organization; P:thermotaxis; P:cell growth; P:transmembrane transport; P:magnesium ion homeostasis; C:integral to membrane; F:ion channel activity | IPR005821 (PFAM); PTHR13800 (PANTHER), PTHR13800:SF0 (PANTHER), tmhmm (TMHMM) |
| Crassostrea gigas | gi\|405966757\|gb\|EKC31999.1\| | transient receptor potential cation channel subfamily m member 2 | 1158 | 0 | 60.35% | 9 | F:sodium channel activity; F:calcium channel activity; F:ADP-ribose diphosphatase activity; C:integral to plasma membrane; P:response to hydroperoxide; P:calcium ion transmembrane transport; P:manganese ion transmembrane transport; F:manganese ion transmembrane transporter activity; P:sodium ion transport | IPR005821 (PFAM); PTHR13800 (PANTHER), PTHR13800:SF0 (PANTHER) |
| Danaus plexippus | gi\|357629934\|gb\|EHJ78405.1\| | transient receptor potential cation channel trpm-like [Bombyx mori] | 774 | 0 | 88.45% | 10 | F:metal ion transmembrane transporter activity; P:zinc ion homeostasis; P:divalent metal ion transport; P:mitochondrion organization; P:thermotaxis; P:cell growth; P:transmembrane transport; P:magnesium ion homeostasis; C:integral to membrane; F:ion channel activity | PTHR13800 (PANTHER), PTHR13800:SF0 (PANTHER) |
| Hydra magnipapillata | gi\|449668329\|ref\|XP_002168052.2\| | transient receptor potential cation channel subfamily m member 2-like | 1053 | 0 | 53.85% | 8 | F:sodium channel activity; F:calcium channel activity; F:ADP-ribose diphosphatase activity; C:integral to plasma membrane; P:calcium ion transport; P:transmembrane transport; P:response to hydroperoxide; F:manganese ion transmembrane transporter activity | IPR005821 (PFAM); G3DSA:3.40.50.450 (GENE3D), PTHR13800 (PANTHER), PTHR13800:SF0 (PANTHER), tmhmm (TMHMM) |
| Strongylocentrotus purpuratus | gi\|390350651\|ref\|XP_792292.3\| | transient receptor potential cation subfamily member 3 | 1468 | 0 | 57.50% | 4 | C:integral to membrane; F:cation channel activity; P:ion transmembrane transport; P:cation transport | IPR005821 (PFAM); PTHR13800 (PANTHER), PTHR13800:SF0 (PANTHER), tmhmm (TMHMM), SSF81324 (SUPERFAMILY) |
| Trichoplax adhaerens | gi\|196009504\|ref\|XP_002114617.1\| | transient receptor potential cation channel subfamily m member 3 | 855 | 0 | 55.55% | 5 | F:calcium channel activity; P:ion transmembrane transport; F:hydrolase activity; P:cation transport; C:integral to membrane | PTHR13800 (PANTHER), PTHR13800:SF0 (PANTHER) |
