# Supplementary material for: Expression of temperature-sensitive ion channel TRPM8 in sperm cells correlates with vertebrate evolution
Source: PeerJ. 2015 Oct 13;3:e1310. doi: 10.7717/peerj.1310 (PMC4614861; doi:10.7717/peerj.1310)
Supplement: Table S2 [file peerj-03-1310-s002.pdf]

Missense position	Amino acid change	Variant ID	chromosomal location	Alleles	gmaf	class	Source	status	snptype	sift	polyphen	Transcript
2	S/C	rs200895419	2:2348351872:234835187	C/G	0	SNP	dbSNP	0	Missense variant	111tolerated (0.11)	68benign (0.067)	ENST00000324695
2	S/C	rs200895419	2:2348351872:234835187	C/G	0	SNP	dbSNP	0	NMD transcript variant, Missense variant	91tolerated(0.09)	411benign(0.41)	ENST00000444298
2	E/G	rs201054729	2:2348460412:234846041	A/G	0	SNP	dbSNP	0	Missense variant	11deleterious(0.01)	25benign(0.024)	ENST00000409625
3	S/F	TMP_ESP_2_234845963	2:2348459632:234845963	C/T	0	SNP	ESP	ESP,	Missense variant	1deleterious(0)	771possibly damaging(0.77)	ENST00000355722
3	G/A	rs113094804	2:2348460442:234846044	G/C	0	SNP	dbSNP	Frequency,	Missense variant	161tolerated(0.16)	82benign(0.081)	ENST00000409625
3	V/A	rs201483334	2:2348904592:234890459	T/C	0	SNP	dbSNP	0	NMD transcript variant, Missense variant	101tolerated(0.1)	133benign(0.132)	ENST00000439148
4	R/W	rs201572946	2:2348351922:234835192	C/T	0	SNP	dbSNP	0	Missense variant	11deleterious(0.01)	7benign(0.006)	ENST00000324695
4	R/W	rs201572946	2:2348351922:234835192	C/T	0	SNP	dbSNP	0	NMD transcript variant, Missense variant	1deleterious(0)	301benign(0.3)	ENST00000444298
4	T/I	rs199594552	2:2348460472:234846047	C/T	0	SNP	dbSNP	0	Missense variant	141tolerated(0.14)	8benign(0.007)	ENST00000409625
7	D/N	TMP_ESP_2_234875280	2:2348752802:234875280	G/A	0	SNP	ESP	ESP,	Missense variant	181tolerated(0.18)	1unknown(0)	ENST00000456930
8	L/F	rs200999555	2:2348352042:234835204	C/T	0	SNP	dbSNP	0	Missense variant	121tolerated(0.12)	1benign(0)	ENST00000324695
8	L/F	rs200999555	2:2348352042:234835204	C/T	0	SNP	dbSNP	0	NMD transcript variant, Missense variant	181tolerated(0.18)	4benign(0.003)	ENST00000444298
10	L/S	TMP_ESP_2_234875290	2:2348752902:234875290	T/C	0	SNP	ESP	ESP,	Missense variant	51tolerated(0.05)	1unknown(0)	ENST00000456930
11	V/M	TMP_ESP_2_234845986	2:2348459862:234845986	G/A	0	SNP	ESP	ESP,	Missense variant	71tolerated(0.07)	87benign(0.086)	ENST00000355722
13	R/I	COSM366374	2:2348352202:234835220	G/T	0	somatic_SNV	COSMIC	0	Missense variant	1deleterious(0)	430benign(0.429)	ENST00000324695
13	R/I	COSM366374	2:2348352202:234835220	G/T	0	somatic_SNV	COSMIC	0	NMD transcript variant, Missense variant	31deleterious(0.03)	843possibly damaging(0.842)	ENST00000444298
14	R/K	COSM165187	2:2348459962:234845996	G/A	0	somatic_SNV	COSMIC	0	Missense variant	351tolerated(0.35)	3benign(0.002)	ENST00000355722
17	V/M	COSM1318964	2:2348460042:234846004	G/A	0	somatic_SNV	COSMIC	0	Missense variant	111tolerated(0.11)	156benign(0.155)	ENST00000355722
17	H/R	rs200767976	2:2348460862:234846086	A/G	0	SNP	dbSNP	0	Missense variant	1deleterious(0)	890possibly damaging(0.889)	ENST00000409625
17	Y/C	COSM242076	2:2348753112:234875311	A/G	0	somatic_SNV	COSMIC	0	Missense variant	11deleterious(0.01)	1unknown(0)	ENST00000456930
18	S/F	rs200718215	2:2348753142:234875314	C/T	0.001 (T)	SNP	dbSNP	0	Missense variant	91tolerated(0.09)	1unknown(0)	ENST00000456930
19	C/Y	rs142251315	2:2348753172:234875317	G/A	0	SNP	dbSNP	Frequency,ESP,	Missense variant	11deleterious(0.01)	1unknown(0)	ENST00000456930
20	E/Q	rs201373026	2:2348460942:234846094	G/C	0.000 (C)	SNP	dbSNP	0	Missense variant	311tolerated(0.31)	94benign(0.093)	ENST00000409625

20	S/L	rs201141727	2:2348586452:234858645	C/T	0	SNP	dbSNP	0	Missense variant	1deleterious(0)	1unknown(0)	ENST00000433712
22	R/W	rs202194673	2:2348352462:234835246	C/T	0	SNP	dbSNP	0	Missense variant	11deleterious(0.01)	226benign(0.225)	ENST00000324695
22	R/W	COSM442528	2:2348352462:234835246	C/T	0	somatic_SNV	COSMIC	0	Missense variant	11deleterious(0.01)	226benign(0.225)	ENST00000324695
22	R/W	rs202194673	2:2348352462:234835246	C/T	0	SNP	dbSNP	0	NMD transcript variant, Missense variant	11deleterious(0.01)	717possibly damaging(0.716)	ENST00000444298
22	R/W	COSM442528	2:2348352462:234835246	C/T	0	somatic_SNV	COSMIC	0	NMD transcript variant, Missense variant	11deleterious(0.01)	717possibly damaging(0.716)	ENST00000444298
22	R/Q	rs200473098	2:2348352472:234835247	G/A	0	SNP	dbSNP	0	Missense variant	431tolerated(0.43)	2benign(0.001)	ENST00000324695
22	R/Q	COSM1406468	2:2348352472:234835247	G/A	0	somatic_SNV	COSMIC	0	Missense variant	431tolerated(0.43)	2benign(0.001)	ENST00000324695
22	R/L	COSM318182	2:2348352472:234835247	G/T	0	somatic_SNV	COSMIC	0	Missense variant	151tolerated(0.15)	21benign(0.02)	ENST00000324695
22	R/Q	rs200473098	2:2348352472:234835247	G/A	0	SNP	dbSNP	0	NMD transcript variant, Missense variant	361tolerated(0.36)	18benign(0.017)	ENST00000444298
22	R/Q	COSM1406468	2:2348352472:234835247	G/A	0	somatic_SNV	COSMIC	0	NMD transcript variant, Missense variant	361tolerated(0.36)	18benign(0.017)	ENST00000444298
22	R/L	COSM318182	2:2348352472:234835247	G/T	0	somatic_SNV	COSMIC	0	NMD transcript variant, Missense variant	151tolerated(0.15)	222benign(0.221)	ENST00000444298
23	T/P	rs201333720	2:2348352492:234835249	A/C	0	SNP	dbSNP	ESP,	Missense variant	281tolerated(0.28)	39benign(0.038)	ENST00000324695
23	T/P	rs201333720	2:2348352492:234835249	A/C	0	SNP	dbSNP	ESP,	NMD transcript variant, Missense variant	291tolerated(0.29)	373benign(0.372)	ENST00000444298
23	S/I	rs148696315	2:2348905192:234890519	G/T	0.001 (T)	SNP	dbSNP	1000Genomes,	NMD transcript variant, Missense variant	1deleterious(0)	982probably damaging(0.981)	ENST00000439148
24	D/N	rs149029668	2:2348461062:234846106	G/A	0	SNP	dbSNP	Frequency,ESP,	Missense variant	201tolerated(0.2)	446possibly damaging(0.445)	ENST00000409625
24	D/N	COSM177742	2:2348461062:234846106	G/A	0	somatic_SNV	COSMIC	0	Missense variant	201tolerated(0.2)	446possibly damaging(0.445)	ENST00000409625
24	D/E	COSM335617	2:2348461082:234846108	C/A	0	somatic_SNV	COSMIC	0	Missense variant	1deleterious(0)	981probably damaging(0.98)	ENST00000409625
24	A/T	rs200708894	2:2348586562:234858656	G/A	0	SNP	dbSNP	0	Missense variant	1deleterious(0)	1unknown(0)	ENST00000433712
25	A/T	rs200565245	2:2348461092:234846109	G/A	0	SNP	dbSNP	0	Missense variant	111tolerated(0.11)	913probably damaging(0.912)	ENST00000409625
27	S/R	COSM292108	2:2348352632:234835263	C/A	0	somatic_SNV	COSMIC	0	Missense variant	61tolerated(0.06)	992probably damaging(0.991)	ENST00000324695
27	S/R	COSM292108	2:2348352632:234835263	C/A	0	somatic_SNV	COSMIC	0	NMD transcript variant, Missense variant	31deleterious(0.03)	1000probably damaging(0.999)	ENST00000444298
28	A/T	rs199755852	2:2348352642:234835264	G/A	0	SNP	dbSNP	ESP,	Missense variant	421tolerated(0.42)	9benign(0.008)	ENST00000324695
28	A/T	rs199755852	2:2348352642:234835264	G/A	0	SNP	dbSNP	ESP,	NMD transcript variant, Missense variant	441tolerated(0.44)	27benign(0.026)	ENST00000444298

28	A/V	rs200600537	2:2348352652:234835265	C/T	0	SNP	dbSNP	0	Missense variant	531tolerated(0.53)	1benign(0)	ENST00000324695
28	A/V	rs200600537	2:2348352652:234835265	C/T	0	SNP	dbSNP	0	NMD transcript variant, Missense variant	531tolerated(0.53)	2benign(0.001)	ENST00000444298
28	P/L	rs199828215	2:2348905342:234890534	C/T	0	SNP	dbSNP	ESP,	NMD transcript variant, Missense variant	1deleterious(0)	1001probably damaging(1)	ENST00000439148
29	E/G	rs201054729	2:2348460412:234846041	A/G	0	SNP	dbSNP	0	Missense variant	41deleterious(0.04)	25benign(0.024)	ENST00000355722
30	R/W	rs199600946	2:2348352702:234835270	C/T	0	SNP	dbSNP	0	Missense variant	21deleterious(0.02)	865possibly damaging(0.864)	ENST00000324695
30	R/W	rs199600946	2:2348352702:234835270	C/T	0	SNP	dbSNP	0	NMD transcript variant, Missense variant	21deleterious(0.02)	957probably damaging(0.956)	ENST00000444298
30	R/Q	rs200774365	2:2348352712:234835271	G/A	0	SNP	dbSNP	ESP,	Missense variant	361tolerated(0.36)	664possibly damaging(0.663)	ENST00000324695
30	R/Q	rs200774365	2:2348352712:234835271	G/A	0	SNP	dbSNP	ESP,	NMD transcript variant, Missense variant	351tolerated(0.35)	871possibly damaging(0.87)	ENST00000444298
30	G/A	rs113094804	2:2348460442:234846044	G/C	0	SNP	dbSNP	Frequency,	Missense variant	501tolerated(0.5)	82benign(0.081)	ENST00000355722
31	T/I	rs199594552	2:2348460472:234846047	C/T	0	SNP	dbSNP	0	Missense variant	251tolerated(0.25)	8benign(0.007)	ENST00000355722
31	A/V	TMP_ESP_2_234875353	2:2348753532:234875353	C/T	0	SNP	ESP	ESP,	Missense variant	1001tolerated(1)	9benign(0.008)	ENST00000456930
33	V/M	rs200856718	2:2348586832:234858683	G/A	0	SNP	dbSNP	0	Missense variant	101tolerated(0.1)	1unknown(0)	ENST00000433712
34	Q/L	rs111316052	2:2348905522:234890552	A/T	0	SNP	dbSNP	Frequency,	NMD transcript variant, Missense variant	31deleterious(0.03)	603possibly damaging(0.602)	ENST00000439148
35	T/A	rs34709945	2:2348753642:234875364	A/G	0	SNP	dbSNP	Frequency,	Missense variant	471tolerated(0.47)	2benign(0.001)	ENST00000456930
36	A/T	rs201855344	2:2348586922:234858692	G/A	0	SNP	dbSNP	0	Missense variant	571tolerated(0.57)	1unknown(0)	ENST00000433712
36	D/Y	COSM383304	2:2348753672:234875367	G/T	0	somatic_SNV	COSMIC	0	Missense variant	91tolerated(0.09)	9benign(0.008)	ENST00000456930
36	M/I	COSM371853	2:2348905592:234890559	G/A	0	somatic_SNV	COSMIC	0	NMD transcript variant, Splice region variant, Missense variant	1deleterious(0)	1000probably damaging(0.999)	ENST00000439148
37	Q/H	COSM342435	2:2348753722:234875372	G/T	0	somatic_SNV	COSMIC	0	Missense variant	31deleterious(0.03)	121benign(0.12)	ENST00000456930
41	A/T	rs141040008	2:2348753822:234875382	G/T/A	0	SNP	dbSNP	Multiple_observations,Frequency,ESP,	Missense variant	761tolerated(0.76)	4benign(0.003)	ENST00000456930
41	A/S	rs141040008	2:2348753822:234875382	G/T/A	0	SNP	dbSNP	Multiple_observations,Frequency,ESP,	Missense variant	1001tolerated(1)	2benign(0.001)	ENST00000456930
44	H/R	rs200767976	2:2348460862:234846086	A/G	0	SNP	dbSNP	0	Missense variant	1deleterious(0)	890possibly damaging(0.889)	ENST00000355722
44	E/Q	rs145069600	2:2348587162:234858716	G/C	0	SNP	dbSNP	Multiple_observations,Frequency,ESP,	Missense variant	701tolerated(0.7)	1unknown(0)	ENST00000433712

47	A/E	COSM109004	2:2348393352:234839335	C/A	0	somatic_SNV	COSMIC	0	Missense variant	1001tolerated(1)	1benign(0)	ENST00000324695
47	A/E	rs144401958	2:2348393352:234839335	C/T/A	0	somatic_SNV	dbSNP	Multiple_observations,Cited,	Missense variant	1001tolerated(1)	1benign(0)	ENST00000324695
47	A/V	rs144401958	2:2348393352:234839335	C/T/A	0	somatic_SNV	dbSNP	Multiple_observations,Cited,	Missense variant	1deleterious(0)	12benign(0.011)	ENST00000324695
47	A/E	COSM109004	2:2348393352:234839335	C/A	0	somatic_SNV	COSMIC	0	NMD transcript variant, Missense variant	1001tolerated(1)	3benign(0.002)	ENST00000444298
47	A/E	rs144401958	2:2348393352:234839335	C/T/A	0	somatic_SNV	dbSNP	Multiple_observations,Cited,	NMD transcript variant, Missense variant	1001tolerated(1)	3benign(0.002)	ENST00000444298
47	A/V	rs144401958	2:2348393352:234839335	C/T/A	0	somatic_SNV	dbSNP	Multiple_observations,Cited,	NMD transcript variant, Missense variant	21deleterious(0.02)	63benign(0.062)	ENST00000444298
47	E/Q	rs201373026	2:2348460942:234846094	G/C	0.000 (C)	SNP	dbSNP	0	Missense variant	281tolerated(0.28)	94benign(0.093)	ENST00000355722
48	R/C	rs145944024	2:2348587282:234858728	C/T	0.001 (T)	SNP	dbSNP	Multiple_observations,Frequency,1000Genomes,ESP,	Missense variant	51tolerated(0.05)	1unknown(0)	ENST00000433712
48	R/H	rs201962335	2:2348587292:234858729	G/A	0	SNP	dbSNP	ESP,	Missense variant	161tolerated(0.16)	1unknown(0)	ENST00000433712
49	V/I	rs200414800	2:2348888382:234888838	G/A	0.001 (A)	SNP	dbSNP	ESP,	Missense variant	351tolerated(0.35)	10benign(0.009)	ENST00000456930
50	E/D	rs138133683	2:2348476742:234847674	A/C	0	SNP	dbSNP	Frequency,ESP,	Missense variant	661tolerated(0.66)	41benign(0.04)	ENST00000409625
50	W/R	TMP_ESP_2_234891736	2:2348917362:234891736	T/C	0	SNP	ESP	ESP,	NMD transcript variant, Missense variant	31deleterious(0.03)	999probably damaging(0.998)	ENST00000439148
51	K/T	rs200892764	2:2348393472:234839347	A/C	0	SNP	dbSNP	0	Missense variant	1deleterious(0)	995probably damaging(0.994)	ENST00000324695
51	K/T	rs200892764	2:2348393472:234839347	A/C	0	SNP	dbSNP	0	NMD transcript variant, Missense variant	11deleterious(0.01)	998probably damaging(0.997)	ENST00000444298
51	D/N	rs149029668	2:2348461062:234846106	G/A	0	SNP	dbSNP	Frequency,ESP,	Missense variant	161tolerated(0.16)	446possibly damaging(0.445)	ENST00000355722
51	D/N	COSM177742	2:2348461062:234846106	G/A	0	somatic_SNV	COSMIC	0	Missense variant	161tolerated(0.16)	446possibly damaging(0.445)	ENST00000355722
51	D/E	COSM335617	2:2348461082:234846108	C/A	0	somatic_SNV	COSMIC	0	Missense variant	1deleterious(0)	981probably damaging(0.98)	ENST00000355722
51	P/T	rs145572343	2:2348587372:234858737	C/A	0	SNP	dbSNP	Multiple_observations,Frequency,	Missense variant	171tolerated(0.17)	1unknown(0)	ENST00000433712
52	R/Q	TMP_ESP_2_234839350	2:2348393502:234839350	G/A	0	SNP	ESP	ESP,	Missense variant	11deleterious(0.01)	280benign(0.279)	ENST00000324695
52	R/Q	TMP_ESP_2_234839350	2:2348393502:234839350	G/A	0	SNP	ESP	ESP,	NMD transcript variant, Missense variant	11deleterious(0.01)	750possibly damaging(0.749)	ENST00000444298
52	A/T	rs200565245	2:2348461092:234846109	G/A	0	SNP	dbSNP	0	Missense variant	91tolerated(0.09)	913probably damaging(0.912)	ENST00000355722
52	R/C	TMP_ESP_2_234858740	2:2348587402:234858740	C/T	0	SNP	ESP	ESP,	Missense variant	31deleterious(0.03)	1unknown(0)	ENST00000433712

52	R/C	COSM210452	2:2348587402:234858740	C/T	0	somatic_SNV	COSMIC	0	Missense variant	31deleterious(0.03)	1unknown(0)	ENST00000433712
52	R/H	rs145044224	2:2348587412:234858741	G/A	0.002 (A)	SNP	dbSNP	Multiple_observations,Frequency,1000 Genomes,ESP,	Missense variant	91tolerated(0.09)	1unknown(0)	ENST00000433712
52	V/A	rs200135938	2:2348888482:234888848	T/C	0	SNP	dbSNP	0	Missense variant	911tolerated(0.91)	3benign(0.002)	ENST00000456930
52	V/A	rs201253609	2:2348917432:234891743	T/C	0	SNP	dbSNP	0	NMD transcript variant, Missense variant	31deleterious(0.03)	619possibly damaging(0.618)	ENST00000439148
53	T/M	rs202102033	2:2348587442:234858744	C/T	0	SNP	dbSNP	0	Missense variant	431tolerated(0.43)	1unknown(0)	ENST00000433712
53	N/S	rs200970660	2:2348888512:234888851	A/G	0	SNP	dbSNP	0	Missense variant	731tolerated(0.73)	12benign(0.011)	ENST00000456930
55	S/F	rs201051067	2:2348587502:234858750	C/T	0	SNP	dbSNP	0	Missense variant	11deleterious(0.01)	1unknown(0)	ENST00000433712
55	S/F	COSM138349	2:2348587502:234858750	C/T	0	somatic_SNV	COSMIC	0	Missense variant	11deleterious(0.01)	1unknown(0)	ENST00000433712
56	R/W	rs201761766	2:2348587522:234858752	C/T	0	SNP	dbSNP	0	Missense variant	141tolerated(0.14)	1unknown(0)	ENST00000433712
56	R/Q	rs200303251	2:2348587532:234858753	G/A	0	SNP	dbSNP	0	Missense variant	681tolerated(0.68)	1unknown(0)	ENST00000433712
59	E/Q	COSM1482912	2:2348587612:234858761	G/C	0	somatic_SNV	COSMIC	0	Missense variant	101tolerated(0.1)	1unknown(0)	ENST00000433712
61	S/Y	rs200448470	2:2348393772:234839377	C/A	0	SNP	dbSNP	0	Missense variant	11deleterious(0.01)	185benign(0.184)	ENST00000324695
61	S/Y	rs200448470	2:2348393772:234839377	C/A	0	SNP	dbSNP	0	NMD transcript variant, Missense variant	11deleterious(0.01)	418benign(0.417)	ENST00000444298
62	T/N	rs140502895	2:2348587712:234858771	C/T/A	0.001 (T)	SNP	dbSNP	Multiple_observations,Frequency,ESP,	Missense variant	1deleterious(0)	1unknown(0)	ENST00000433712
62	T/I	rs140502895	2:2348587712:234858771	C/T/A	0.001 (T)	SNP	dbSNP	Multiple_observations,Frequency,ESP,	Missense variant	1001tolerated(1)	1unknown(0)	ENST00000433712
63	A/V	rs201742495	2:2348393832:234839383	C/T	0	SNP	dbSNP	0	Missense variant	211tolerated(0.21)	42benign(0.041)	ENST00000324695
63	A/V	rs201742495	2:2348393832:234839383	C/T	0	SNP	dbSNP	0	NMD transcript variant, Missense variant	291tolerated(0.29)	262benign(0.261)	ENST00000444298
64	T/M	rs146149942	2:2348393862:234839386	C/G/T	0	SNP	dbSNP	Multiple_observations,Frequency,ESP,	Splice region variant, Missense variant	161tolerated(0.16)	2benign(0.001)	ENST00000324695
64	T/R	rs146149942	2:2348393862:234839386	C/G/T	0	SNP	dbSNP	Multiple_observations,Frequency,ESP,	Splice region variant, Missense variant	601tolerated(0.6)	3benign(0.002)	ENST00000324695
64	T/M	rs146149942	2:2348393862:234839386	C/G/T	0	SNP	dbSNP	Multiple_observations,Frequency,ESP,	NMD transcript variant, Splice region variant, Missense variant	121tolerated(0.12)	5benign(0.004)	ENST00000444298
64	T/R	rs146149942	2:2348393862:234839386	C/G/T	0	SNP	dbSNP	Multiple_observations,Frequency,ESP,	NMD transcript variant, Splice region variant, Missense variant	541tolerated(0.54)	2benign(0.001)	ENST00000444298

64	S/R	rs199509635	2:2348587782:234858778	T/A	0	SNP	dbSNP	0	Missense variant	101tolerated(0.1)	1unknown(0)	ENST00000433712
64	T/M	rs200017552	2:2348888842:234888884	C/T	0	SNP	dbSNP	0	Missense variant	591tolerated(0.59)	794possibly damaging(0.793)	ENST00000456930
67	V/M	COSM1318964	2:2348460042:234846004	G/A	0	somatic_SNV	COSMIC	0	Missense variant	141tolerated(0.14)	5benign(0.004)	ENST00000324695
67	V/M	COSM1318964	2:2348460042:234846004	G/A	0	somatic_SNV	COSMIC	0	NMD transcript variant, Missense variant	111tolerated(0.11)	34benign(0.033)	ENST00000444298
67	L/P	rs201249162	2:2348477242:234847724	T/C	0	SNP	dbSNP	0	Missense variant	1deleterious(0)	998probably damaging(0.997)	ENST00000409625
68	R/H	TMP_ESP_2_234891791	2:2348917912:234891791	G/A	0	SNP	ESP	ESP,	NMD transcript variant, Missense variant	101tolerated(0.1)	616possibly damaging(0.615)	ENST00000439148
72	A/T	rs201621223	2:2348889072:234888907	G/A	0	SNP	dbSNP	0	Missense variant	201tolerated(0.2)	829possibly damaging(0.828)	ENST00000456930
73	G/R	rs199497591	2:2348477412:234847741	G/C	0	SNP	dbSNP	0	Missense variant	1deleterious(0)	994probably damaging(0.993)	ENST00000409625
74	R/C	rs201721376	2:2348918082:234891808	C/T	0	SNP	dbSNP	0	NMD transcript variant, Missense variant	1deleterious(0)	1000probably damaging(0.999)	ENST00000439148
74	R/H	rs199784199	2:2348918092:234891809	G/A	0	SNP	dbSNP	0	NMD transcript variant, Missense variant	11deleterious(0.01)	860possibly damaging(0.859)	ENST00000439148
75	A/T	COSM217237	2:2348477472:234847747	G/A	0	somatic_SNV	COSMIC	0	Missense variant	371tolerated(0.37)	762possibly damaging(0.761)	ENST00000409625
75	C/R	rs145663793	2:2348625792:234862579	T/C	0	SNP	dbSNP	Multiple_observations,Frequency,ESP,	Missense variant	1001tolerated(1)	1unknown(0)	ENST00000433712
75	S/L	COSM171795	2:2348918122:234891812	C/T	0	somatic_SNV	COSMIC	0	NMD transcript variant, Missense variant	11deleterious(0.01)	618possibly damaging(0.617)	ENST00000439148
76	K/E	rs150424704	2:2348477502:234847750	A/G	0	SNP	dbSNP	ESP,	Missense variant	221tolerated(0.22)	335benign(0.334)	ENST00000409625
76	F/I	rs200252660	2:2348889192:234888919	T/A	0	SNP	dbSNP	0	Missense variant	91tolerated(0.09)	429benign(0.428)	ENST00000456930
77	E/D	rs138133683	2:2348476742:234847674	A/C	0	SNP	dbSNP	Frequency,ESP,	Missense variant	651tolerated(0.65)	41benign(0.04)	ENST00000355722
77	R/W	rs151304365	2:2348889222:234888922	C/T/A	0	SNP	dbSNP	ESP,	Splice region variant, Missense variant	1deleterious(0)	1000probably damaging(0.999)	ENST00000456930
77	R/W	COSM1018198	2:2348889222:234888922	C/T	0	somatic_SNV	COSMIC	0	Splice region variant, Missense variant	1deleterious(0)	1000probably damaging(0.999)	ENST00000456930
79	E/G	rs201054729	2:2348460412:234846041	A/G	0	SNP	dbSNP	0	Missense variant	11deleterious(0.01)	961probably damaging(0.96)	ENST00000324695
79	E/G	rs201054729	2:2348460412:234846041	A/G	0	SNP	dbSNP	0	NMD transcript variant, Missense variant	11deleterious(0.01)	998probably damaging(0.997)	ENST00000444298
80	G/A	rs113094804	2:2348460442:234846044	G/C	0	SNP	dbSNP	Frequency,	Missense variant	721tolerated(0.72)	727possibly damaging(0.726)	ENST00000324695
80	G/A	rs113094804	2:2348460442:234846044	G/C	0	SNP	dbSNP	Frequency,	NMD transcript variant, Missense variant	711tolerated(0.71)	999probably damaging(0.998)	ENST00000444298
81	T/I	rs199594552	2:2348460472:234846047	C/T	0	SNP	dbSNP	0	Missense variant	401tolerated(0.4)	4benign(0.003)	ENST00000324695

81	T/I	rs199594552	2:2348460472:234846047	C/T	0	SNP	dbSNP	0	NMD transcript variant, Missense variant	321tolerated(0.32)	28benign(0.027)	ENST00000444298
81	K/T	rs199716904	2:2348477662:234847766	A/C	0	SNP	dbSNP	ESP,	Missense variant	91tolerated(0.09)	378benign(0.377)	ENST00000409625
82	N/S	rs28902201	2:2348904322:234890432	A/G	0.005 (G)	SNP	dbSNP	Multiple_observations,Frequency,HapMap,1000Genomes,ESP,	Missense variant	821tolerated(0.82)	15benign(0.014)	ENST00000456930
84	M/R	rs200821010	2:2348477752:234847775	T/G	0	SNP	dbSNP	0	Missense variant	1deleterious(0)	76benign(0.075)	ENST00000409625
86	K/N	COSM264218	2:2348477822:234847782	G/T	0	somatic_SNV	COSMIC	0	Missense variant	201tolerated(0.2)	82benign(0.081)	ENST00000409625
87	A/I	COSM97113	2:2348626152:234862615-234862616	GC/AT	0	somatic_substitution	COSMIC	0	Missense variant	11deleterious(0.01)	1unknown(0)	ENST00000433712
88	F/S	rs201611229	2:2348477872:234847787	T/C	0	SNP	dbSNP	0	Missense variant	1deleterious(0)	924probably damaging(0.923)	ENST00000409625
88	V/M	rs149328116	2:2348918502:234891850	G/A	0.001 (A)	SNP	dbSNP	Multiple_observations,Frequency,ESP,	NMD transcript variant, Missense variant	11deleterious(0.01)	247benign(0.246)	ENST00000439148
89	S/N	rs190304256	2:2348477902:234847790	G/A	0.001 (A)	SNP	dbSNP	0	Missense variant	371tolerated(0.37)	115benign(0.114)	ENST00000409625
89	S/R	rs199553680	2:2348477912:234847791	C/T/A	0.001 (T)	SNP	dbSNP	ESP,	Missense variant	1001tolerated(1)	4benign(0.003)	ENST00000409625
90	R/W	rs138240187	2:2348477922:234847792	C/T	0	SNP	dbSNP	Frequency,ESP,	Missense variant	1deleterious(0)	989probably damaging(0.988)	ENST00000409625
91	V/A	rs201483334	2:2348904592:234890459	T/C	0	SNP	dbSNP	0	Missense variant	281tolerated(0.28)	978probably damaging(0.977)	ENST00000456930
92	V/M	rs199993016	2:2348918622:234891862	G/A	0	SNP	dbSNP	0	NMD transcript variant, Missense variant	141tolerated(0.14)	871possibly damaging(0.87)	ENST00000439148
92	V/M	COSM210455	2:2348918622:234891862	G/A	0	somatic_SNV	COSMIC	0	NMD transcript variant, Missense variant	141tolerated(0.14)	871possibly damaging(0.87)	ENST00000439148
94	H/R	rs200767976	2:2348460862:234846086	A/G	0	SNP	dbSNP	0	Missense variant	1deleterious(0)	358benign(0.357)	ENST00000324695
94	H/R	rs200767976	2:2348460862:234846086	A/G	0	SNP	dbSNP	0	NMD transcript variant, Missense variant	1deleterious(0)	418benign(0.417)	ENST00000444298
94	L/P	rs201249162	2:2348477242:234847724	T/C	0	SNP	dbSNP	0	Missense variant	1deleterious(0)	998probably damaging(0.997)	ENST00000355722
94	G/S	rs201072281	2:2348918682:234891868	G/A	0	SNP	dbSNP	0	NMD transcript variant, Missense variant	31deleterious(0.03)	725possibly damaging(0.724)	ENST00000439148
97	E/Q	rs201373026	2:2348460942:234846094	G/C	0.000 (C)	SNP	dbSNP	0	Missense variant	81tolerated(0.08)	713possibly damaging(0.712)	ENST00000324695
97	E/Q	rs201373026	2:2348460942:234846094	G/C	0.000 (C)	SNP	dbSNP	0	NMD transcript variant, Missense variant	81tolerated(0.08)	888possibly damaging(0.887)	ENST00000444298
97	S/Y	COSM336694	2:2348478142:234847814	C/A	0	somatic_SNV	COSMIC	0	Missense variant	1deleterious(0)	337benign(0.336)	ENST00000409625
100	G/R	rs199497591	2:2348477412:234847741	G/C	0	SNP	dbSNP	0	Missense variant	1deleterious(0)	994probably damaging(0.993)	ENST00000355722

101	D/N	rs149029668	2:2348461062:234846106	G/A	0	SNP	dbSNP	Frequency,ESP,	Missense variant	61tolerated(0.06)	997probably damaging(0.996)	ENST00000324695
101	D/N	COSM177742	2:2348461062:234846106	G/A	0	somatic_SNV	COSMIC	0	Missense variant	61tolerated(0.06)	997probably damaging(0.996)	ENST00000324695
101	D/N	rs149029668	2:2348461062:234846106	G/A	0	SNP	dbSNP	Frequency,ESP,	NMD transcript variant, Missense variant	201tolerated(0.2)	999probably damaging(0.998)	ENST00000444298
101	D/N	COSM177742	2:2348461062:234846106	G/A	0	somatic_SNV	COSMIC	0	NMD transcript variant, Missense variant	201tolerated(0.2)	999probably damaging(0.998	ENST00000444298
101	D/E	COSM335617	2:2348461082:234846108	C/A	0	somatic_SNV	COSMIC	0	Missense variant	1deleterious(0)	997probably damaging(0.996)	ENST00000324695
101	D/E	COSM335617	2:2348461082:234846108	C/A	0	somatic_SNV	COSMIC	0	NMD transcript variant, Missense variant	1deleterious(0)	999probably damaging(0.998)	ENST00000444298
101	Y/H	TMP_ESP_2_234862657	2:2348626572:234862657	T/C	0	SNP	ESP	ESP,	Missense variant	1deleterious(0)	1unknown(0)	ENST00000433712
102	A/T	rs200565245	2:2348461092:234846109	G/A	0	SNP	dbSNP	0	Missense variant	11deleterious(0.01)	997probably damaging(0.996)	ENST00000324695
102	A/T	rs200565245	2:2348461092:234846109	G/A	0	SNP	dbSNP	0	NMD transcript variant, Missense variant	11deleterious(0.01)	999probably damaging(0.998)	ENST00000444298
102	A/T	COSM217237	2:2348477472:234847747	G/A	0	somatic_SNV	COSMIC	0	Missense variant	361tolerated(0.36)	762possibly damaging(0.761)	ENST00000355722
103	K/E	rs150424704	2:2348477502:234847750	A/G	0	SNP	dbSNP	ESP,	Missense variant	221tolerated(0.22)	335benign(0.334)	ENST00000355722
103	L/F	rs201672489	2:2348512312:234851231	C/T	0	SNP	dbSNP	0	Missense variant	551tolerated(0.55)	15benign(0.014)	ENST00000409625
104	T/M	rs200249534	2:2348512352:234851235	C/T	0	SNP	dbSNP	0	Missense variant	1deleterious(0)	1000probably damaging(0.999)	ENST00000409625
104	T/M	COSM1406470	2:2348512352:234851235	C/T	0	somatic_SNV	COSMIC	0	Missense variant	1deleterious(0)	1000probably damaging(0.999)	ENST00000409625
107	S/N	rs7593557	2:2348637882:234863788	G/A	0.301 (A)	SNP	dbSNP	Multiple_observations,Frequency,HapMap,1000Genomes,ESP,	Missense variant	501tolerated(0.5)	1unknown(0)	ENST00000433712
107	S/N	COSM149104	2:2348637882:234863788	G/A	0	somatic_SNV	COSMIC	0	Missense variant	501tolerated(0.5)	1unknown(0)	ENST00000433712
107	R/K	rs202225286	2:2348919082:234891908	G/A	0	SNP	dbSNP	0	NMD transcript variant, Missense variant	481tolerated(0.48)	394benign(0.393)	ENST00000439148
108	K/T	rs199716904	2:2348477662:234847766	A/C	0	SNP	dbSNP	ESP,	Missense variant	61tolerated(0.06)	378benign(0.377)	ENST00000355722
110	D/E	rs200879817	2:2348637982:234863798	C/A	0	SNP	dbSNP	0	Missense variant	651tolerated(0.65)	1unknown(0)	ENST00000433712
111	M/R	rs200821010	2:2348477752:234847775	T/G	0	SNP	dbSNP	0	Missense variant	1deleterious(0)	76benign(0.075)	ENST00000355722
111	K/N	rs151229928	2:2348638012:234863801	G/C	0	SNP	dbSNP	ESP,	Missense variant	181tolerated(0.18)	1unknown(0)	ENST00000433712
111	S/I	rs148696315	2:2348905192:234890519	G/T	0.001 (T)	SNP	dbSNP	1000Genomes,	Missense variant	11deleterious(0.01)	950probably damaging(0.949)	ENST00000456930

113	K/N	COSM264218	2:2348477822:234847782	G/T	0	somatic_SNV	COSMIC	0	Missense variant	171tolerated(0.17)	82benign(0.081)	ENST00000355722
113	K/R	rs202220989	2:2348512622:234851262	A/G	0	SNP	dbSNP	ESP,	Missense variant	331tolerated(0.33)	430benign(0.429)	ENST00000409625
114	Y/C	rs35124867	2:2348512652:234851265	A/G	0	SNP	dbSNP	Multiple_observations,Frequency,	Missense variant	1deleterious(0)	66benign(0.065)	ENST00000409625
115	F/S	rs201611229	2:2348477872:234847787	T/C	0	SNP	dbSNP	0	Missense variant	1deleterious(0)	924probably damaging(0.923)	ENST00000355722
116	S/N	rs190304256	2:2348477902:234847790	G/A	0.001 (A)	SNP	dbSNP	0	Missense variant	351tolerated(0.35)	115benign(0.114)	ENST00000355722
116	S/R	rs199553680	2:2348477912:234847791	C/T/A	0.001 (T)	SNP	dbSNP	ESP,	Missense variant	1001tolerated(1)	4benign(0.003)	ENST00000355722
116	G/R	rs199598346	2:2348512702:234851270	G/A	0	SNP	dbSNP	0	Missense variant	1deleterious(0)	1000probably damaging(0.999)	ENST00000409625
116	P/L	rs199828215	2:2348905342:234890534	C/T	0	SNP	dbSNP	ESP,	Missense variant	1deleterious(0)	1000probably damaging(0.999)	ENST00000456930
117	R/W	rs138240187	2:2348477922:234847792	C/T	0	SNP	dbSNP	Frequency,ESP,	Missense variant	1deleterious(0)	989probably damaging(0.988)	ENST00000355722
118	L/M	rs144120368	2:2348638202:234863820	C/A	0	SNP	dbSNP	Frequency,ESP,	Missense variant	1deleterious(0)	1unknown(0)	ENST00000433712
120	R/G	rs142264059	2:2348512822:234851282	A/G	0	SNP	dbSNP	Multiple_observations,Frequency,	Missense variant	1deleterious(0)	239benign(0.238)	ENST00000409625
121	D/Y	COSM1230577	2:2348512852:234851285	G/T	0	somatic_SNV	COSMIC	0	Missense variant	1deleterious(0)	983probably damaging(0.982)	ENST00000409625
122	Q/L	rs111316052	2:2348905522:234890552	A/T	0	SNP	dbSNP	Frequency,	Missense variant	81tolerated(0.08)	724possibly damaging(0.723)	ENST00000456930
124	S/Y	COSM336694	2:2348478142:234847814	C/A	0	somatic_SNV	COSMIC	0	Missense variant	1deleterious(0)	337benign(0.336)	ENST00000355722
124	I/T	TMP_ESP_2_234851295	2:2348512952:234851295	T/C	0	SNP	ESP	ESP,	Missense variant	271tolerated(0.27)	72benign(0.071)	ENST00000409625
124	M/I	COSM371853	2:2348905592:234890559	G/A	0	somatic_SNV	COSMIC	0	Splice region variant, Missense variant	1deleterious(0)	638possibly damaging(0.637)	ENST00000456930
125	N/S	TMP_ESP_2_234863842	2:2348638422:234863842	A/G	0	SNP	ESP	ESP,	Missense variant	11deleterious(0.01)	1unknown(0)	ENST00000433712
127	E/D	rs138133683	2:2348476742:234847674	A/C	0	SNP	dbSNP	Frequency,ESP,	Missense variant	231tolerated(0.23)	517possibly damaging(0.516)	ENST00000324695
127	E/D	rs138133683	2:2348476742:234847674	A/C	0	SNP	dbSNP	Frequency,ESP,	NMD transcript variant, Missense variant	351tolerated(0.35)	621possibly damaging(0.62)	ENST00000444298
127	L/P	rs140612583	2:2348638482:234863848	T/C	0.001 (C)	SNP	dbSNP	Multiple_observations,Frequency,	Missense variant	11deleterious(0.01)	1unknown(0)	ENST00000433712
130	L/F	rs201672489	2:2348512312:234851231	C/T	0	SNP	dbSNP	0	Missense variant	711tolerated(0.71)	15benign(0.014)	ENST00000355722
130	T/M	rs114456097	2:2348919772:234891977	C/T	0.005 (T)	SNP	dbSNP	Multiple_observations,Frequency,1000Genomes,	NMD transcript variant, Missense variant	0	1unknown(0)	ENST00000439148

131	T/M	rs200249534	2:2348512352:234851235	C/T	0	SNP	dbSNP	0	Missense variant	1deleterious(0)	1000probably damaging(0.999)	ENST00000355722
131	T/M	COSM1406470	2:2348512352:234851235	C/T	0	somatic_SNV	COSMIC	0	Missense variant	1deleterious(0)	1000probably damaging(0.999)	ENST00000355722
131	N/T	rs143385407	2:2348638602:234863860	A/C	0	SNP	dbSNP	Multiple_observations,Frequency,ESP,	Missense variant	111tolerated(0.11)	1unknown(0)	ENST00000433712
133	N/S	rs11563210	2:2348919862:234891986	A/G	0.011 (G)	SNP	dbSNP	Multiple_observations,Frequency,HapMap,1000Genomes,	NMD transcript variant, Missense variant	0	1unknown(0)	ENST00000439148
134	A/T	COSM1406471	2:2348513242:234851324	G/A	0	somatic_SNV	COSMIC	0	Missense variant	811tolerated(0.81)	108benign(0.107)	ENST00000409625
137	I/V	COSM1406472	2:2348513332:234851333	A/G	0	somatic_SNV	COSMIC	0	Missense variant	251tolerated(0.25)	394benign(0.393)	ENST00000409625
137	I/M	rs199520024	2:2348513352:234851335	A/G	0	SNP	dbSNP	0	Missense variant	11deleterious(0.01)	963probably damaging(0.962)	ENST00000409625
138	W/R	TMP_ESP_2_234891736	2:2348917362:234891736	T/C	0	SNP	ESP	ESP,	Missense variant	1deleterious(0)	758possibly damaging(0.757)	ENST00000456930
139	R/S	rs182733234	2:2348638832:234863883	C/T/A	0.001 (T)	SNP	dbSNP	ESP,	Missense variant	451tolerated(0.45)	1unknown(0)	ENST00000433712
139	R/C	rs182733234	2:2348638832:234863883	C/T/A	0.001 (T)	SNP	dbSNP	ESP,	Missense variant	181tolerated(0.18)	1unknown(0)	ENST00000433712
139	V/I	rs201464699	2:2348943392:234894339	G/A	0	SNP	dbSNP	0	NMD transcript variant, Missense variant	0	1unknown(0)	ENST00000439148
140	K/R	rs202220989	2:2348512622:234851262	A/G	0	SNP	dbSNP	ESP,	Missense variant	351tolerated(0.35)	430benign(0.429)	ENST00000355722
140	R/Q	rs200547297	2:2348638872:234863887	G/A	0	SNP	dbSNP	0	Missense variant	771tolerated(0.77)	1unknown(0)	ENST00000433712
140	R/Q	COSM1406474	2:2348638872:234863887	G/A	0	somatic_SNV	COSMIC	0	Missense variant	771tolerated(0.77)	1unknown(0)	ENST00000433712
140	V/A	rs201253609	2:2348917432:234891743	T/C	0	SNP	dbSNP	0	Missense variant	1deleterious(0)	944probably damaging(0.943)	ENST00000456930
141	Y/C	rs35124867	2:2348512652:234851265	A/G	0	SNP	dbSNP	Multiple_observations,Frequency,	Missense variant	1deleterious(0)	66benign(0.065)	ENST00000355722
142	E/K	rs201971040	2:2348638922:234863892	G/A	0	SNP	dbSNP	0	Splice region variant, Missense variant	1001tolerated(1)	1unknown(0)	ENST00000433712
143	G/R	rs199598346	2:2348512702:234851270	G/A	0	SNP	dbSNP	0	Missense variant	1deleterious(0)	1000probably damaging(0.999)	ENST00000355722
144	L/P	rs201249162	2:2348477242:234847724	T/C	0	SNP	dbSNP	0	Missense variant	1deleterious(0)	1000probably damaging(0.999)	ENST00000324695
144	L/P	rs201249162	2:2348477242:234847724	T/C	0	SNP	dbSNP	0	NMD transcript variant, Missense variant	1deleterious(0)	963probably damaging(0.962)	ENST00000444298
145	N/K	rs200551327	2:2348513592:234851359	C/G	0	SNP	dbSNP	0	Missense variant	31deleterious(0.03)	428benign(0.427)	ENST00000409625
146	R/L	COSM1531361	2:2348513612:234851361	G/T	0	somatic_SNV	COSMIC	0	Missense variant	1deleterious(0)	980probably damaging(0.979)	ENST00000409625

147	R/G	rs142264059	2:2348512822:234851282	A/G	0	SNP	dbSNP	Multiple_observations,Frequency,	Missense variant	1deleterious(0)	239benign(0.238)	ENST00000355722
147	Q/R	rs145289688	2:2348694332:234869433	A/G	0.001 (G)	SNP	dbSNP	1000Genomes,	Missense variant	21deleterious(0.02)	1unknown(0)	ENST00000433712
148	D/Y	COSM1230577	2:2348512852:234851285	G/T	0	somatic_SNV	COSMIC	0	Missense variant	1deleterious(0)	983probably damaging(0.982)	ENST00000355722
150	G/R	rs199497591	2:2348477412:234847741	G/C	0	SNP	dbSNP	0	Missense variant	1deleterious(0)	1000probably damaging(0.999)	ENST00000324695
150	G/R	rs199497591	2:2348477412:234847741	G/C	0	SNP	dbSNP	0	NMD transcript variant, Missense variant	1deleterious(0)	965probably damaging(0.964)	ENST00000444298
150	M/T	rs28902173	2:2348694422:234869442	T/C	0.003 (C)	SNP	dbSNP	Multiple_observations,Frequency,HapMap,1000Genomes,ESP,	Missense variant	1deleterious(0)	1unknown(0)	ENST00000433712
151	I/T	TMP_ESP_2_234851295	2:2348512952:234851295	T/C	0	SNP	ESP	ESP,	Missense variant	341tolerated(0.34)	72benign(0.071)	ENST00000355722
151	R/S	COSM324023	2:2348513772:234851377	G/T	0	somatic_SNV	COSMIC	0	Missense variant	281tolerated(0.28)	72benign(0.071)	ENST00000409625
152	A/T	COSM217237	2:2348477472:234847747	G/A	0	somatic_SNV	COSMIC	0	Missense variant	21deleterious(0.02)	969probably damaging(0.968)	ENST00000324695
152	A/T	COSM217237	2:2348477472:234847747	G/A	0	somatic_SNV	COSMIC	0	NMD transcript variant, Missense variant	11deleterious(0.01)	999probably damaging(0.998)	ENST00000444298
152	T/M	rs143538093	2:2348694482:234869448	C/T	0	SNP	dbSNP	Multiple_observations,Frequency,ESP,	Missense variant	41deleterious(0.04)	1unknown(0)	ENST00000433712
153	K/E	rs150424704	2:2348477502:234847750	A/G	0	SNP	dbSNP	ESP,	Missense variant	61tolerated(0.06)	363benign(0.362)	ENST00000324695
153	K/E	rs150424704	2:2348477502:234847750	A/G	0	SNP	dbSNP	ESP,	NMD transcript variant, Missense variant	51tolerated(0.05)	258benign(0.257)	ENST00000444298
155	I/V	rs201369256	2:2348694562:234869456	A/G	0	SNP	dbSNP	0	Missense variant	701tolerated(0.7)	1unknown(0)	ENST00000433712
156	R/H	TMP_ESP_2_234891791	2:2348917912:234891791	G/A	0	SNP	ESP	ESP,	Missense variant	21deleterious(0.02)	1000probably damaging(0.999)	ENST00000456930
158	K/T	rs199716904	2:2348477662:234847766	A/C	0	SNP	dbSNP	ESP,	Missense variant	1deleterious(0)	961probably damaging(0.96)	ENST00000324695
158	K/T	rs199716904	2:2348477662:234847766	A/C	0	SNP	dbSNP	ESP,	NMD transcript variant, Missense variant	11deleterious(0.01)	998probably damaging(0.997)	ENST00000444298
158	P/L	rs200891977	2:2348513972:234851397	C/T	0	SNP	dbSNP	ESP,	Missense variant	31deleterious(0.03)	99benign(0.098)	ENST00000409625
159	P/S	TMP_ESP_2_234869468	2:2348694682:234869468	C/T	0	SNP	ESP	ESP,	Missense variant	1deleterious(0)	1unknown(0)	ENST00000433712
161	M/R	rs200821010	2:2348477752:234847775	T/G	0	SNP	dbSNP	0	Missense variant	1deleterious(0)	473possibly damaging(0.472)	ENST00000324695
161	M/R	rs200821010	2:2348477752:234847775	T/G	0	SNP	dbSNP	0	NMD transcript variant, Missense variant	1deleterious(0)	760possibly damaging(0.759)	ENST00000444298
161	A/T	COSM1406471	2:2348513242:234851324	G/A	0	somatic_SNV	COSMIC	0	Missense variant	781tolerated(0.78)	108benign(0.107)	ENST00000355722

161	F/L	rs113858305	2:2348694742:234869474	T/C	0	SNP	dbSNP	Frequency,	Missense variant	1deleterious(0)	1unknown(0)	ENST00000433712
162	R/C	rs201721376	2:2348918082:234891808	C/T	0	SNP	dbSNP	0	Missense variant	1deleterious(0)	999probably damaging(0.998)	ENST00000456930
162	R/H	rs199784199	2:2348918092:234891809	G/A	0	SNP	dbSNP	0	Missense variant	11deleterious(0.01)	752possibly damaging(0.751)	ENST00000456930
163	K/N	COSM264218	2:2348477822:234847782	G/T	0	somatic_SNV	COSMIC	0	Missense variant	991tolerated(0.99)	961probably damaging(0.96)	ENST00000324695
163	K/N	COSM264218	2:2348477822:234847782	G/T	0	somatic_SNV	COSMIC	0	NMD transcript variant, Missense variant	521tolerated(0.52)	998probably damaging(0.997)	ENST00000444298
163	R/C	rs201940567	2:2348694802:234869480	C/T	0.001 (T)	SNP	dbSNP	0	Missense variant	1deleterious(0)	1unknown(0)	ENST00000433712
163	R/H	TMP_ESP_2_234869481	2:2348694812:234869481	G/A	0	SNP	ESP	ESP,	Missense variant	11deleterious(0.01)	1unknown(0)	ENST00000433712
163	S/L	COSM171795	2:2348918122:234891812	C/T	0	somatic_SNV	COSMIC	0	Missense variant	111tolerated(0.11)	871possibly damaging(0.87)	ENST00000456930
164	I/V	COSM1406472	2:2348513332:234851333	A/G	0	somatic_SNV	COSMIC	0	Missense variant	231tolerated(0.23)	394benign(0.393)	ENST00000355722
164	I/M	rs199520024	2:2348513352:234851335	A/G	0	SNP	dbSNP	0	Missense variant	11deleterious(0.01)	963probably damaging(0.962)	ENST00000355722
165	F/S	rs201611229	2:2348477872:234847787	T/C	0	SNP	dbSNP	0	Missense variant	1deleterious(0)	136benign(0.135)	ENST00000324695
165	F/S	rs201611229	2:2348477872:234847787	T/C	0	SNP	dbSNP	0	NMD transcript variant, Missense variant	1deleterious(0)	337benign(0.336)	ENST00000444298
166	S/N	rs190304256	2:2348477902:234847790	G/A	0.001 (A)	SNP	dbSNP	0	Missense variant	31deleterious(0.03)	438possibly damaging(0.437)	ENST00000324695
166	S/N	rs190304256	2:2348477902:234847790	G/A	0.001 (A)	SNP	dbSNP	0	NMD transcript variant, Missense variant	31deleterious(0.03)	742possibly damaging(0.741)	ENST00000444298
166	S/R	rs199553680	2:2348477912:234847791	C/T/A	0.001 (T)	SNP	dbSNP	ESP,	Missense variant	1001tolerated(1)	13benign(0.012)	ENST00000324695
166	S/R	rs199553680	2:2348477912:234847791	C/T/A	0.001 (T)	SNP	dbSNP	ESP,	NMD transcript variant, Missense variant	1001tolerated(1)	68benign(0.067)	ENST00000444298
167	R/W	rs138240187	2:2348477922:234847792	C/T	0	SNP	dbSNP	Frequency,ESP,	Missense variant	1deleterious(0)	998probably damaging(0.997)	ENST00000324695
167	R/W	rs138240187	2:2348477922:234847792	C/T	0	SNP	dbSNP	Frequency,ESP,	NMD transcript variant, Missense variant	1deleterious(0)	1000probably damaging(0.999)	ENST00000444298
168	N/S	TMP_ESP_2_234869496	2:2348694962:234869496	A/G	0	SNP	ESP	ESP,	Missense variant	21deleterious(0.02)	1unknown(0)	ENST00000433712
169	G/D	COSM1494873	2:2348694992:234869499	G/A	0	somatic_SNV	COSMIC	0	Missense variant	1deleterious(0)	1unknown(0)	ENST00000433712
172	N/K	rs200551327	2:2348513592:234851359	C/G	0	SNP	dbSNP	0	Missense variant	31deleterious(0.03)	428benign(0.427)	ENST00000355722
173	R/L	COSM1531361	2:2348513612:234851361	G/T	0	somatic_SNV	COSMIC	0	Missense variant	1deleterious(0)	980probably damaging(0.979)	ENST00000355722
173	R/W	rs111972494	2:2348695102:234869510	C/T	0	SNP	dbSNP	Multiple_observations,Frequency,ESP,	Missense variant	61tolerated(0.06)	1unknown(0)	ENST00000433712

173	R/Q	rs200772894	2:2348695112:234869511	G/A	0.001 (A)	SNP	dbSNP	0	Missense variant	511tolerated(0.51)	1unknown(0)	ENST00000433712
174	S/Y	COSM336694	2:2348478142:234847814	C/A	0	somatic_SNV	COSMIC	0	Missense variant	1deleterious(0)	891possibly damaging(0.89)	ENST00000324695
174	S/Y	COSM336694	2:2348478142:234847814	C/A	0	somatic_SNV	COSMIC	0	NMD transcript variant, Missense variant	1deleterious(0)	608possibly damaging(0.607)	ENST00000444298
176	V/M	rs149328116	2:2348918502:234891850	G/A	0.001 (A)	SNP	dbSNP	Multiple_observations,Frequency,ESP,	Missense variant	31deleterious(0.03)	69benign(0.068)	ENST00000456930
177	T/I	rs76512385	2:2348695232:234869523	C/T	0.001 (T)	SNP	dbSNP	Multiple_observations,Frequency,1000 Genomes,ESP,	Missense variant	1deleterious(0)	1unknown(0)	ENST00000433712
178	R/S	COSM324023	2:2348513772:234851377	G/T	0	somatic_SNV	COSMIC	0	Missense variant	121tolerated(0.12)	72benign(0.071)	ENST00000355722
179	D/G	rs199871046	2:2348695292:234869529	A/G	0	SNP	dbSNP	0	Missense variant	431tolerated(0.43)	1unknown(0)	ENST00000433712
180	L/F	rs201672489	2:2348512312:234851231	C/T	0	SNP	dbSNP	0	Missense variant	331tolerated(0.33)	3benign(0.002)	ENST00000324695
180	L/F	rs201672489	2:2348512312:234851231	C/T	0	SNP	dbSNP	0	NMD transcript variant, Missense variant	241tolerated(0.24)	9benign(0.008)	ENST00000444298
180	V/F	rs200514662	2:2348695312:234869531	G/T	0	SNP	dbSNP	0	Missense variant	101tolerated(0.1)	1unknown(0)	ENST00000433712
180	V/M	rs199993016	2:2348918622:234891862	G/A	0	SNP	dbSNP	0	Missense variant	421tolerated(0.42)	951probably damaging(0.95)	ENST00000456930
180	V/M	COSM210455	2:2348918622:234891862	G/A	0	somatic_SNV	COSMIC	0	Missense variant	421tolerated(0.42)	951probably damaging(0.95)	ENST00000456930
181	T/M	rs200249534	2:2348512352:234851235	C/T	0	SNP	dbSNP	0	Missense variant	1deleterious(0)	998probably damaging(0.997)	ENST00000324695
181	T/M	COSM1406470	2:2348512352:234851235	C/T	0	somatic_SNV	COSMIC	0	Missense variant	1deleterious(0)	998probably damaging(0.997)	ENST00000324695
181	T/M	rs200249534	2:2348512352:234851235	C/T	0	SNP	dbSNP	0	NMD transcript variant, Missense variant	1deleterious(0)	1000probably damaging(0.999)	ENST00000444298
181	T/M	COSM1406470	2:2348512352:234851235	C/T	0	somatic_SNV	COSMIC	0	NMD transcript variant, Missense variant	1deleterious(0)	1000probably damaging(0.999)	ENST00000444298
181	L/F	rs201464095	2:2348695342:234869534	C/T	0	SNP	dbSNP	0	Missense variant	1deleterious(0)	1unknown(0)	ENST00000433712
182	T/I	rs200065004	2:2348695382:234869538	C/T	0	SNP	dbSNP	0	Missense variant	461tolerated(0.46)	1unknown(0)	ENST00000433712
182	G/S	rs201072281	2:2348918682:234891868	G/A	0	SNP	dbSNP	0	Splice region variant, Missense variant	451tolerated(0.45)	322benign(0.321)	ENST00000456930
184	T/M	rs200355253	2:2348943382:234894338	C/T	0	SNP	dbSNP	0	Missense variant	101tolerated(0.1)	904possibly damaging(0.903)	ENST00000456930
185	P/L	rs200891977	2:2348513972:234851397	C/T	0	SNP	dbSNP	ESP,	Missense variant	41deleterious(0.04)	99benign(0.098)	ENST00000355722
185	F/L	rs200953031	2:2348695462:234869546	T/C	0	SNP	dbSNP	0	Missense variant	11deleterious(0.01)	1unknown(0)	ENST00000433712

188	H/Y	TMP_ESP_2_234869555	2:2348695552:234869555	C/T	0	SNP	ESP	ESP,	Missense variant	1001tolerated(1)	1unknown(0)	ENST00000433712
188	A/V	rs201934673	2:2348943502:234894350	C/T	0	SNP	dbSNP	0	Missense variant	371tolerated(0.37)	149benign(0.148)	ENST00000456930
190	K/R	rs202220989	2:2348512622:234851262	A/G	0	SNP	dbSNP	ESP,	Missense variant	161tolerated(0.16)	967probably damaging(0.966)	ENST00000324695
190	K/R	rs202220989	2:2348512622:234851262	A/G	0	SNP	dbSNP	ESP,	NMD transcript variant, Missense variant	81tolerated(0.08)	994probably damaging(0.993)	ENST00000444298
191	Y/C	rs35124867	2:2348512652:234851265	A/G	0	SNP	dbSNP	Multiple_observations,Frequency,	Missense variant	151tolerated(0.15)	835possibly damaging(0.834)	ENST00000324695
191	Y/C	rs35124867	2:2348512652:234851265	A/G	0	SNP	dbSNP	Multiple_observations,Frequency,	NMD transcript variant, Missense variant	41deleterious(0.04)	885possibly damaging(0.884)	ENST00000444298
191	T/M	rs149254613	2:2348695652:234869565	C/T	0	SNP	dbSNP	Multiple_observations,Frequency,ESP,	Missense variant	31deleterious(0.03)	1unknown(0)	ENST00000433712
193	G/R	rs199598346	2:2348512702:234851270	G/A	0	SNP	dbSNP	0	Missense variant	1deleterious(0)	1000probably damaging(0.999)	ENST00000324695
193	G/R	rs199598346	2:2348512702:234851270	G/A	0	SNP	dbSNP	0	NMD transcript variant, Missense variant	1deleterious(0)	1000probably damaging(0.999)	ENST00000444298
195	R/W	COSM1406476	2:2348695762:234869576	C/T	0	somatic_SNV	COSMIC	0	Missense variant	21deleterious(0.02)	1unknown(0)	ENST00000433712
195	R/Q	rs201095192	2:2348695772:234869577	G/A	0	SNP	dbSNP	0	Missense variant	291tolerated(0.29)	1unknown(0)	ENST00000433712
197	R/G	rs142264059	2:2348512822:234851282	A/G	0	SNP	dbSNP	Multiple_observations,Frequency,	Missense variant	1deleterious(0)	992probably damaging(0.991)	ENST00000324695
197	R/G	rs142264059	2:2348512822:234851282	A/G	0	SNP	dbSNP	Multiple_observations,Frequency,	NMD transcript variant, Missense variant	1deleterious(0)	997probably damaging(0.996)	ENST00000444298
198	D/Y	COSM1230577	2:2348512852:234851285	G/T	0	somatic_SNV	COSMIC	0	Missense variant	1deleterious(0)	1000probably damaging(0.999)	ENST00000324695
198	D/Y	COSM1230577	2:2348512852:234851285	G/T	0	somatic_SNV	COSMIC	0	NMD transcript variant, Missense variant	1deleterious(0)	1000probably damaging(0.999)	ENST00000444298
200	A/T	rs200884995	2:2348695912:234869591	G/A	0	SNP	dbSNP	0	Missense variant	451tolerated(0.45)	1unknown(0)	ENST00000433712
200	A/T	COSM210453	2:2348695912:234869591	G/A	0	somatic_SNV	COSMIC	0	Missense variant	451tolerated(0.45)	1unknown(0)	ENST00000433712
200	L/M	rs139760142	2:2348943852:234894385	C/A	0.001 (A)	SNP	dbSNP	Multiple_observations,1000Genomes,	Missense variant	221tolerated(0.22)	495possibly damaging(0.494)	ENST00000456930
201	I/T	TMP_ESP_2_234851295	2:2348512952:234851295	T/C	0	SNP	ESP	ESP,	Missense variant	81tolerated(0.08)	643possibly damaging(0.642)	ENST00000324695
201	I/T	TMP_ESP_2_234851295	2:2348512952:234851295	T/C	0	SNP	ESP	ESP,	NMD transcript variant, Missense variant	141tolerated(0.14)	997probably damaging(0.996)	ENST00000444298
201	K/N	COSM277842	2:2348695962:234869596	G/T	0	somatic_SNV	COSMIC	0	Missense variant	161tolerated(0.16)	1unknown(0)	ENST00000433712
207	H/R	rs199727864	2:2348944072:234894407	A/G	0	SNP	dbSNP	0	Missense variant	671tolerated(0.67)	15benign(0.014)	ENST00000456930
210	T/M	COSM1018191	2:2348696222:234869622	C/T	0	somatic_SNV	COSMIC	0	Missense variant	221tolerated(0.22)	1unknown(0)	ENST00000433712

210	P/S	rs200680058	2:2348944152:234894415	C/T	0	SNP	dbSNP	0	Missense variant	261tolerated(0.26)	757possibly damaging(0.756)	ENST00000456930
211	A/T	COSM1406471	2:2348513242:234851324	G/A	0	somatic_SNV	COSMIC	0	Missense variant	681tolerated(0.68)	996probably damaging(0.995)	ENST00000324695
211	A/T	COSM1406471	2:2348513242:234851324	G/A	0	somatic_SNV	COSMIC	0	NMD transcript variant, Missense variant	531tolerated(0.53)	999probably damaging(0.998)	ENST00000444298
211	R/W	rs147248663	2:2348944182:234894418	C/T	0	SNP	dbSNP	Multiple_observations,Frequency,ESP,	Missense variant	31deleterious(0.03)	932probably damaging(0.931)	ENST00000456930
211	R/Q	rs199616111	2:2348944192:234894419	G/T/A	0	SNP	dbSNP	0	Missense variant	611tolerated(0.61)	862possibly damaging(0.861)	ENST00000456930
211	R/L	rs199616111	2:2348944192:234894419	G/T/A	0	SNP	dbSNP	0	Missense variant	351tolerated(0.35)	74benign(0.073)	ENST00000456930
213	P/S	rs200606227	2:2348944242:234894424	C/T	0	SNP	dbSNP	0	Missense variant	151tolerated(0.15)	990probably damaging(0.989)	ENST00000456930
214	I/V	COSM1406472	2:2348513332:234851333	A/G	0	somatic_SNV	COSMIC	0	Missense variant	431tolerated(0.43)	25benign(0.024)	ENST00000324695
214	I/V	COSM1406472	2:2348513332:234851333	A/G	0	somatic_SNV	COSMIC	0	NMD transcript variant, Missense variant	391tolerated(0.39)	261benign(0.26)	ENST00000444298
214	I/M	rs199520024	2:2348513352:234851335	A/G	0	SNP	dbSNP	0	Missense variant	1deleterious(0)	366benign(0.365)	ENST00000324695
214	I/M	rs199520024	2:2348513352:234851335	A/G	0	SNP	dbSNP	0	NMD transcript variant, Missense variant	1deleterious(0)	686possibly damaging(0.685)	ENST00000444298
214	E/K	rs202035579	2:2348944272:234894427	G/A	0	SNP	dbSNP	0	Missense variant	131tolerated(0.13)	900possibly damaging(0.899)	ENST00000456930
218	N/K	COSM1230576	2:2348696472:234869647	C/A	0	somatic_SNV	COSMIC	0	Missense variant	181tolerated(0.18)	1unknown(0)	ENST00000433712
220	R/Q	rs201941621	2:2348696522:234869652	G/A	0	SNP	dbSNP	ESP,	Missense variant	441tolerated(0.44)	1unknown(0)	ENST00000433712
222	N/K	rs200551327	2:2348513592:234851359	C/G	0	SNP	dbSNP	0	Missense variant	11deleterious(0.01)	992probably damaging(0.991)	ENST00000324695
222	N/K	rs200551327	2:2348513592:234851359	C/G	0	SNP	dbSNP	0	NMD transcript variant, Missense variant	61tolerated(0.06)	997probably damaging(0.996)	ENST00000444298
223	R/L	COSM1531361	2:2348513612:234851361	G/T	0	somatic_SNV	COSMIC	0	Missense variant	1deleterious(0)	996probably damaging(0.995)	ENST00000324695
223	R/L	COSM1531361	2:2348513612:234851361	G/T	0	somatic_SNV	COSMIC	0	NMD transcript variant, Missense variant	1deleterious(0)	999probably damaging(0.998)	ENST00000444298
224	R/W	rs201447068	2:2348696632:234869663	C/T	0	SNP	dbSNP	0	Missense variant	191tolerated(0.19)	1unknown(0)	ENST00000433712
224	R/Q	rs199756522	2:2348696642:234869664	G/A	0	SNP	dbSNP	ESP,	Missense variant	481tolerated(0.48)	1unknown(0)	ENST00000433712
225	K/N	COSM108177	2:2348696682:234869668	G/T	0	somatic_SNV	COSMIC	0	Missense variant	121tolerated(0.12)	1unknown(0)	ENST00000433712
225	K/N	rs149963916	2:2348696682:234869668	G/T	0	somatic_SNV	dbSNP	0	Missense variant	121tolerated(0.12)	1unknown(0)	ENST00000433712
228	R/S	COSM324023	2:2348513772:234851377	G/T	0	somatic_SNV	COSMIC	0	Missense variant	211tolerated(0.21)	935probably damaging(0.934)	ENST00000324695

228	R/S	COSM324023	2:2348513772:234851377	G/T	0	somatic_SNV	COSMIC	0	NMD transcript variant, Missense variant	231tolerated(0.23)	997probably damaging(0.996)	ENST00000444298
231	R/W	rs200707338	2:2348696842:234869684	C/T	0	SNP	dbSNP	0	Missense variant	31deleterious(0.03)	1unknown(0)	ENST00000433712
231	R/W	COSM1565678	2:2348696842:234869684	C/T	0	somatic_SNV	COSMIC	0	Missense variant	31deleterious(0.03)	1unknown(0)	ENST00000433712
231	R/Q	rs201047162	2:2348696852:234869685	G/A	0	SNP	dbSNP	0	Missense variant	471tolerated(0.47)	1unknown(0)	ENST00000433712
232	D/E	rs142882642	2:2348696892:234869689	C/G/T	0	SNP	dbSNP	Multiple_observations,Frequency,ESP,	Missense variant	931tolerated(0.93)	1unknown(0)	ENST00000433712
233	E/K	rs200725140	2:2348696902:234869690	G/A	0	SNP	dbSNP	0	Missense variant	871tolerated(0.87)	1unknown(0)	ENST00000433712
236	I/V	rs200180972	2:2348696992:234869699	A/G	0	SNP	dbSNP	0	Missense variant	631tolerated(0.63)	1unknown(0)	ENST00000433712
237	E/K	rs200749841	2:2348697022:234869702	G/A	0	SNP	dbSNP	0	Missense variant	921tolerated(0.92)	1unknown(0)	ENST00000433712
238	A/T	rs202029767	2:2348545122:234854512	G/A	0	SNP	dbSNP	0	Missense variant	1deleterious(0)	551possibly damaging(0.55)	ENST00000324695
238	A/T	rs202029767	2:2348545122:234854512	G/A	0	SNP	dbSNP	0	NMD transcript variant, Missense variant	1deleterious(0)	755possibly damaging(0.754)	ENST00000444298
241	L/V	rs200473694	2:2348545212:234854521	C/G	0	SNP	dbSNP	0	Missense variant	961tolerated(0.96)	1benign(0)	ENST00000324695
241	L/V	rs200473694	2:2348545212:234854521	C/G	0	SNP	dbSNP	0	NMD transcript variant, Missense variant	591tolerated(0.59)	3benign(0.002)	ENST00000444298
243	T/M	rs202017617	2:2349049752:234904975	C/T	0	SNP	dbSNP	0	Missense variant	1deleterious(0)	1001probably damaging(1)	ENST00000456930
244	V/M	TMP_ESP_2_234904977	2:2349049772:234904977	G/A	0	SNP	ESP	ESP,	Missense variant	1deleterious(0)	982probably damaging(0.981)	ENST00000456930
246	R/W	rs202225959	2:2348719442:234871944	C/T	0	SNP	dbSNP	0	Missense variant	81tolerated(0.08)	1unknown(0)	ENST00000433712
246	R/Q	rs201115161	2:2348719452:234871945	G/A/T	0.001 (A)	SNP	dbSNP	ESP,	Missense variant	601tolerated(0.6)	1unknown(0)	ENST00000433712
246	R/L	rs201115161	2:2348719452:234871945	G/A/T	0.001 (A)	SNP	dbSNP	ESP,	Missense variant	471tolerated(0.47)	1unknown(0)	ENST00000433712
246	R/Q	COSM1482913	2:2348719452:234871945	G/A	0	somatic_SNV	COSMIC	0	Missense variant	601tolerated(0.6)	1unknown(0)	ENST00000433712
247	R/T	rs13004520	2:2348545402:234854540	G/C	0.058 (C)	SNP	dbSNP	Multiple_observations,Frequency,HapMap,1000Genomes,ESP,	Missense variant	11deleterious(0.01)	183benign(0.182)	ENST00000324695
247	R/T	rs13004520	2:2348545402:234854540	G/C	0.058 (C)	SNP	dbSNP	Multiple_observations,Frequency,HapMap,1000Genomes,ESP,	NMD transcript variant, Missense variant	121tolerated(0.12)	473possibly damaging(0.472)	ENST00000444298
247	V/I	rs148556306	2:2349049862:234904986	G/A	0	SNP	dbSNP	Multiple_observations,Frequency,ESP,	Missense variant	101tolerated(0.1)	987probably damaging(0.986)	ENST00000456930

248	P/S	rs141865492	2:2348719502:234871950	C/T	0	SNP	dbSNP	Frequency,ESP,	Missense variant	21deleterious(0.02)	1unknown(0)	ENST00000433712
248	Q/R	rs200248747	2:2349049902:234904990	A/G	0	SNP	dbSNP	0	Missense variant	1deleterious(0)	741possibly damaging(0.74)	ENST00000456930
249	P/S	rs201941040	2:2348545452:234854545	C/T	0	SNP	dbSNP	0	Missense variant	581tolerated(0.58)	104benign(0.103)	ENST00000324695
249	P/S	rs201941040	2:2348545452:234854545	C/T	0	SNP	dbSNP	0	NMD transcript variant, Missense variant	861tolerated(0.86)	479possibly damaging(0.478)	ENST00000444298
250	Q/E	COSM1531359	2:2348719562:234871956	C/G	0	somatic_SNV	COSMIC	0	Missense variant	91tolerated(0.09)	1unknown(0)	ENST00000433712
251	Y/C	rs17868387	2:2348545522:234854552	A/G	0.060 (G)	SNP	dbSNP	Multiple_observations,Frequency,HapMap,1000Genomes,ESP,	Missense variant	91tolerated(0.09)	835possibly damaging(0.834)	ENST00000324695
251	Y/C	rs17868387	2:2348545522:234854552	A/G	0.060 (G)	SNP	dbSNP	Multiple_observations,Frequency,HapMap,1000Genomes,ESP,	NMD transcript variant, Missense variant	161tolerated(0.16)	971probably damaging(0.97)	ENST00000444298
253	L/M	COSM324022	2:2348545572:234854557	C/A	0	somatic_SNV	COSMIC	0	Missense variant	1deleterious(0)	999probably damaging(0.998)	ENST00000324695
253	L/M	COSM324022	2:2348545572:234854557	C/A	0	somatic_SNV	COSMIC	0	NMD transcript variant, Missense variant	1deleterious(0)	1000probably damaging(0.999)	ENST00000444298
253	Q/K	rs143128851	2:2349050042:234905004	C/A	0	SNP	dbSNP	Frequency,ESP,	Missense variant	751tolerated(0.75)	346benign(0.345)	ENST00000456930
254	I/M	TMP_ESP_2_234871970	2:2348719702:234871970	C/G	0	SNP	ESP	ESP,	Missense variant	11deleterious(0.01)	1unknown(0)	ENST00000433712
256	K/N	COSM335618	2:2349050152:234905015	G/T	0	somatic_SNV	COSMIC	0	Missense variant	1deleterious(0)	999probably damaging(0.998)	ENST00000456930
257	H/Y	rs201450325	2:2348545692:234854569	C/T	0	SNP	dbSNP	ESP,	Missense variant	121tolerated(0.12)	27benign(0.026)	ENST00000324695
257	H/Y	rs201450325	2:2348545692:234854569	C/T	0	SNP	dbSNP	ESP,	NMD transcript variant, Missense variant	131tolerated(0.13)	760possibly damaging(0.759)	ENST00000444298
259	H/Y	rs199593973	2:2348545752:234854575	C/T	0	SNP	dbSNP	ESP,	Missense variant	1deleterious(0)	983probably damaging(0.982)	ENST00000324695
259	H/Y	rs199593973	2:2348545752:234854575	C/T	0	SNP	dbSNP	ESP,	NMD transcript variant, Missense variant	1deleterious(0)	997probably damaging(0.996)	ENST00000444298
259	Q/R	rs201451492	2:2348719842:234871984	A/G	0	SNP	dbSNP	0	Missense variant	11deleterious(0.01)	1unknown(0)	ENST00000433712
260	N/T	rs201960730	2:2348719872:234871987	A/C	0.001 (C)	SNP	dbSNP	0	Missense variant	1deleterious(0)	1unknown(0)	ENST00000433712
262	L/P	TMP_ESP_2_234905032	2:2349050322:234905032	T/C	0	SNP	ESP	ESP,	Missense variant	1deleterious(0)	1000probably damaging(0.999)	ENST00000456930
265	N/S	COSM1162608	2:2348545942:234854594	A/G	0	somatic_SNV	COSMIC	0	Missense variant	21deleterious(0.02)	125benign(0.124)	ENST00000324695
265	N/S	COSM1162608	2:2348545942:234854594	A/G	0	somatic_SNV	COSMIC	0	NMD transcript variant, Missense variant	11deleterious(0.01)	446possibly damaging(0.445)	ENST00000444298

265	S/F	rs149198571	2:2348720022:234872002	C/T	0	SNP	dbSNP	Frequency,ESP,	Missense variant	1deleterious(0)	1unknown(0)	ENST00000433712
267	V/I	rs201927659	2:2348720072:234872007	G/A	0.001 (A)	SNP	dbSNP	0	Missense variant	1001tolerated(1)	1unknown(0)	ENST00000433712
268	H/Y	rs199515958	2:2348546022:234854602	C/T	0	SNP	dbSNP	ESP,	Missense variant	111tolerated(0.11)	12benign(0.011)	ENST00000324695
268	H/Y	rs199515958	2:2348546022:234854602	C/T	0	SNP	dbSNP	ESP,	NMD transcript variant, Missense variant	221tolerated(0.22)	72benign(0.071)	ENST00000444298
269	R/C	rs199727860	2:2349050522:234905052	C/T	0	SNP	dbSNP	0	Missense variant	1deleterious(0)	1001probably damaging(1)	ENST00000456930
269	R/H	COSM1406478	2:2349050532:234905053	G/A	0	somatic_SNV	COSMIC	0	Missense variant	1deleterious(0)	999probably damaging(0.998	ENST00000456930
273	R/K	rs148231346	2:2348732762:234873276	G/A	0.001 (A)	SNP	dbSNP	Multiple_observations,Frequency,ESP,	Missense variant	491tolerated(0.49)	1unknown(0)	ENST00000433712
274	E/K	COSM110191	2:2348546202:234854620	G/A	0	somatic_SNV	COSMIC	0	Missense variant	11deleterious(0.01)	609possibly damaging(0.608)	ENST00000324695
274	E/K	rs140382067	2:2348546202:234854620	G/A	0	somatic_SNV	dbSNP	Multiple_observations,Cited,ESP,	Missense variant	11deleterious(0.01)	609possibly damaging(0.608)	ENST00000324695
274	E/K	COSM110191	2:2348546202:234854620	G/A	0	somatic_SNV	COSMIC	0	NMD transcript variant, Missense variant	1deleterious(0)	995probably damaging(0.994)	ENST00000444298
274	E/K	rs140382067	2:2348546202:234854620	G/A	0	somatic_SNV	dbSNP	Multiple_observations,Cited,ESP,	NMD transcript variant, Missense variant	1deleterious(0)	995probably damaging(0.994)	ENST00000444298
274	F/L	TMP_ESP_2_234905069	2:2349050692:234905069	C/G	0	SNP	ESP	ESP,	Missense variant	41deleterious(0.04)	316benign(0.315)	ENST00000456930
278	R/W	rs200066478	2:2348546322:234854632	C/T	0	SNP	dbSNP	0	Missense variant	1deleterious(0)	951probably damaging(0.95)	ENST00000324695
278	R/W	rs200066478	2:2348546322:234854632	C/T	0	SNP	dbSNP	0	NMD transcript variant, Missense variant	1deleterious(0)	980probably damaging(0.979)	ENST00000444298
280	A/T	rs145966390	2:2349050852:234905085	G/A	0	SNP	dbSNP	Multiple_observations,Frequency,ESP,	Missense variant	1deleterious(0)	756possibly damaging(0.755)	ENST00000456930
282	E/D	COSM1230578	2:2348546462:234854646	G/T	0	somatic_SNV	COSMIC	0	Missense variant	11deleterious(0.01)	417benign(0.416)	ENST00000324695
282	E/D	COSM1230578	2:2348546462:234854646	G/T	0	somatic_SNV	COSMIC	0	NMD transcript variant, Missense variant	11deleterious(0.01)	710possibly damaging(0.709)	ENST00000444298
284	M/L	rs200703853	2:2349050972:234905097	A/T	0	SNP	dbSNP	0	Missense variant	1001tolerated(1)	3benign(0.002)	ENST00000456930
284	M/I	TMP_ESP_2_234905099	2:2349050992:234905099	G/C	0	SNP	ESP	ESP,	Missense variant	41deleterious(0.04)	98benign(0.097)	ENST00000456930
288	R/C	rs141984026	2:2348546622:234854662	C/T	0	SNP	dbSNP	Multiple_observations,Frequency,ESP,	Missense variant	11deleterious(0.01)	729possibly damaging(0.728)	ENST00000324695
288	R/C	rs141984026	2:2348546622:234854662	C/T	0	SNP	dbSNP	Multiple_observations,Frequency,ESP,	NMD transcript variant, Missense variant	11deleterious(0.01)	717possibly damaging(0.716)	ENST00000444298
289	L/M	rs150720365	2:2348733232:234873323	C/A	0	SNP	dbSNP	ESP,	Missense variant	561tolerated(0.56)	1unknown(0)	ENST00000433712
289	L/R	rs199618852	2:2348733242:234873324	T/G	0	SNP	dbSNP	0	Missense variant	41deleterious(0.04)	1unknown(0)	ENST00000433712

292	D/E	TMP_ESP_2_234855733	2:2348557332:234855733	T/G	0	SNP	ESP	ESP,	Splice region variant, Missense variant	831tolerated(0.83)	8benign(0.007)	ENST00000324695
292	D/E	TMP_ESP_2_234855733	2:2348557332:234855733	T/G	0	SNP	ESP	ESP,	NMD transcript variant, Splice region variant, Missense variant	571tolerated(0.57)	77benign(0.076)	ENST00000444298
292	C/R	COSM1406479	2:2349051212:234905121	T/C	0	somatic_SNV	COSMIC	0	Missense variant	721tolerated(0.72)	47benign(0.046)	ENST00000456930
294	N/S	rs200467265	2:2348557382:234855738	A/G	0.001 (G)	SNP	dbSNP	0	Missense variant	31deleterious(0.03)	260benign(0.259)	ENST00000324695
294	N/S	rs200467265	2:2348557382:234855738	A/G	0.001 (G)	SNP	dbSNP	0	NMD transcript variant, Missense variant	221tolerated(0.22)	118benign(0.117)	ENST00000444298
294	C/S	rs201835578	2:2349051282:234905128	G/C	0	SNP	dbSNP	0	Missense variant	831tolerated(0.83)	47benign(0.046)	ENST00000456930
295	D/N	TMP_ESP_2_234873341	2:2348733412:234873341	G/A	0	SNP	ESP	ESP,	Missense variant	1deleterious(0)	1unknown(0)	ENST00000433712
298	N/S	TMP_ESP_2_234905140	2:2349051402:234905140	A/G	0	SNP	ESP	ESP,	Missense variant	751tolerated(0.75)	8benign(0.007)	ENST00000456930
299	A/V	rs201840075	2:2348733542:234873354	C/T	0	SNP	dbSNP	0	Missense variant	131tolerated(0.13)	1unknown(0)	ENST00000433712
304	C/F	rs201096876	2:2349051582:234905158	G/T	0	SNP	dbSNP	0	Splice region variant, Missense variant	711tolerated(0.71)	904possibly damaging(0.903)	ENST00000456930
311	T/A	rs200064222	2:2348733892:234873389	A/G	0	SNP	dbSNP	0	Missense variant	161tolerated(0.16)	1unknown(0)	ENST00000433712
312	R/P	rs201008719	2:2348733932:234873393	G/C	0	SNP	dbSNP	0	Missense variant	111tolerated(0.11)	1unknown(0)	ENST00000433712
312	R/Q	COSM1565677	2:2348733932:234873393	G/A	0	somatic_SNV	COSMIC	0	Missense variant	361tolerated(0.36)	1unknown(0)	ENST00000433712
314	K/N	rs202061896	2:2348557992:234855799	A/T	0	SNP	dbSNP	0	Splice region variant, Missense variant	1001tolerated(1)	988probably damaging(0.987)	ENST00000324695
314	K/N	rs202061896	2:2348557992:234855799	A/T	0	SNP	dbSNP	0	NMD transcript variant, Splice region variant, Missense variant	511tolerated(0.51)	998probably damaging(0.997)	ENST00000444298
314	L/V	rs201193454	2:2349155232:234915523	C/G	0	SNP	dbSNP	0	Missense variant	251tolerated(0.25)	1000probably damaging(0.999)	ENST00000456930
315	A/T	rs202108535	2:2349155262:234915526	G/A	0	SNP	dbSNP	0	Missense variant	1001tolerated(1)	281benign(0.28)	ENST00000456930
316	L/P	rs201627387	2:2348752572:234875257	T/C	0.001 (C)	SNP	dbSNP	0	Missense variant	1deleterious(0)	1unknown(0)	ENST00000433712
324	D/N	TMP_ESP_2_234875280	2:2348752802:234875280	G/A	0	SNP	ESP	ESP,	Missense variant	141tolerated(0.14)	1unknown(0)	ENST00000433712
324	Y/C	rs200883085	2:2349155542:234915554	A/G	0	SNP	dbSNP	0	Missense variant	1deleterious(0)	1001probably damaging(1)	ENST00000456930
327	L/S	TMP_ESP_2_234875290	2:2348752902:234875290	T/C	0	SNP	ESP	ESP,	Missense variant	21deleterious(0.02)	1unknown(0)	ENST00000433712
332	S/L	rs201141727	2:2348586452:234858645	C/T	0	SNP	dbSNP	0	Missense variant	1deleterious(0)	992probably damaging(0.991)	ENST00000324695

332	A/V	rs201836188	2:2349155782:234915578	C/T	0	SNP	dbSNP	ESP,	Missense variant	281tolerated(0.28)	286benign(0.285)	ENST00000456930
334	Y/C	COSM242076	2:2348753112:234875311	A/G	0	somatic_SNV	COSMIC	0	Missense variant	11deleterious(0.01)	1unknown(0)	ENST00000433712
335	S/F	rs200718215	2:2348753142:234875314	C/T	0.001 (T)	SNP	dbSNP	0	Missense variant	91tolerated(0.09)	1unknown(0)	ENST00000433712
336	A/T	rs200708894	2:2348586562:234858656	G/A	0	SNP	dbSNP	0	Missense variant	21deleterious(0.02)	997probably damaging(0.996)	ENST00000324695
336	C/Y	rs142251315	2:2348753172:234875317	G/A	0	SNP	dbSNP	Frequency,ESP,	Missense variant	21deleterious(0.02)	1unknown(0)	ENST00000433712
342	R/Q	rs139759512	2:2349167252:234916725	G/A	0	SNP	dbSNP	Multiple_observations,Frequency,ESP,	Missense variant	91tolerated(0.09)	39benign(0.038)	ENST00000456930
342	R/Q	COSM175165	2:2349167252:234916725	G/A	0	somatic_SNV	COSMIC	0	Missense variant	91tolerated(0.09)	39benign(0.038)	ENST00000456930
345	V/M	rs200856718	2:2348586832:234858683	G/A	0	SNP	dbSNP	0	Missense variant	101tolerated(0.1)	66benign(0.065)	ENST00000324695
348	A/T	rs201855344	2:2348586922:234858692	G/A	0	SNP	dbSNP	0	Missense variant	581tolerated(0.58)	1benign(0)	ENST00000324695
348	A/V	TMP_ESP_2_234875353	2:2348753532:234875353	C/T	0	SNP	ESP	ESP,	Missense variant	1deleterious(0)	16benign(0.015)	ENST00000433712
352	T/A	rs34709945	2:2348753642:234875364	A/G	0	SNP	dbSNP	Frequency,	Missense variant	181tolerated(0.18)	3benign(0.002)	ENST00000433712
353	D/Y	COSM383304	2:2348753672:234875367	G/T	0	somatic_SNV	COSMIC	0	Missense variant	31deleterious(0.03)	44benign(0.043)	ENST00000433712
354	Q/H	COSM342435	2:2348753722:234875372	G/T	0	somatic_SNV	COSMIC	0	Missense variant	81tolerated(0.08)	334benign(0.333)	ENST00000433712
354	N/K	rs199838351	2:2349259572:234925957	T/A	0	SNP	dbSNP	0	Missense variant	181tolerated(0.18)	2benign(0.001)	ENST00000456930
356	E/Q	rs145069600	2:2348587162:234858716	G/C	0	SNP	dbSNP	Multiple_observations,Frequency,ESP,	Missense variant	531tolerated(0.53)	358benign(0.357)	ENST00000324695
358	A/T	rs141040008	2:2348753822:234875382	G/T/A	0	SNP	dbSNP	Multiple_observations,Frequency,ESP,	Missense variant	11deleterious(0.01)	30benign(0.029)	ENST00000433712
358	A/S	rs141040008	2:2348753822:234875382	G/T/A	0	SNP	dbSNP	Multiple_observations,Frequency,ESP,	Missense variant	431tolerated(0.43)	19benign(0.018)	ENST00000433712
360	R/C	rs145944024	2:2348587282:234858728	C/T	0.001 (T)	SNP	dbSNP	Multiple_observations,Frequency,1000Genomes,ESP,	Missense variant	81tolerated(0.08)	525possibly damaging(0.524)	ENST00000324695
360	R/H	rs201962335	2:2348587292:234858729	G/A	0	SNP	dbSNP	ESP,	Missense variant	311tolerated(0.31)	3benign(0.002)	ENST00000324695
362	R/Q	rs201477771	2:2349259802:234925980	G/A	0	SNP	dbSNP	0	Missense variant	181tolerated(0.18)	615possibly damaging(0.614)	ENST00000456930
363	P/T	rs145572343	2:2348587372:234858737	C/A	0	SNP	dbSNP	Multiple_observations,Frequency,	Missense variant	571tolerated(0.57)	433benign(0.432)	ENST00000324695
364	R/C	TMP_ESP_2_234858740	2:2348587402:234858740	C/T	0	SNP	ESP	ESP,	Missense variant	41deleterious(0.04)	870possibly damaging(0.869)	ENST00000324695

364	R/C	COSM210452	2:2348587402:234858740	C/T	0	somatic_SNV	COSMIC	0	Missense variant	41deleterious(0.04)	870possibly damaging(0.869)	ENST00000324695
364	R/H	rs145044224	2:2348587412:234858741	G/A	0.002 (A)	SNP	dbSNP	Multiple_observations,Frequency,1000 Genomes,ESP,	Missense variant	91tolerated(0.09)	691possibly damaging(0.69)	ENST00000324695
365	T/M	rs202102033	2:2348587442:234858744	C/T	0	SNP	dbSNP	0	Missense variant	191tolerated(0.19)	844possibly damaging(0.843)	ENST00000324695
366	V/I	rs200414800	2:2348888382:234888838	G/A	0.001 (A)	SNP	dbSNP	ESP,	Missense variant	91tolerated(0.09)	144benign(0.143)	ENST00000433712
367	S/F	rs201051067	2:2348587502:234858750	C/T	0	SNP	dbSNP	0	Missense variant	11deleterious(0.01)	404benign(0.403)	ENST00000324695
367	S/F	COSM138349	2:2348587502:234858750	C/T	0	somatic_SNV	COSMIC	0	Missense variant	11deleterious(0.01)	404benign(0.403)	ENST00000324695
368	R/W	rs201761766	2:2348587522:234858752	C/T	0	SNP	dbSNP	0	Missense variant	21deleterious(0.02)	856possibly damaging(0.855)	ENST00000324695
368	R/Q	rs200303251	2:2348587532:234858753	G/A	0	SNP	dbSNP	0	Missense variant	611tolerated(0.61)	192benign(0.191)	ENST00000324695
369	V/A	rs200135938	2:2348888482:234888848	T/C	0	SNP	dbSNP	0	Missense variant	61tolerated(0.06)	38benign(0.037)	ENST00000433712
370	N/S	rs200970660	2:2348888512:234888851	A/G	0	SNP	dbSNP	0	Missense variant	1001tolerated(1)	85benign(0.084)	ENST00000433712
371	E/Q	COSM1482912	2:2348587612:234858761	G/C	0	somatic_SNV	COSMIC	0	Missense variant	101tolerated(0.1)	643possibly damaging(0.642)	ENST00000324695
372	W/C	rs200681075	2:2349260112:234926011	G/C	0	SNP	dbSNP	0	Missense variant	21deleterious(0.02)	3benign(0.002)	ENST00000456930
374	T/N	rs140502895	2:2348587712:234858771	C/T/A	0.001 (T)	SNP	dbSNP	Multiple_observations,Frequency,ESP,	Missense variant	1deleterious(0)	79benign(0.078)	ENST00000324695
374	T/I	rs140502895	2:2348587712:234858771	C/T/A	0.001 (T)	SNP	dbSNP	Multiple_observations,Frequency,ESP,	Missense variant	1001tolerated(1)	1benign(0)	ENST00000324695
375	G/D	rs190583531	2:2349260192:234926019	G/A	0.001 (A)	SNP	dbSNP	0	Missense variant	1deleterious(0)	8benign(0.007)	ENST00000456930
376	S/R	rs199509635	2:2348587782:234858778	T/A	0	SNP	dbSNP	0	Missense variant	171tolerated(0.17)	87benign(0.086)	ENST00000324695
381	T/M	rs200017552	2:2348888842:234888884	C/T	0	SNP	dbSNP	0	Missense variant	351tolerated(0.35)	979probably damaging(0.978)	ENST00000433712
387	C/R	rs145663793	2:2348625792:234862579	T/C	0	SNP	dbSNP	Multiple_observations,Frequency,ESP,	Missense variant	861tolerated(0.86)	2benign(0.001)	ENST00000324695
389	A/T	rs201621223	2:2348889072:234888907	G/A	0	SNP	dbSNP	0	Missense variant	361tolerated(0.36)	828possibly damaging(0.827)	ENST00000433712
393	F/I	rs200252660	2:2348889192:234888919	T/A	0	SNP	dbSNP	0	Missense variant	191tolerated(0.19)	809possibly damaging(0.808)	ENST00000433712
394	R/W	rs151304365	2:2348889222:234888922	C/T/A	0	SNP	dbSNP	ESP,	Splice region variant, Missense variant	1deleterious(0)	1001probably damaging(1)	ENST00000433712
394	R/W	COSM1018198	2:2348889222:234888922	C/T	0	somatic_SNV	COSMIC	0	Splice region variant, Missense variant	1deleterious(0)	1001probably damaging(1)	ENST00000433712

399	A/I	COSM97113	2:2348626152:234862615-234862616	GC/AT	0	somatic_substitution	COSMIC	0	Missense variant	21deleterious(0.02)	999probably damaging(0.998)	ENST00000324695
399	N/S	rs28902201	2:2348904322:234890432	A/G	0.005 (G)	SNP	dbSNP	Multiple_observations,Frequency,HapMap,1000Genomes,ESP,	Missense variant	721tolerated(0.72)	310benign(0.309)	ENST00000433712
408	V/A	rs201483334	2:2348904592:234890459	T/C	0	SNP	dbSNP	0	Missense variant	191tolerated(0.19)	995probably damaging(0.994)	ENST00000433712
413	Y/H	TMP_ESP_2_234862657	2:2348626572:234862657	T/C	0	SNP	ESP	ESP,	Missense variant	1deleterious(0)	993probably damaging(0.992)	ENST00000324695
419	S/N	rs7593557	2:2348637882:234863788	G/A	0.301 (A)	SNP	dbSNP	Multiple_observations,Frequency,HapMap,1000Genomes,ESP,	Missense variant	661tolerated(0.66)	1benign(0)	ENST00000324695
419	S/N	COSM149104	2:2348637882:234863788	G/A	0	somatic_SNV	COSMIC	0	Missense variant	661tolerated(0.66)	1benign(0)	ENST00000324695
422	D/E	rs200879817	2:2348637982:234863798	C/A	0	SNP	dbSNP	0	Missense variant	561tolerated(0.56)	2benign(0.001)	ENST00000324695
423	K/N	rs151229928	2:2348638012:234863801	G/C	0	SNP	dbSNP	ESP,	Missense variant	501tolerated(0.5)	341benign(0.34)	ENST00000324695
428	S/I	rs148696315	2:2348905192:234890519	G/T	0.001 (T)	SNP	dbSNP	1000Genomes,	Missense variant	21deleterious(0.02)	996probably damaging(0.995)	ENST00000433712
430	L/M	rs144120368	2:2348638202:234863820	C/A	0	SNP	dbSNP	Frequency,ESP,	Missense variant	51deleterious(0.05)	999probably damaging(0.998)	ENST00000324695
433	P/L	rs199828215	2:2348905342:234890534	C/T	0	SNP	dbSNP	ESP,	Missense variant	1deleterious(0)	1001probably damaging(1)	ENST00000433712
437	N/S	TMP_ESP_2_234863842	2:2348638422:234863842	A/G	0	SNP	ESP	ESP,	Missense variant	11deleterious(0.01)	985probably damaging(0.984)	ENST00000324695
439	L/P	rs140612583	2:2348638482:234863848	T/C	0.001 (C)	SNP	dbSNP	Multiple_observations,Frequency,	Missense variant	11deleterious(0.01)	1000probably damaging(0.999)	ENST00000324695
439	Q/L	rs111316052	2:2348905522:234890552	A/T	0	SNP	dbSNP	Frequency,	Missense variant	61tolerated(0.06)	910probably damaging(0.909)	ENST00000433712
441	M/I	COSM371853	2:2348905592:234890559	G/A	0	somatic_SNV	COSMIC	0	Splice region variant, Missense variant	221tolerated(0.22)	995probably damaging(0.994)	ENST00000433712
443	N/T	rs143385407	2:2348638602:234863860	A/C	0	SNP	dbSNP	Multiple_observations,Frequency,ESP,	Missense variant	91tolerated(0.09)	25benign(0.024)	ENST00000324695
451	R/S	rs182733234	2:2348638832:234863883	C/T/A	0.001 (T)	SNP	dbSNP	ESP,	Missense variant	421tolerated(0.42)	428benign(0.427)	ENST00000324695
451	R/C	rs182733234	2:2348638832:234863883	C/T/A	0.001 (T)	SNP	dbSNP	ESP,	Missense variant	181tolerated(0.18)	870possibly damaging(0.869)	ENST00000324695
452	R/Q	rs200547297	2:2348638872:234863887	G/A	0	SNP	dbSNP	0	Missense variant	1001tolerated(1)	280benign(0.279)	ENST00000324695
452	R/Q	COSM1406474	2:2348638872:234863887	G/A	0	somatic_SNV	COSMIC	0	Missense variant	1001tolerated(1)	280benign(0.279)	ENST00000324695
454	E/K	rs201971040	2:2348638922:234863892	G/A	0	SNP	dbSNP	0	Splice region variant, Missense variant	1001tolerated(1)	34benign(0.033)	ENST00000324695

455	W/R	TMP_ESP_2_234891736	2:2348917362:234891736	T/C	0	SNP	ESP	ESP,	Missense variant	1deleterious(0)	1001probably damaging(1)	ENST00000433712
457	V/A	rs201253609	2:2348917432:234891743	T/C	0	SNP	dbSNP	0	Missense variant	1deleterious(0)	997probably damaging(0.996)	ENST00000433712
459	Q/R	rs145289688	2:2348694332:234869433	A/G	0.001 (G)	SNP	dbSNP	1000Genomes,	Missense variant	21deleterious(0.02)	140benign(0.139)	ENST00000324695
462	M/T	rs28902173	2:2348694422:234869442	T/C	0.003 (C)	SNP	dbSNP	Multiple_observations,Frequency,HapMap,1000Genomes,ESP,	Missense variant	1deleterious(0)	952probably damaging(0.951)	ENST00000324695
464	T/M	rs143538093	2:2348694482:234869448	C/T	0	SNP	dbSNP	Multiple_observations,Frequency,ESP,	Missense variant	51deleterious(0.05)	569possibly damaging(0.568)	ENST00000324695
467	I/V	rs201369256	2:2348694562:234869456	A/G	0	SNP	dbSNP	0	Missense variant	731tolerated(0.73)	1benign(0)	ENST00000324695
471	P/S	TMP_ESP_2_234869468	2:2348694682:234869468	C/T	0	SNP	ESP	ESP,	Missense variant	1deleterious(0)	999probably damaging(0.998)	ENST00000324695
473	F/L	rs113858305	2:2348694742:234869474	T/C	0	SNP	dbSNP	Frequency,	Missense variant	11deleterious(0.01)	985probably damaging(0.984)	ENST00000324695
473	R/H	TMP_ESP_2_234891791	2:2348917912:234891791	G/A	0	SNP	ESP	ESP,	Missense variant	11deleterious(0.01)	1001probably damaging(1)	ENST00000433712
475	R/C	rs201940567	2:2348694802:234869480	C/T	0.001 (T)	SNP	dbSNP	0	Missense variant	1deleterious(0)	998probably damaging(0.997)	ENST00000324695
475	R/H	TMP_ESP_2_234869481	2:2348694812:234869481	G/A	0	SNP	ESP	ESP,	Missense variant	11deleterious(0.01)	998probably damaging(0.997)	ENST00000324695
479	R/C	rs201721376	2:2348918082:234891808	C/T	0	SNP	dbSNP	0	Missense variant	51deleterious(0.05)	1000probably damaging(0.999)	ENST00000433712
479	R/H	rs199784199	2:2348918092:234891809	G/A	0	SNP	dbSNP	0	Missense variant	11deleterious(0.01)	931probably damaging(0.93)	ENST00000433712
480	N/S	TMP_ESP_2_234869496	2:2348694962:234869496	A/G	0	SNP	ESP	ESP,	Missense variant	61tolerated(0.06)	985probably damaging(0.984)	ENST00000324695
480	S/L	COSM171795	2:2348918122:234891812	C/T	0	somatic_SNV	COSMIC	0	Missense variant	141tolerated(0.14)	965probably damaging(0.964)	ENST00000433712
481	G/D	COSM1494873	2:2348694992:234869499	G/A	0	somatic_SNV	COSMIC	0	Missense variant	1deleterious(0)	999probably damaging(0.998)	ENST00000324695
485	R/W	rs111972494	2:2348695102:234869510	C/T	0	SNP	dbSNP	Multiple_observations,Frequency,ESP,	Missense variant	11deleterious(0.01)	482possibly damaging(0.481)	ENST00000324695
485	R/Q	rs200772894	2:2348695112:234869511	G/A	0.001 (A)	SNP	dbSNP	0	Missense variant	201tolerated(0.2)	3benign(0.002)	ENST00000324695
489	T/I	rs76512385	2:2348695232:234869523	C/T	0.001 (T)	SNP	dbSNP	Multiple_observations,Frequency,1000Genomes,ESP,	Missense variant	11deleterious(0.01)	571possibly damaging(0.57)	ENST00000324695
491	D/G	rs199871046	2:2348695292:234869529	A/G	0	SNP	dbSNP	0	Missense variant	201tolerated(0.2)	74benign(0.073)	ENST00000324695
492	V/F	rs200514662	2:2348695312:234869531	G/T	0	SNP	dbSNP	0	Missense variant	41deleterious(0.04)	665possibly damaging(0.664)	ENST00000324695

493	L/F	rs201464095	2:2348695342:234869534	C/T	0	SNP	dbSNP	0	Missense variant	1deleterious(0)	999probably damaging(0.998)	ENST00000324695
493	V/M	rs149328116	2:2348918502:234891850	G/A	0.001 (A)	SNP	dbSNP	Multiple_observations,Frequency,ESP,	Missense variant	21deleterious(0.02)	369benign(0.368)	ENST00000433712
494	T/I	rs200065004	2:2348695382:234869538	C/T	0	SNP	dbSNP	0	Missense variant	501tolerated(0.5)	59benign(0.058)	ENST00000324695
497	F/L	rs200953031	2:2348695462:234869546	T/C	0	SNP	dbSNP	0	Missense variant	11deleterious(0.01)	88benign(0.087)	ENST00000324695
497	V/M	rs199993016	2:2348918622:234891862	G/A	0	SNP	dbSNP	0	Missense variant	121tolerated(0.12)	996probably damaging(0.995)	ENST00000433712
497	V/M	COSM210455	2:2348918622:234891862	G/A	0	somatic_SNV	COSMIC	0	Missense variant	121tolerated(0.12)	996probably damaging(0.995)	ENST00000433712
499	G/S	rs201072281	2:2348918682:234891868	G/A	0	SNP	dbSNP	0	Splice region variant, Missense variant	591tolerated(0.59)	754possibly damaging(0.753)	ENST00000433712
500	H/Y	TMP_ESP_2_234869555	2:2348695552:234869555	C/T	0	SNP	ESP	ESP,	Missense variant	161tolerated(0.16)	244benign(0.243)	ENST00000324695
501	T/M	rs200355253	2:2348943382:234894338	C/T	0	SNP	dbSNP	0	Missense variant	111tolerated(0.11)	979probably damaging(0.978)	ENST00000433712
503	T/M	rs149254613	2:2348695652:234869565	C/T	0	SNP	dbSNP	Multiple_observations,Frequency,ESP,	Missense variant	31deleterious(0.03)	688possibly damaging(0.687)	ENST00000324695
505	A/V	rs201934673	2:2348943502:234894350	C/T	0	SNP	dbSNP	0	Missense variant	281tolerated(0.28)	724possibly damaging(0.723)	ENST00000433712
507	R/W	COSM1406476	2:2348695762:234869576	C/T	0	somatic_SNV	COSMIC	0	Missense variant	51deleterious(0.05)	709possibly damaging(0.708)	ENST00000324695
507	R/Q	rs201095192	2:2348695772:234869577	G/A	0	SNP	dbSNP	0	Missense variant	341tolerated(0.34)	49benign(0.048)	ENST00000324695
512	A/T	rs200884995	2:2348695912:234869591	G/A	0	SNP	dbSNP	0	Missense variant	241tolerated(0.24)	855possibly damaging(0.854)	ENST00000324695
512	A/T	COSM210453	2:2348695912:234869591	G/A	0	somatic_SNV	COSMIC	0	Missense variant	241tolerated(0.24)	855possibly damaging(0.854)	ENST00000324695
513	K/N	COSM277842	2:2348695962:234869596	G/T	0	somatic_SNV	COSMIC	0	Missense variant	241tolerated(0.24)	410benign(0.409)	ENST00000324695
517	L/M	rs139760142	2:2348943852:234894385	C/A	0.001 (A)	SNP	dbSNP	Multiple_observations,1000Genomes,	Missense variant	221tolerated(0.22)	717possibly damaging(0.716)	ENST00000433712
522	T/M	COSM1018191	2:2348696222:234869622	C/T	0	somatic_SNV	COSMIC	0	Missense variant	111tolerated(0.11)	998probably damaging(0.997)	ENST00000324695
524	H/R	rs199727864	2:2348944072:234894407	A/G	0	SNP	dbSNP	0	Missense variant	531tolerated(0.53)	118benign(0.117)	ENST00000433712
527	P/S	rs200680058	2:2348944152:234894415	C/T	0	SNP	dbSNP	0	Missense variant	131tolerated(0.13)	945probably damaging(0.944)	ENST00000433712
528	R/W	rs147248663	2:2348944182:234894418	C/T	0	SNP	dbSNP	Multiple_observations,Frequency,ESP,	Missense variant	41deleterious(0.04)	975probably damaging(0.974)	ENST00000433712
528	R/Q	rs199616111	2:2348944192:234894419	G/T/A	0	SNP	dbSNP	0	Missense variant	371tolerated(0.37)	921probably damaging(0.92)	ENST00000433712
528	R/L	rs199616111	2:2348944192:234894419	G/T/A	0	SNP	dbSNP	0	Missense variant	361tolerated(0.36)	199benign(0.198)	ENST00000433712

530	N/K	COSM1230576	2:2348696472:234869647	C/A	0	somatic_SNV	COSMIC	0	Missense variant	701tolerated(0.7)	8benign(0.007)	ENST00000324695
530	P/S	rs200606227	2:2348944242:234894424	C/T	0	SNP	dbSNP	0	Missense variant	31deleterious(0.03)	999probably damaging(0.998)	ENST00000433712
531	E/K	rs202035579	2:2348944272:234894427	G/A	0	SNP	dbSNP	0	Missense variant	171tolerated(0.17)	981probably damaging(0.98)	ENST00000433712
532	R/Q	rs201941621	2:2348696522:234869652	G/A	0	SNP	dbSNP	ESP,	Missense variant	651tolerated(0.65)	3benign(0.002)	ENST00000324695
536	R/W	rs201447068	2:2348696632:234869663	C/T	0	SNP	dbSNP	0	Missense variant	171tolerated(0.17)	1benign(0)	ENST00000324695
536	R/Q	rs199756522	2:2348696642:234869664	G/A	0	SNP	dbSNP	ESP,	Missense variant	331tolerated(0.33)	14benign(0.013)	ENST00000324695
537	K/N	COSM108177	2:2348696682:234869668	G/T	0	somatic_SNV	COSMIC	0	Missense variant	191tolerated(0.19)	5benign(0.004)	ENST00000324695
537	K/N	rs149963916	2:2348696682:234869668	G/T	0	somatic_SNV	dbSNP	0	Missense variant	191tolerated(0.19)	5benign(0.004)	ENST00000324695
543	R/W	rs200707338	2:2348696842:234869684	C/T	0	SNP	dbSNP	0	Missense variant	61tolerated(0.06)	613possibly damaging(0.612)	ENST00000324695
543	R/W	COSM1565678	2:2348696842:234869684	C/T	0	somatic_SNV	COSMIC	0	Missense variant	61tolerated(0.06)	613possibly damaging(0.612)	ENST00000324695
543	R/Q	rs201047162	2:2348696852:234869685	G/A	0	SNP	dbSNP	0	Missense variant	501tolerated(0.5)	21benign(0.02)	ENST00000324695
544	D/E	rs142882642	2:2348696892:234869689	C/G/T	0	SNP	dbSNP	Multiple_observations,Frequency,ESP,	Missense variant	351tolerated(0.35)	2benign(0.001)	ENST00000324695
545	E/K	rs200725140	2:2348696902:234869690	G/A	0	SNP	dbSNP	0	Missense variant	861tolerated(0.86)	13benign(0.012)	ENST00000324695
548	I/V	rs200180972	2:2348696992:234869699	A/G	0	SNP	dbSNP	0	Missense variant	461tolerated(0.46)	2benign(0.001)	ENST00000324695
549	E/K	rs200749841	2:2348697022:234869702	G/A	0	SNP	dbSNP	0	Missense variant	741tolerated(0.74)	20benign(0.019)	ENST00000324695
558	R/W	rs202225959	2:2348719442:234871944	C/T	0	SNP	dbSNP	0	Missense variant	61tolerated(0.06)	850possibly damaging(0.849)	ENST00000324695
558	R/Q	rs201115161	2:2348719452:234871945	G/A/T	0.001 (A)	SNP	dbSNP	ESP,	Missense variant	581tolerated(0.58)	105benign(0.104)	ENST00000324695
558	R/L	rs201115161	2:2348719452:234871945	G/A/T	0.001 (A)	SNP	dbSNP	ESP,	Missense variant	391tolerated(0.39)	224benign(0.223)	ENST00000324695
558	R/Q	COSM1482913	2:2348719452:234871945	G/A	0	somatic_SNV	COSMIC	0	Missense variant	581tolerated(0.58)	105benign(0.104)	ENST00000324695
560	P/S	rs141865492	2:2348719502:234871950	C/T	0	SNP	dbSNP	Frequency,ESP,	Missense variant	11deleterious(0.01)	999probably damaging(0.998)	ENST00000324695
560	T/M	rs202017617	2:2349049752:234904975	C/T	0	SNP	dbSNP	0	Missense variant	1deleterious(0)	1001probably damaging(1)	ENST00000433712
561	V/M	TMP_ESP_2_234904977	2:2349049772:234904977	G/A	0	SNP	ESP	ESP,	Missense variant	1deleterious(0)	972probably damaging(0.971)	ENST00000433712
562	Q/E	COSM1531359	2:2348719562:234871956	C/G	0	somatic_SNV	COSMIC	0	Missense variant	131tolerated(0.13)	150benign(0.149)	ENST00000324695

564	V/I	rs148556306	2:2349049862:234904986	G/A	0	SNP	dbSNP	Multiple_observations,Frequency,ESP,	Missense variant	1deleterious(0)	998probably damaging(0.997)	ENST00000433712
565	Q/R	rs200248747	2:2349049902:234904990	A/G	0	SNP	dbSNP	0	Missense variant	1deleterious(0)	992probably damaging(0.991)	ENST00000433712
566	I/M	TMP_ESP_2_234871970	2:2348719702:234871970	C/G	0	SNP	ESP	ESP,	Missense variant	11deleterious(0.01)	685possibly damaging(0.684)	ENST00000324695
570	Q/K	rs143128851	2:2349050042:234905004	C/A	0	SNP	dbSNP	Frequency,ESP,	Missense variant	1001tolerated(1)	453possibly damaging(0.452)	ENST00000433712
571	Q/R	rs201451492	2:2348719842:234871984	A/G	0	SNP	dbSNP	0	Missense variant	1deleterious(0)	972probably damaging(0.971)	ENST00000324695
572	N/T	rs201960730	2:2348719872:234871987	A/C	0.001 (C)	SNP	dbSNP	0	Missense variant	1deleterious(0)	992probably damaging(0.991)	ENST00000324695
573	K/N	COSM335618	2:2349050152:234905015	G/T	0	somatic_SNV	COSMIC	0	Missense variant	1deleterious(0)	1000probably damaging(0.999)	ENST00000433712
577	S/F	rs149198571	2:2348720022:234872002	C/T	0	SNP	dbSNP	Frequency,ESP,	Missense variant	1deleterious(0)	998probably damaging(0.997)	ENST00000324695
579	V/I	rs201927659	2:2348720072:234872007	G/A	0.001 (A)	SNP	dbSNP	0	Missense variant	1001tolerated(1)	882possibly damaging(0.881)	ENST00000324695
579	L/P	TMP_ESP_2_234905032	2:2349050322:234905032	T/C	0	SNP	ESP	ESP,	Missense variant	1deleterious(0)	1001probably damaging(1)	ENST00000433712
585	R/K	rs148231346	2:2348732762:234873276	G/A	0.001 (A)	SNP	dbSNP	Multiple_observations,Frequency,ESP,	Missense variant	771tolerated(0.77)	2benign(0.001)	ENST00000324695
586	R/C	rs199727860	2:2349050522:234905052	C/T	0	SNP	dbSNP	0	Missense variant	1deleterious(0)	999probably damaging(0.998)	ENST00000433712
586	R/H	COSM1406478	2:2349050532:234905053	G/A	0	somatic_SNV	COSMIC	0	Missense variant	1deleterious(0)	997probably damaging(0.996)	ENST00000433712
591	F/L	TMP_ESP_2_234905069	2:2349050692:234905069	C/G	0	SNP	ESP	ESP,	Missense variant	61tolerated(0.06)	453possibly damaging(0.452)	ENST00000433712
597	A/T	rs145966390	2:2349050852:234905085	G/A	0	SNP	dbSNP	Multiple_observations,Frequency,ESP,	Missense variant	21deleterious(0.02)	737possibly damaging(0.736)	ENST00000433712
601	L/M	rs150720365	2:2348733232:234873323	C/A	0	SNP	dbSNP	ESP,	Missense variant	281tolerated(0.28)	84benign(0.083)	ENST00000324695
601	L/R	rs199618852	2:2348733242:234873324	T/G	0	SNP	dbSNP	0	Missense variant	21deleterious(0.02)	18benign(0.017)	ENST00000324695
601	M/L	rs200703853	2:2349050972:234905097	A/T	0	SNP	dbSNP	0	Missense variant	1001tolerated(1)	8benign(0.007)	ENST00000433712
601	M/I	TMP_ESP_2_234905099	2:2349050992:234905099	G/C	0	SNP	ESP	ESP,	Missense variant	121tolerated(0.12)	133benign(0.132)	ENST00000433712
607	D/N	TMP_ESP_2_234873341	2:2348733412:234873341	G/A	0	SNP	ESP	ESP,	Missense variant	11deleterious(0.01)	373benign(0.372)	ENST00000324695
609	C/R	COSM1406479	2:2349051212:234905121	T/C	0	somatic_SNV	COSMIC	0	Missense variant	821tolerated(0.82)	33benign(0.032)	ENST00000433712
611	A/V	rs201840075	2:2348733542:234873354	C/T	0	SNP	dbSNP	0	Missense variant	41deleterious(0.04)	245benign(0.244)	ENST00000324695
611	C/S	rs201835578	2:2349051282:234905128	G/C	0	SNP	dbSNP	0	Missense variant	801tolerated(0.8)	33benign(0.032)	ENST00000433712

615	N/S	TMP_ESP_2_234905140	2:2349051402:234905140	A/G	0	SNP	ESP	ESP,	Missense variant	141tolerated(0.14)	8benign(0.007)	ENST00000433712
621	C/F	rs201096876	2:2349051582:234905158	G/T	0	SNP	dbSNP	0	Splice region variant, Missense variant	31deleterious(0.03)	733possibly damaging(0.732)	ENST00000433712
623	T/A	rs200064222	2:2348733892:234873389	A/G	0	SNP	dbSNP	0	Missense variant	751tolerated(0.75)	167benign(0.166)	ENST00000324695
624	R/P	rs201008719	2:2348733932:234873393	G/C	0	SNP	dbSNP	0	Missense variant	61tolerated(0.06)	690possibly damaging(0.689)	ENST00000324695
624	R/Q	COSM1565677	2:2348733932:234873393	G/A	0	somatic_SNV	COSMIC	0	Missense variant	221tolerated(0.22)	15benign(0.014)	ENST00000324695
628	L/P	rs201627387	2:2348752572:234875257	T/C	0.001 (C)	SNP	dbSNP	0	Missense variant	1deleterious(0)	998probably damaging(0.997)	ENST00000324695
631	L/V	rs201193454	2:2349155232:234915523	C/G	0	SNP	dbSNP	0	Missense variant	161tolerated(0.16)	999probably damaging(0.998)	ENST00000433712
632	A/T	rs202108535	2:2349155262:234915526	G/A	0	SNP	dbSNP	0	Missense variant	971tolerated(0.97)	357benign(0.356)	ENST00000433712
636	D/N	TMP_ESP_2_234875280	2:2348752802:234875280	G/A	0	SNP	ESP	ESP,	Missense variant	41deleterious(0.04)	57benign(0.056)	ENST00000324695
639	L/S	TMP_ESP_2_234875290	2:2348752902:234875290	T/C	0	SNP	ESP	ESP,	Missense variant	21deleterious(0.02)	749possibly damaging(0.748)	ENST00000324695
641	Y/C	rs200883085	2:2349155542:234915554	A/G	0	SNP	dbSNP	0	Missense variant	1deleterious(0)	1001probably damaging(1)	ENST00000433712
646	Y/C	COSM242076	2:2348753112:234875311	A/G	0	somatic_SNV	COSMIC	0	Missense variant	41deleterious(0.04)	4benign(0.003)	ENST00000324695
647	S/F	rs200718215	2:2348753142:234875314	C/T	0.001 (T)	SNP	dbSNP	0	Missense variant	91tolerated(0.09)	325benign(0.324)	ENST00000324695
648	C/Y	rs142251315	2:2348753172:234875317	G/A	0	SNP	dbSNP	Frequency,ESP,	Missense variant	31deleterious(0.03)	325benign(0.324)	ENST00000324695
649	A/V	rs201836188	2:2349155782:234915578	C/T	0	SNP	dbSNP	ESP,	Missense variant	71tolerated(0.07)	464possibly damaging(0.463)	ENST00000433712
659	R/Q	rs139759512	2:2349167252:234916725	G/A	0	SNP	dbSNP	Multiple_observations,Frequency,ESP,	Missense variant	31deleterious(0.03)	230benign(0.229)	ENST00000433712
659	R/Q	COSM175165	2:2349167252:234916725	G/A	0	somatic_SNV	COSMIC	0	Missense variant	31deleterious(0.03)	230benign(0.229)	ENST00000433712
660	A/V	TMP_ESP_2_234875353	2:2348753532:234875353	C/T	0	SNP	ESP	ESP,	Missense variant	1deleterious(0)	218benign(0.217)	ENST00000324695
664	T/A	rs34709945	2:2348753642:234875364	A/G	0	SNP	dbSNP	Frequency,	Missense variant	261tolerated(0.26)	20benign(0.019)	ENST00000324695
665	D/Y	COSM383304	2:2348753672:234875367	G/T	0	somatic_SNV	COSMIC	0	Missense variant	11deleterious(0.01)	583possibly damaging(0.582)	ENST00000324695
666	Q/H	COSM342435	2:2348753722:234875372	G/T	0	somatic_SNV	COSMIC	0	Missense variant	111tolerated(0.11)	416benign(0.415)	ENST00000324695
667	L/V	rs201188178	2:2349232062:234923206	C/G	0	SNP	dbSNP	0	Splice region variant, Missense variant	1001tolerated(1)	26benign(0.025)	ENST00000433712
670	A/T	rs141040008	2:2348753822:234875382	G/T/A	0	SNP	dbSNP	Multiple_observations,Frequency,ESP,	Missense variant	11deleterious(0.01)	238benign(0.237)	ENST00000324695

670	A/S	rs141040008	2:2348753822:234875382	G/T/A	0	SNP	dbSNP	Multiple_observations,Frequency,ESP,	Missense variant	601tolerated(0.6)	69benign(0.068)	ENST00000324695
679	N/S	rs144357618	2:2349232432:234923243	A/G	0	SNP	dbSNP	Multiple_observations,Frequency,ESP,	Missense variant	531tolerated(0.53)	6benign(0.005)	ENST00000433712
682	K/E	rs201564611	2:2349232512:234923251	A/G	0	SNP	dbSNP	0	Missense variant	1deleterious(0)	23benign(0.022)	ENST00000433712
701	I/S	rs200853140	2:2348784152:234878415	T/G	0	SNP	dbSNP	0	Missense variant	31deleterious(0.03)	77benign(0.076)	ENST00000324695
713	R/M	rs201540847	2:2348784512:234878451	G/T	0	SNP	dbSNP	0	Splice region variant, Missense variant	1deleterious(0)	995probably damaging(0.994)	ENST00000324695
714	K/R	rs202110003	2:2348788562:234878856	A/G	0	SNP	dbSNP	0	Splice region variant, Missense variant	321tolerated(0.32)	8benign(0.007	ENST00000324695
716	P/L	COSM228908	2:2348788622:234878862	C/T	0	somatic_SNV	COSMIC	0	Missense variant	91tolerated(0.09)	62benign(0.061)	ENST00000324695
718	D/N	rs201564419	2:2348788672:234878867	G/A	0	SNP	dbSNP	0	Missense variant	581tolerated(0.58)	4benign(0.003)	ENST00000324695
718	D/N	COSM1230579	2:2348788672:234878867	G/A	0	somatic_SNV	COSMIC	0	Missense variant	581tolerated(0.58)	4benign(0.003)	ENST00000324695
720	H/L	rs145489801	2:2348788742:234878874	A/T	0	SNP	dbSNP	Frequency,	Missense variant	781tolerated(0.78)	12benign(0.011)	ENST00000324695
728	V/A	rs199676608	2:2348788982:234878898	T/C	0	SNP	dbSNP	0	Missense variant	671tolerated(0.67)	2benign(0.001)	ENST00000324695
732	T/I	rs17862932	2:2348789102:234878910	C/T	0.001 (T)	SNP	dbSNP	Multiple_observations,Frequency,HapMap,ESP,	Missense variant	31deleterious(0.03)	995probably damaging(0.994)	ENST00000324695
734	P/S	rs200582935	2:2348789152:234878915	C/T	0	SNP	dbSNP	0	Missense variant	1deleterious(0)	998probably damaging(0.997)	ENST00000324695
736	V/M	rs200217624	2:2348789212:234878921	G/A	0	SNP	dbSNP	ESP,	Missense variant	1deleterious(0)	913probably damaging(0.912)	ENST00000324695
736	V/M	COSM1018196	2:2348789212:234878921	G/A	0	somatic_SNV	COSMIC	0	Missense variant	1deleterious(0)	913probably damaging(0.912)	ENST00000324695
739	S/P	TMP_ESP_2_234878930	2:2348789302:234878930	T/C	0	SNP	ESP	ESP,	Missense variant	181tolerated(0.18)	997probably damaging(0.996)	ENST00000324695
739	S/F	COSM442530	2:2348789312:234878931	C/T	0	somatic_SNV	COSMIC	0	Missense variant	661tolerated(0.66)	998probably damaging(0.997)	ENST00000324695
740	W/L	COSM1531358	2:2348789342:234878934	G/T	0	somatic_SNV	COSMIC	0	Missense variant	1001tolerated(1)	992probably damaging(0.991)	ENST00000324695
747	A/T	rs200649506	2:2348789542:234878954	G/A	0	SNP	dbSNP	ESP,	Missense variant	191tolerated(0.19)	105benign(0.104)	ENST00000324695
747	A/T	COSM271578	2:2348789542:234878954	G/A	0	somatic_SNV	COSMIC	0	Missense variant	191tolerated(0.19)	105benign(0.104)	ENST00000324695
748	F/L	COSM165188	2:2348789592:234878959	C/G	0	somatic_SNV	COSMIC	0	Missense variant	1deleterious(0)	985probably damaging(0.984)	ENST00000324695
749	L/F	COSM327584	2:2348789602:234878960	C/T	0	somatic_SNV	COSMIC	0	Missense variant	1deleterious(0)	999probably damaging(0.998)	ENST00000324695

755	V/M	rs199991435	2:2348789782:234878978	G/A	0	SNP	dbSNP	0	Missense variant	1deleterious(0)	998probably damaging(0.997)	ENST00000324695
757	L/F	TMP_ESP_2_234878984	2:2348789842:234878984	C/T	0	SNP	ESP	ESP,	Missense variant	11deleterious(0.01)	999probably damaging(0.998)	ENST00000324695
761	H/R	rs149382347	2:2348789972:234878997	A/G	0	SNP	dbSNP	Multiple_observations,Frequency,ESP,	Missense variant	131tolerated(0.13)	31benign(0.03)	ENST00000324695
762	S/L	rs201939575	2:2348790002:234879000	C/T	0	SNP	dbSNP	0	Missense variant	241tolerated(0.24)	72benign(0.071)	ENST00000324695
766	P/T	rs201080817	2:2348790112:234879011	C/A	0.001 (A)	SNP	dbSNP	0	Missense variant	741tolerated(0.74)	1benign(0)	ENST00000324695
767	P/T	rs202105112	2:2348790142:234879014	C/A	0	SNP	dbSNP	0	Missense variant	681tolerated(0.68)	175benign(0.174)	ENST00000324695
767	P/R	rs148846269	2:2348790152:234879015	C/G	0.001 (G)	SNP	dbSNP	Multiple_observations,1000Genomes,	Missense variant	611tolerated(0.61)	447possibly damaging(0.446)	ENST00000324695
769	L/M	rs200982995	2:2348790202:234879020	C/A	0	SNP	dbSNP	0	Missense variant	171tolerated(0.17)	880possibly damaging(0.879)	ENST00000324695
773	S/L	rs200296802	2:2348790332:234879033	C/T	0	SNP	dbSNP	0	Missense variant	541tolerated(0.54)	1benign(0)	ENST00000324695
777	V/I	rs199516053	2:2348790442:234879044	G/A	0	SNP	dbSNP	0	Missense variant	551tolerated(0.55)	10benign(0.009)	ENST00000324695
788	V/I	rs200414800	2:2348888382:234888838	G/A	0.001 (A)	SNP	dbSNP	ESP,	Missense variant	401tolerated(0.4)	1benign(0)	ENST00000324695
791	V/A	rs200135938	2:2348888482:234888848	T/C	0	SNP	dbSNP	0	Missense variant	781tolerated(0.78)	2benign(0.001)	ENST00000324695
792	N/S	rs200970660	2:2348888512:234888851	A/G	0	SNP	dbSNP	0	Missense variant	731tolerated(0.73)	12benign(0.011)	ENST00000324695
803	T/M	rs200017552	2:2348888842:234888884	C/T	0	SNP	dbSNP	0	Missense variant	121tolerated(0.12)	708possibly damaging(0.707)	ENST00000324695
811	A/T	rs201621223	2:2348889072:234888907	G/A	0	SNP	dbSNP	0	Missense variant	251tolerated(0.25)	784possibly damaging(0.783)	ENST00000324695
815	F/I	rs200252660	2:2348889192:234888919	T/A	0	SNP	dbSNP	0	Missense variant	91tolerated(0.09)	267benign(0.266)	ENST00000324695
816	R/W	rs151304365	2:2348889222:234888922	C/T/A	0	SNP	dbSNP	ESP,	Splice region variant, Missense variant	1deleterious(0)	970probably damaging(0.969)	ENST00000324695
816	R/W	COSM1018198	2:2348889222:234888922	C/T	0	somatic_SNV	COSMIC	0	Splice region variant, Missense variant	1deleterious(0)	970probably damaging(0.969)	ENST00000324695
821	N/S	rs28902201	2:2348904322:234890432	A/G	0.005 (G)	SNP	dbSNP	Multiple_observations,Frequency,HapMap,1000Genomes,ESP,	Missense variant	731tolerated(0.73)	36benign(0.035)	ENST00000324695
830	V/A	rs201483334	2:2348904592:234890459	T/C	0	SNP	dbSNP	0	Missense variant	111tolerated(0.11)	995probably damaging(0.994)	ENST00000324695
850	S/I	rs148696315	2:2348905192:234890519	G/T	0.001 (T)	SNP	dbSNP	1000Genomes,	Missense variant	1deleterious(0)	381benign(0.38)	ENST00000324695
855	P/L	rs199828215	2:2348905342:234890534	C/T	0	SNP	dbSNP	ESP,	Missense variant	1deleterious(0)	1000probably damaging(0.999)	ENST00000324695

861	Q/L	rs111316052	2:2348905522:234890552	A/T	0	SNP	dbSNP	Frequency,	Missense variant	31deleterious(0.03)	993probably damaging(0.992)	ENST00000324695
863	M/I	COSM371853	2:2348905592:234890559	G/A	0	somatic_SNV	COSMIC	0	Splice region variant, Missense variant	1deleterious(0)	972probably damaging(0.971)	ENST00000324695
877	W/R	TMP_ESP_2_234891736	2:2348917362:234891736	T/C	0	SNP	ESP	ESP,	Missense variant	1deleterious(0)	1000probably damaging(0.999)	ENST00000324695
879	V/A	rs201253609	2:2348917432:234891743	T/C	0	SNP	dbSNP	0	Missense variant	1deleterious(0)	441possibly damaging(0.44)	ENST00000324695
895	R/H	TMP_ESP_2_234891791	2:2348917912:234891791	G/A	0	SNP	ESP	ESP,	Missense variant	1deleterious(0)	999probably damaging(0.998)	ENST00000324695
901	R/C	rs201721376	2:2348918082:234891808	C/T	0	SNP	dbSNP	0	Missense variant	1deleterious(0)	1000probably damaging(0.999)	ENST00000324695
901	R/H	rs199784199	2:2348918092:234891809	G/A	0	SNP	dbSNP	0	Missense variant	21deleterious(0.02)	999probably damaging(0.998)	ENST00000324695
902	S/L	COSM171795	2:2348918122:234891812	C/T	0	somatic_SNV	COSMIC	0	Missense variant	21deleterious(0.02)	997probably damaging(0.996)	ENST00000324695
915	V/M	rs149328116	2:2348918502:234891850	G/A	0.001 (A)	SNP	dbSNP	Multiple_observations,Frequency,ESP,	Missense variant	101tolerated(0.1)	656possibly damaging(0.655)	ENST00000324695
919	V/M	rs199993016	2:2348918622:234891862	G/A	0	SNP	dbSNP	0	Missense variant	91tolerated(0.09)	886possibly damaging(0.885)	ENST00000324695
919	V/M	COSM210455	2:2348918622:234891862	G/A	0	somatic_SNV	COSMIC	0	Missense variant	91tolerated(0.09)	886possibly damaging(0.885)	ENST00000324695
921	G/S	rs201072281	2:2348918682:234891868	G/A	0	SNP	dbSNP	0	Splice region variant, Missense variant	161tolerated(0.16)	18benign(0.017)	ENST00000324695
923	T/M	rs200355253	2:2348943382:234894338	C/T	0	SNP	dbSNP	0	Missense variant	91tolerated(0.09)	936probably damaging(0.935)	ENST00000324695
927	A/V	rs201934673	2:2348943502:234894350	C/T	0	SNP	dbSNP	0	Missense variant	131tolerated(0.13)	44benign(0.043)	ENST00000324695
939	L/M	rs139760142	2:2348943852:234894385	C/A	0.001 (A)	SNP	dbSNP	Multiple_observations,1000Genomes,	Missense variant	231tolerated(0.23)	249benign(0.248)	ENST00000324695
946	H/R	rs199727864	2:2348944072:234894407	A/G	0	SNP	dbSNP	0	Missense variant	601tolerated(0.6)	26benign(0.025)	ENST00000324695
949	P/S	rs200680058	2:2348944152:234894415	C/T	0	SNP	dbSNP	0	Missense variant	71tolerated(0.07)	1000probably damaging(0.999)	ENST00000324695
950	R/W	rs147248663	2:2348944182:234894418	C/T	0	SNP	dbSNP	Multiple_observations,Frequency,ESP,	Missense variant	11deleterious(0.01)	1000probably damaging(0.999)	ENST00000324695
950	R/Q	rs199616111	2:2348944192:234894419	G/T/A	0	SNP	dbSNP	0	Missense variant	51tolerated(0.05)	997probably damaging(0.996)	ENST00000324695
950	R/L	rs199616111	2:2348944192:234894419	G/T/A	0	SNP	dbSNP	0	Missense variant	311tolerated(0.31)	998probably damaging(0.997)	ENST00000324695
952	P/S	rs200606227	2:2348944242:234894424	C/T	0	SNP	dbSNP	0	Missense variant	11deleterious(0.01)	1000probably damaging(0.999)	ENST00000324695
953	E/K	rs202035579	2:2348944272:234894427	G/A	0	SNP	dbSNP	0	Missense variant	71tolerated(0.07)	285benign(0.284)	ENST00000324695
982	T/M	rs202017617	2:2349049752:234904975	C/T	0	SNP	dbSNP	0	Missense variant	1deleterious(0)	998probably damaging(0.997)	ENST00000324695

983	V/M	TMP_ESP_2_234904977	2:2349049772:234904977	G/A	0	SNP	ESP	ESP,	Missense variant	11deleterious(0.01)	997probably damaging(0.996)	ENST00000324695
986	V/I	rs148556306	2:2349049862:234904986	G/A	0	SNP	dbSNP	Multiple_observations,Frequency,ESP,	Missense variant	91tolerated(0.09)	977probably damaging(0.976)	ENST00000324695
987	Q/R	rs200248747	2:2349049902:234904990	A/G	0	SNP	dbSNP	0	Missense variant	1deleterious(0)	970probably damaging(0.969)	ENST00000324695
992	Q/K	rs143128851	2:2349050042:234905004	C/A	0	SNP	dbSNP	Frequency,ESP,	Missense variant	621tolerated(0.62)	951probably damaging(0.95)	ENST00000324695
995	K/N	COSM335618	2:2349050152:234905015	G/T	0	somatic_SNV	COSMIC	0	Missense variant	1deleterious(0)	992probably damaging(0.991)	ENST00000324695
1001	L/P	TMP_ESP_2_234905032	2:2349050322:234905032	T/C	0	SNP	ESP	ESP,	Missense variant	1deleterious(0)	998probably damaging(0.997)	ENST00000324695
1008	R/C	rs199727860	2:2349050522:234905052	C/T	0	SNP	dbSNP	0	Missense variant	1deleterious(0)	905probably damaging(0.904)	ENST00000324695
1008	R/H	COSM1406478	2:2349050532:234905053	G/A	0	somatic_SNV	COSMIC	0	Missense variant	1deleterious(0)	815possibly damaging(0.814)	ENST00000324695
1013	F/L	TMP_ESP_2_234905069	2:2349050692:234905069	C/G	0	SNP	ESP	ESP,	Missense variant	371tolerated(0.37)	54benign(0.053)	ENST00000324695
1019	A/T	rs145966390	2:2349050852:234905085	G/A	0	SNP	dbSNP	Multiple_observations,Frequency,ESP,	Missense variant	21deleterious(0.02)	761possibly damaging(0.76)	ENST00000324695
1023	M/L	rs200703853	2:2349050972:234905097	A/T	0	SNP	dbSNP	0	Missense variant	1001tolerated(1)	1benign(0)	ENST00000324695
1023	M/I	TMP_ESP_2_234905099	2:2349050992:234905099	G/C	0	SNP	ESP	ESP,	Missense variant	31deleterious(0.03)	8benign(0.007)	ENST00000324695
1031	C/R	COSM1406479	2:2349051212:234905121	T/C	0	somatic_SNV	COSMIC	0	Missense variant	901tolerated(0.9)	540possibly damaging(0.539)	ENST00000324695
1033	C/S	rs201835578	2:2349051282:234905128	G/C	0	SNP	dbSNP	0	Missense variant	851tolerated(0.85)	8benign(0.007)	ENST00000324695
1037	N/S	TMP_ESP_2_234905140	2:2349051402:234905140	A/G	0	SNP	ESP	ESP,	Missense variant	901tolerated(0.9)	1benign(0)	ENST00000324695
1043	C/F	rs201096876	2:2349051582:234905158	G/T	0	SNP	dbSNP	0	Splice region variant, Missense variant	51deleterious(0.05)	993probably damaging(0.992)	ENST00000324695
1053	L/V	rs201193454	2:2349155232:234915523	C/G	0	SNP	dbSNP	0	Missense variant	61tolerated(0.06)	976probably damaging(0.975)	ENST00000324695
1054	A/T	rs202108535	2:2349155262:234915526	G/A	0	SNP	dbSNP	0	Missense variant	331tolerated(0.33)	132benign(0.131)	ENST00000324695
1063	Y/C	rs200883085	2:2349155542:234915554	A/G	0	SNP	dbSNP	0	Missense variant	1deleterious(0)	998probably damaging(0.997)	ENST00000324695
1071	A/V	rs201836188	2:2349155782:234915578	C/T	0	SNP	dbSNP	ESP,	Missense variant	291tolerated(0.29)	200benign(0.199)	ENST00000324695
1081	R/Q	rs139759512	2:2349167252:234916725	G/A	0	SNP	dbSNP	Multiple_observations,Frequency,ESP,	Missense variant	181tolerated(0.18)	10benign(0.009)	ENST00000324695
1081	R/Q	COSM175165	2:2349167252:234916725	G/A	0	somatic_SNV	COSMIC	0	Missense variant	181tolerated(0.18)	10benign(0.009)	ENST00000324695
1089	L/V	rs201188178	2:2349232062:234923206	C/G	0	SNP	dbSNP	0	Splice region variant, Missense variant	991tolerated(0.99)	3benign(0.002)	ENST00000324695

1101	N/S	rs144357618	2:2349232432:234923243	A/G	0	SNP	dbSNP	Multiple_observations,Frequency,ESP,	Missense variant	871tolerated(0.87)	1benign(0)	ENST00000324695
1104	K/E	rs201564611	2:2349232512:234923251	A/G	0	SNP	dbSNP	0	Missense variant	11deleterious (0.01)	2benign(0.001)	ENST00000324695
