# Supplementary material for: Expression of temperature-sensitive ion channel TRPM8 in sperm cells correlates with vertebrate evolution
Source: PeerJ. 2015 Oct 13;3:e1310. doi: 10.7717/peerj.1310 (PMC4614861; doi:10.7717/peerj.1310)
Supplement: Figure S1 — Invertebrates have TRPM-like genes but not authentic TRPM8 as demonstrated by Maximum-likelihood method based phylogenetic tree. Bootstrap = 1,000. [file peerj-03-1310-s003.docx]

**~~~~**

**Supplementary figure S1: TRPM-like genes are present in invertebrates.** Invertebrates have TRPM-like genes but not authentic TRPM8 as demonstrated by Maximum-likelihood method based phylogenetic tree. Bootstrap = 1000
