# Supplementary material for: Expression of temperature-sensitive ion channel TRPM8 in sperm cells correlates with vertebrate evolution
Source: PeerJ. 2015 Oct 13;3:e1310. doi: 10.7717/peerj.1310 (PMC4614861; doi:10.7717/peerj.1310)
Supplement: Figure S2 — (A) The epitome sequence of the anti-TRPM8 antibody used in this work is highly conserved in all species for which TRPM8 sequences are available. (B) The TRPM8 epitope sequence is missing in all other TRPM channels. [file peerj-03-1310-s004.docx]

**
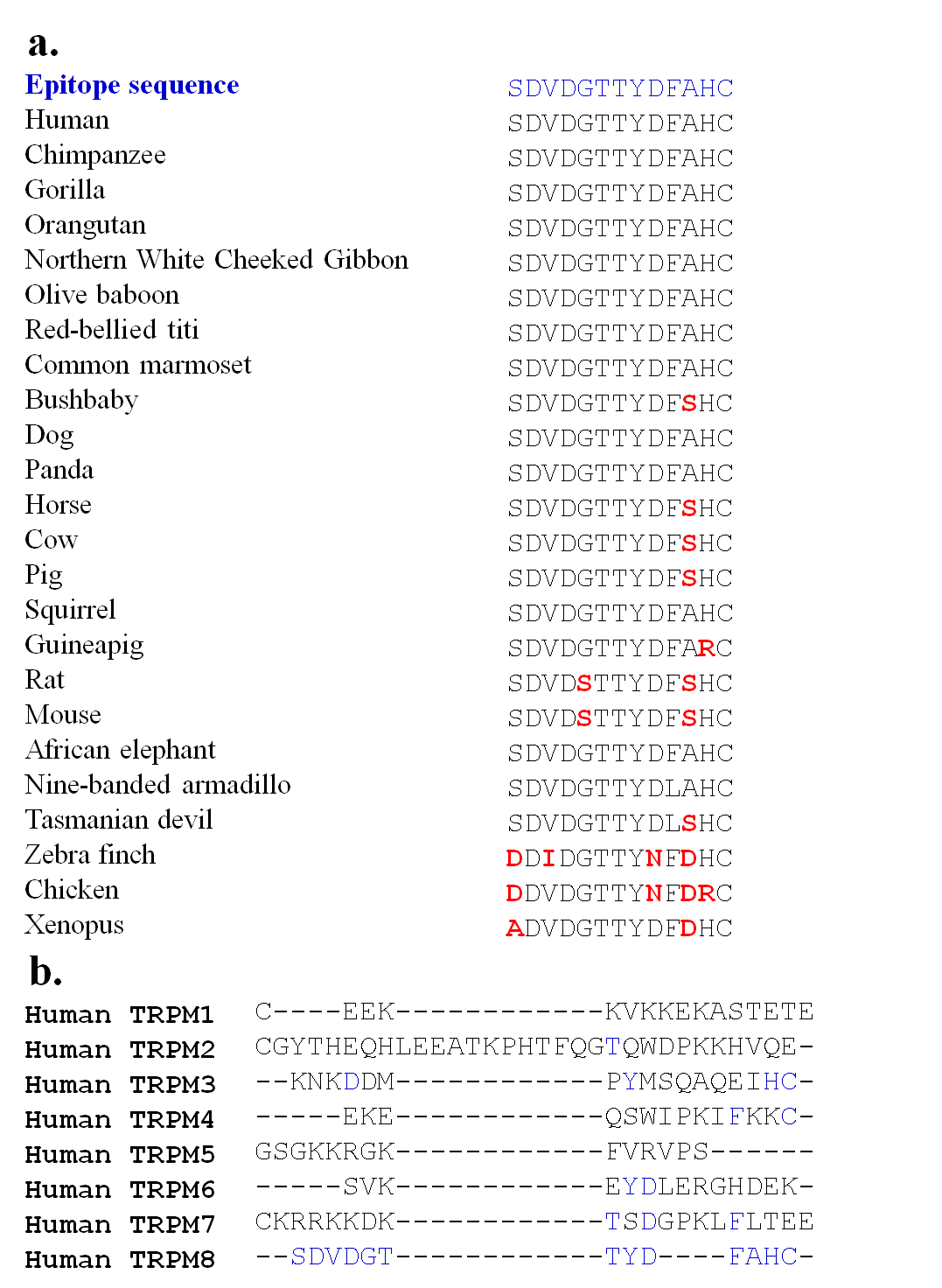
**

**Supplementary figure S2. Conservation of the epitope of the TRPM8-specific antibody. a.** The epitome sequence of the anti-TRPM8 antibody used in this work is highly conserved in all species for which TRPM8 sequences are available. **b.** The TRPM8 epitope sequence is missing in all other TRPM channels.
